# Chemoselective Hydrogenation of Nitroarenes Using an Air-Stable Base-Metal Catalyst

**DOI:** 10.1021/acs.orglett.1c00659

**Published:** 2021-03-23

**Authors:** Viktoriia Zubar, Abhishek Dewanji, Magnus Rueping

**Affiliations:** † Institute of Organic Chemistry, RWTH Aachen University, Landoltweg 1, 52074 Aachen, Germany; ‡ KAUST Catalysis Center (KCC), KAUST, Thuwal 23955-6900, Saudi Arabia

## Abstract

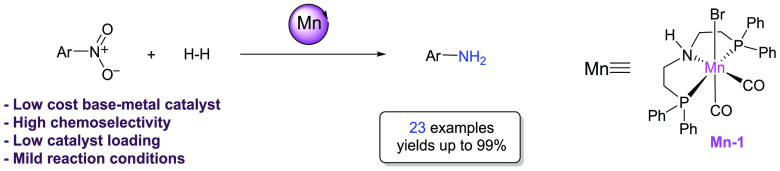

The reduction of nitroarenes to anilines as well as azobenzenes to hydrazobenzenes using a single base-metal catalyst is reported. The hydrogenation reactions are performed with an air-and moisture-stable manganese catalyst and proceed under relatively mild reaction conditions. The transformation tolerates a broad range of functional groups, affording aniline derivatives and hydrazobenzenes in high yields. Mechanistic studies suggest that the reaction proceeds via a bifunctional activation involving metal–ligand cooperative catalysis.

The reduction of nitroarenes to anilines represents one of the most significant reactions in organic chemistry. In this context, various procedures were developed to obtain anilines via the hydrogenation of nitroarenes.^1−3^ This straightforward approach features minimum waste generation because it gives water as the sole byproduct.^4^ The hydrogenation of nitroarenes also has great importance in industry due to the high demand for anilines for pharmaceuticals, dyes, agrochemical production, and polyurethanes synthesis. One of the commonly used reactions converting nitroarenes into anilines is Bechamp reduction.^5^ Despite a high functional group tolerance, this process exhibits considerable drawbacks, as it requires the use of corrosive hydrochloric acid and superstoichiometric amounts of iron or iron salts, leading to significant amounts of waste. Because of the high importance of substituted anilines, more economically beneficial methods are required. At present, the most commonly applied procedure is the catalytic hydrogenation of nitroarenes utilizing expensive Pd/C or pyrophoric Raney-Ni catalysts, which often suffer from low chemoselectivity. The desired chemoselectivity can be achieved by the modification of the standard catalysts. The application of modifiers usually decreases the reactivity as a result of the coverage of the active site and also involves laborious, complex, and not always reproducible preparation.^1^ Most of the recent reports for the catalytic hydrogenation of nitroarenes focus on the development and modification of heterogeneous catalysts.^1−3^ In contrast, only a few methods for the homogeneous reduction of nitroarenes are known. Given that homogeneous metal catalysts can be readily modified and adjusted by the application of different ligands or metals, higher chemoselectivity can often be realized. This is important for the synthesis of specific pharmaceuticals, which require high selectivity and low toxicity of the catalyst. For this reason a range of protocols were developed using homogeneous catalysts based on noble metals including Au,^6^ Ir,^7^ Pd,^6,8,9^ Pt,^8,10^ Rh,^7,11^ and Ru.^8,12−14^ Nevertheless, the replacement of noble-metal catalysts by earth-abundant alternatives is highly desirable in the context of sustainable chemistry. The first attempts to apply base-metal catalysts in the hydrogenation of nitroarenes to anilines were made by Knifton.^12^ Fe(CO)_3_(PPh_3_)_2_ and Fe(CO)_3_(AsPh_3_)_2_ were applied in low catalytic loading, leading to the selective formation of aniline under moderately mild conditions. Apart from this, Chaudhari et al. performed the hydrogenation of nitroarenes in aqueous/organic biphasic medium using an Fe/EDTANa_2_ system.^15^ The presence of a biphasic system allows a better separation of the product from the catalyst but also slows down the reaction. Good chemoselectivities were observed despite the use of a relatively high reaction temperature of 150 °C. In 2013, Beller and coworkers reported an iron-based complex for the catalytic hydrogenation of nitroarenes.^16^ The developed system operates under relatively mild reaction conditions and tolerates various functional groups. Here, up to 2 equiv of a strong acid, such as trifluoroacetic acid, was added to achieve significant catalyst activity.

Our interest in manganese catalysis^17^ and the lack of reports regarding its application in the hydrogenation of nitroarenes motivated us to address this issue. Recently, the application of manganese, as the third most abundant metal in the Earth’s crust, for the catalytic hydrogenation of organic molecules has considerably increased.^18−31^ To the best of our knowledge, no manganese-catalyzed reduction of nitroarenes to anilines has been previously reported, although manganese catalysis has been shown to be rather chemoselective.^1−3^ Hence we decided to explore a manganese-catalyzed reaction.

We started our investigation with the application of **Mn-1** catalyst, which can be easily prepared from a commercially available ligand and metal precursor and is able to activate molecular hydrogen, thereby being a powerful, inexpensive, and environmentally friendly reducing catalyst.

Nitrobenzene (**1a**) was chosen as model substrate to determine the optimal reaction conditions. Initially, we attempted to hydrogenate nitrobenzene using 5 mol % of **Mn-1** and 12.5 mol % of KO^*t*^Bu in toluene at 130 °C, applying 50 bar of H_2_ for 24 h. We were pleased to see that the **Mn-1** catalyst was active towards the reduction of nitrobenzene, producing the desired aniline (**2a**) in 59% GC yield (Table 1, entry 1). In the next step, other solvents were tested. The use of polar aprotic 1,4-dioxane resulted in a 44% yield of **2a** (Table 1, entry 2), whereas the application of *t*-amyl alcohol led to a 21% yield (Table 1, entry 3). Hence, with toluene as the solvent, we decided to investigate different bases. When Cs_2_CO_3_ was used for the activation of the catalyst, the yield dropped to 35%, whereas CsOH·H_2_O showed similar reactivity to KO^*t*^Bu, affording **2a** in 52% yield (Table 1, entries 4 and 5). To our delight, the use of cheap and readily available K_2_CO_3_ in the reaction led to the formation of aniline in 87% yield (Table 1, entry 6). Lastly, KH was tested; however, it provided an unsatisfactory result with a 27% yield of the desired product (Table 1, entry 7). Next, we increased the reaction temperature and pressure of H_2_ to 80 bar, which allowed us to reach a >99% yield of the aniline in both cases (Table 1, entries 8 and 9). As expected, the reaction did not take place in the absence of the catalyst or base (Table 1, entries 10 and 11).

**Table 1:**
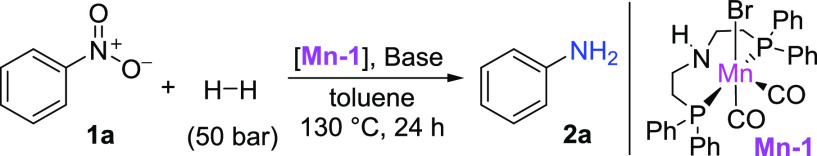
Optimization of the Reaction Conditions^a^

entry	solvent	base	yield (%)^b^
1	toluene	KO^*t*^Bu	59
2	1,4-dioxane	KO^*t*^Bu	44
3	TAA	KO^*t*^Bu	21
4	toluene	Cs_2_CO_3_	35
5	toluene	CsOH·H_2_O	52
6	toluene	K_2_CO_3_	87
7	toluene	KH	27
8^c^	toluene	K_2_CO_3_	>99
9^d^	toluene	K_2_CO_3_	>99
10^d^	toluene		<5
11^d^^,^^e^	toluene	K_2_CO_3_	nr

a Reaction conditions: nitrobenzene (0.25 mmol), **Mn-1** (5 mol %), base (12.5 mol %) in 1 mL of toluene at 130 °C under 50 bar of H_2_ for 24 h.

b Determined by the GC analysis using dodecane as an internal standard.

c 140 °C.

d 80 bar of H_2_.

e Without the catalyst. TAA, *t*-amyl alcohol.

With the optimized reaction conditions in hand, we started to explore the substrate scope for the hydrogenation of nitroarenes (Scheme 1). A range of alkyl-substituted nitroarenes were well tolerated and provided the corresponding anilines **2b**–**2e** in excellent yields of up to 97%. It should be noted that halogenated substrates were well tolerated, affording high yields of the desired aniline derivatives **2f**–**2i**. Remarkably, no protodehalogenation of the C–Hal bond took place, and a trifluoromethyl group in the meta position of the aromatic ring was also tolerated (**2j**). The developed system is able to chemoselectively reduce the nitro group in the presence of a double bond (**2k**), ester group (**2p**, **2q**), amide functionality (**2r**), and compounds containing a sulfonamide residue (**2u**). Other functional groups such as ether and thioether and the amino group were well tolerated, and the desired anilines (**2l**–**2o** and **2s**, **2t**) could be isolated in very high yields of up to 99%. Finally, 1-nitronaphthalene (**1v**) was successfully applied, providing naphthalen-1-amine (**2v**) in 75% yield. Unfortunately, under the general reaction conditions, nitriles, certain ketones, alkynes, and olefins are partially reduced.

**Scheme 1:**
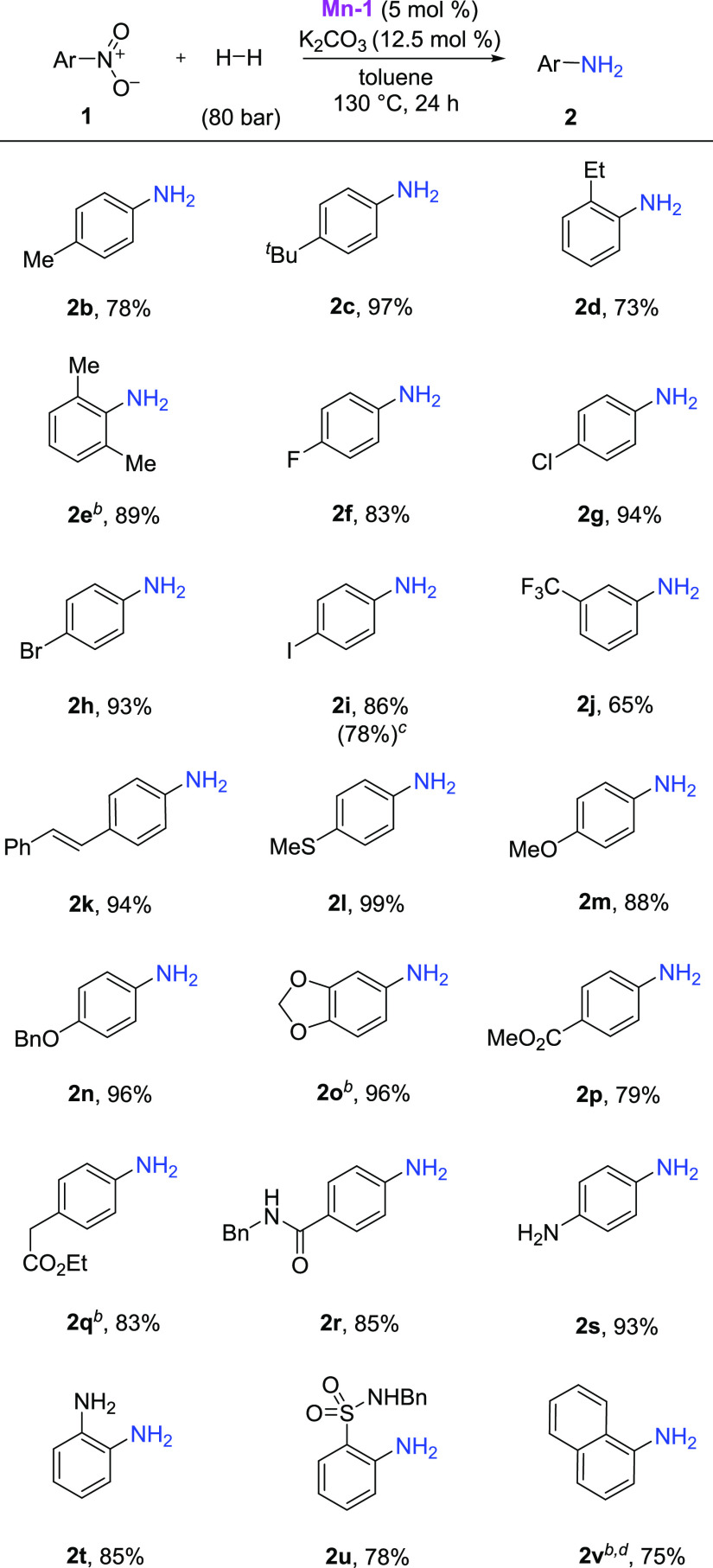
Selective Hydrogenation of Nitroarenes Catalyzed by **Mn-1** Reaction conditions: nitroarene (0.25 mmol), **Mn-1** (5 mol %), K_2_CO_3_ (12.5 mol %) in 1 mL of toluene at 130 °C under 80 bar of H_2_. Yields after isolation. **Mn-1** (10 mol %), K_2_CO_3_ (25 mol %). **1i** (1 g), **Mn-1** (4 mol %), K_2_CO_3_ (10 mol %) in 5 mL of toluene. 48 h.

Additionally, a gram-scale synthesis of 4-iodoaniline could also be performed using the optimized reaction conditions. The product **2i** was formed in 78% yield (Scheme 1), implying the feasibility of the described protocol. To show the general applicability of the developed method, we decided to perform the hydrogenation of **1w**, an intermediate in the synthesis of vortioxetine, an antidepressant used to treat major depressive disorder. A newly developed synthetic route includes the nucleophilic substitution of 2,4-dimethylbenzenethiol with 1-chloro-2-nitrobenzene to afford the desired intermediate **1w**.^32^ Under the optimized reaction conditions, the formed nitrophenylsulfane derivative **1w** undergoes catalytic hydrogenation, leading to the desired thioaniline derivative **2w** in 74% yield (Scheme 2). It is important to note that the transition metals are often inhibited by thio- and amino groups and result in reduced activity. Furthermore, several M(0) species generated in late transition-metal-catalyzed reactions undergo C–S-type oxidative additions that can be prevented by the use of manganese catalysts, such as **Mn-1**. The reaction of **2w** with 2-chloro-*N*-(2-chloroethyl)ethanamine hydrochloride then provides the desired vortioxetine.

**Scheme 2:**
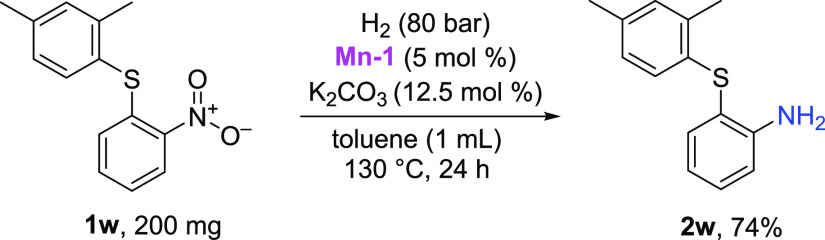
Synthesis of Vortioxetine Intermediate

There are two commonly studied pathways for the hydrogenation of nitroarenes to anilines. The first one is a direct pathway where the reduction proceeds *via* the formation of nitrosoarene and hydroxylamine intermediates. The second one occurs when an azoxy compound is formed by the condensation of nitrosoarene and hydroxylamine and later undergoes reduction to azo and hydrazo compounds (Scheme 3). To investigate the reaction mechanism of the studied catalytic system, possible intermediates were submitted to the standard reaction conditions. *N*-Phenylhydroxylamine (**3**), azobenzene (**4**), and 1,2-diphenylhydrazine (**5**) were tested (Scheme 4a). The reduction of *N*-phenylhydroxylamine led to the formation of 49% of aniline, whereas azobenzene and 1,2-diphenylhydrazine provided only 7 and 10% of aniline, respectively. These results suggested that the nitroarenes undergo direct hydrogenation in the presence of the developed catalytic system. In the case of the formation of the unwanted azo and hydrazo compounds, **Mn-1** can partially transform them into the desired anilines. It should be noted that we did not observe the accumulation of intermediates such as hydroxylamine or azo, hydrazo, and azoxy compounds. Furthermore, we did not observe the formation of nanoparticles, as the pincer complexes are rather stable under the applied conditions. (See the SI for details.)

**Scheme 3:**
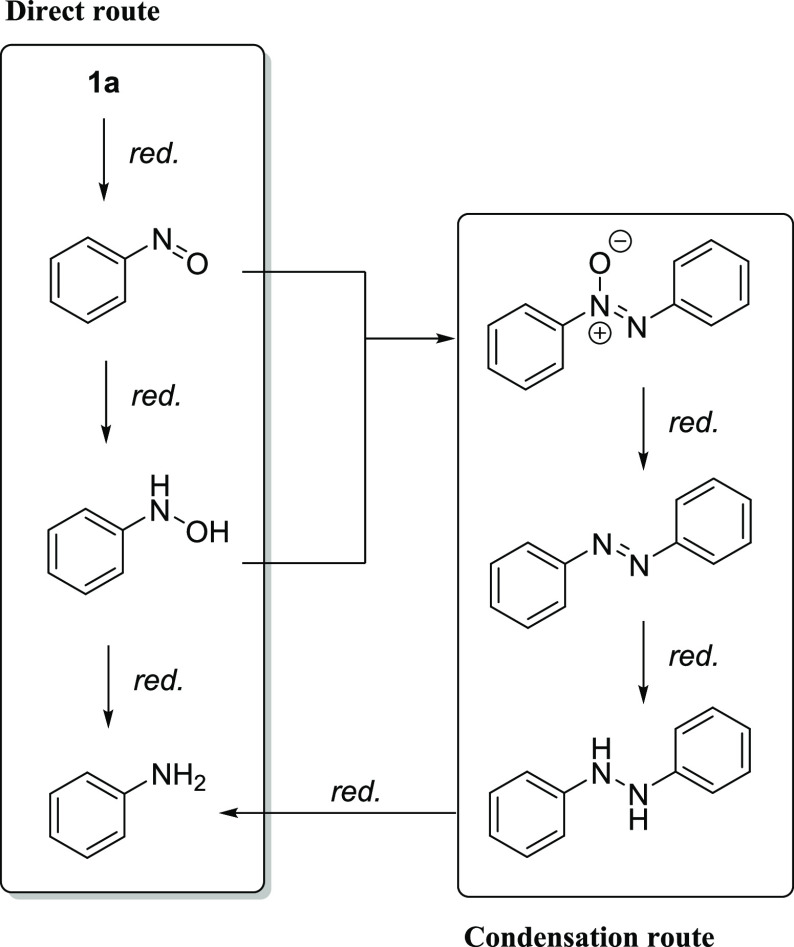
Possible Pathways for the Hydrogenation of Nitroarenes

**Scheme 4:**
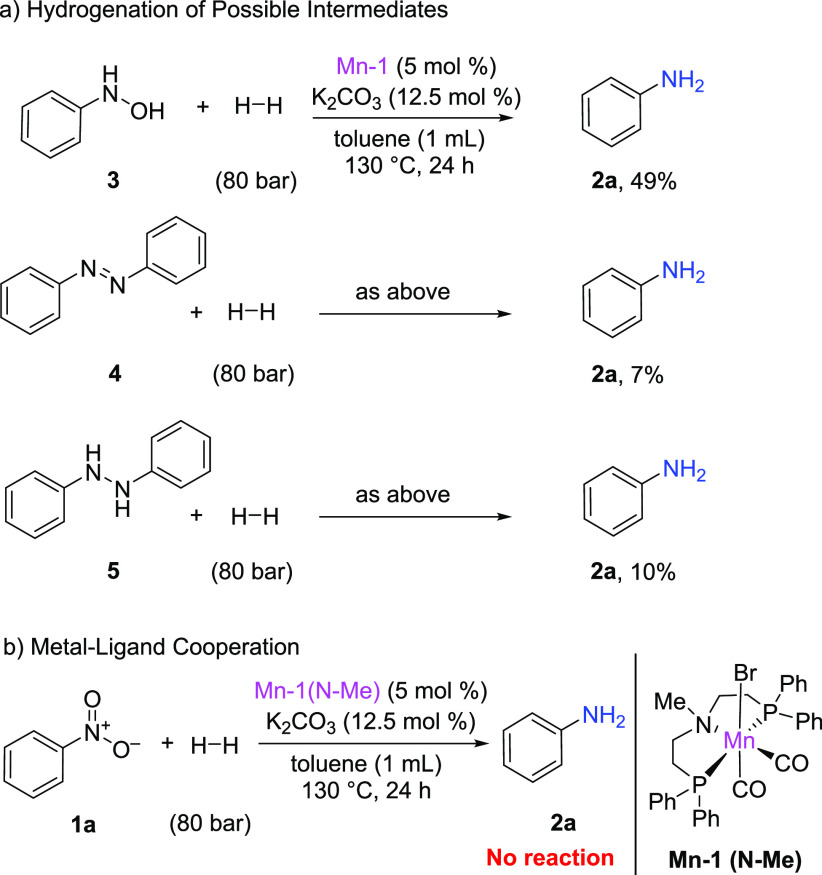
Mechanistic Studies

To prove whether the described reaction proceeds via metal–ligand cooperativity,^33^ we performed the hydrogenation reaction using the corresponding manganese **N-Me** derivative of **Mn-1** (Scheme 4b). As expected, the methylated complex **Mn-1** (**N-Me**) appeared to be inactive in the hydrogenation of nitrobenzene under the optimized reaction conditions, indicating that the presence of the N–H is critical for the reaction to proceed.

While performing our mechanistic studies, we observed the formation of a significant amount of hydrazobenzene when azobenzene was used as starting material. Taking this observation into account, we decided to optimize the reaction conditions for hydrazobenzenes synthesis due to the high importance of hydrazobenzenes in the synthesis of dyes and pharmaceuticals.^34^ The conditions appeared to be very close to those applied for nitroarene hydrogenation. The catalytic hydrogenation of azobenzene to hydrazobenzene was previously achieved using rare and expensive transition metals like Pd,^35^ Pt,^36^ and Ru.^37^

We were pleasantly surprised that our **Mn-1** catalyst was able to convert azobenzene to hydrazobenzene selectively, producing anilines as minor byproducts. Thus we decided to explore the substrate scope (Scheme 5). Substrates with electron-donating and electron-withdrawing substituents were tolerated, although in some cases, a higher catalyst loading of 7.5 mol % was required for the successful outcome of the reaction.

**Scheme 5:**
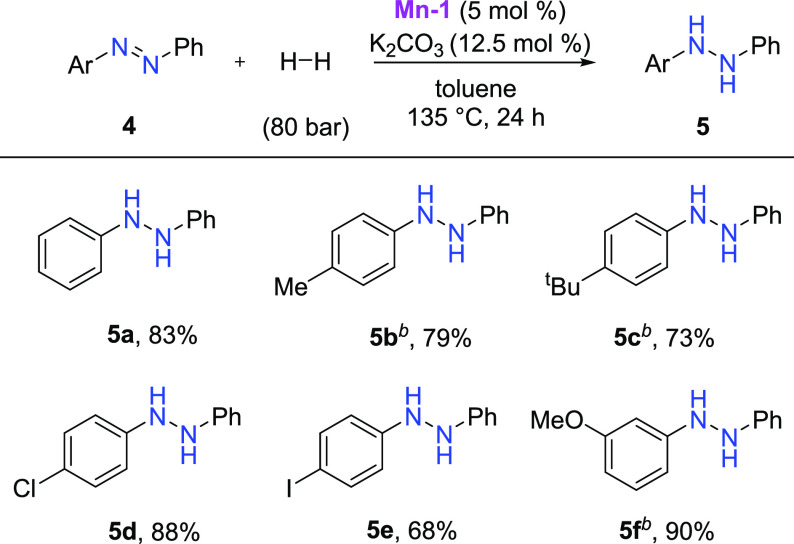
Selective Hydrogenation of Azobenzenes Catalyzed by **Mn-1** Reaction conditions: azobenzene (0.25 mmol), **Mn-1** (5 mol %), K_2_CO_3_ (12.5 mol %) in 1 mL of toluene at 135 °C under 80 bar of H_2_. Yields after isolation. **Mn-1** (7.5 mol %), K_2_CO_3_ (18.75 mol %).

In conclusion, a manganese-catalyzed protocol for the hydrogenation of nitroarenes was developed using molecular hydrogen as a reducing agent. The applied catalyst **Mn-1** can be synthesized from a commercially available manganese precursor and an air-stable and readily available PhPNP pincer ligand, highlighting the practicability of the developed protocol. The reaction proceeds under relatively mild conditions and provides the desired aniline derivatives in excellent yields. The newly developed manganese catalysis shows good reactivity and chemoselectivity and tolerates a variety of functional groups, leading to a practical protocol for the synthesis of anilines. Additionally, the hydrogenation of azobenzenes to hydrazobenzenes can be achieved, highlighting the high versatility of the developed catalytic system. The performed mechanistic studies suggested that the reaction takes place via metal–ligand cooperative catalysis and proceeds via a direct pathway to afford the desired anilines.

## Supporting Information Available

Detailed experimental procedures, characterization, and copies of ^1^H NMR and ^13^C NMR spectra of all products and synthesized starting materials (PDF)
