# Supplementary material for: Chemoselective Hydrogenation of Nitroarenes Using an Air-Stable Base-Metal Catalyst
Source: Org Lett. 2021 Mar 23;23(7):2742–7. doi: 10.1021/acs.orglett.1c00659 (PMC8041384; doi:10.1021/acs.orglett.1c00659)
Supplement: Supplementary file 1 — ol1c00659_si_001.pdf [file ol1c00659_si_001.pdf]

Supporting Information

Chemoselective Hydrogenation of Nitroarenes using an Air Stable Base Metal Catalyst

Viktoriia Zubar,[†] *Abhishek Dewanji*[†] *and Magnus Rueping**[†‡]

[†]Institute of Organic Chemistry, RWTH Aachen University, Landoltweg 1, 52074, Aachen, Germany
[‡]KAUST Catalysis Center (KCC), KAUST, Thuwal 23955-6900, Saudi Arabia

*Email: magnus.rueping@kaust.edu.sa
*@rwth-aachen.de

Table of Contents

General Information S3
Experimental Procedures and Characterization of the Products S4
NMR Spectra S15
Literature S46

General Information

All reactions were carried out under an argon atmosphere using oven-dried glassware. The dry and degassed tert-amyl alcohol was distilled from calcium hydride under nitrogen. DMF, toluene and DCM were obtained from MBRAUN Solvent Purification System. The dry and degassed dioxane was distilled from sodium benzophenone under nitrogen. All other chemicals were used as purchased without further purification. ^{1}H, ^{13}C, and ^{19}F spectra were recorded in $CDCl_3$ using Varian VNMR 600 MHz, Ascend Bruker 600 MHz, Ascend Bruker 400 MHz and Inova 400 MHz spectrometer. The signals were referenced to residual chloroform (7.26 ppm, ^{1}H, 77.00 ppm, ^{13}C). Chemical shifts are reported in ppm, multiplicities are indicated by br (broad signal), s (singlet), d (doublet), t (triplet), q (quartet), dd (doublet of doublets), td (triplet of doublets), tt (triplet of triplets) and m (multiplet). IR spectra were recorded on a Perkin Elmer-100 spectrometer and are reported in terms of frequency of absorption (cm-1). Mass spectra (EI-MS, 70 eV) were conducted on a Finnigan SSQ 7000 spectrometer. HRMS were recorded on a Thermo Scientific LTQ Orbitrap XL spectrometer. Analytical thin-layer chromatography (TLC) was performed using silica gel 60 pre-coated aluminium plates (Macherey-Nagel 0.20 mm thickness) with a fluorescent indicator UV254. Visualization was performed with standard phosphomolybdic acid stain (10g in 100 mL EtOH) or UV light. Analysis by gas chromatography (GC) was done using a CP-Sil-8-CB column (30 m, d = 0.25 mm) with FID detector and H_2 as carrier gas. **Mn-1** and **Mn-1 (N-Me)** catalysts were synthesized following our previous procedures.[1,2]

The high pressure laboratory reactor BR-300 (autoclave) used in this study was purchased from firma Berghof GmbH. For the hydrogenation reactions, precautions and safety measures are needed when working with flammable and compressed gases (see the general procedures for the hydrogenation of nitroarenes, azobenzenes and possible reaction intermediates). To be noted, appropriate care is required upon attempting to scale up the reactions, as these processes are exothermic.

Experimental Procedures and Characterization of the Products

Synthesis of the starting materials

All nitroarenes, if not otherwise noted, were purchased and used without further purification.

Compound **1n** was synthesized via alkylation of the corresponding phenol.

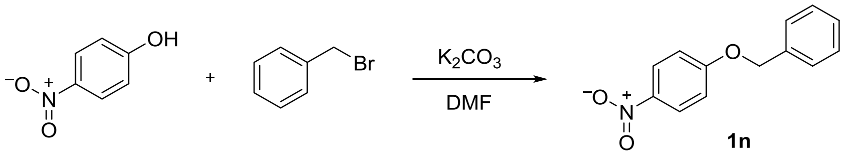

To a mixture of 4-nitrophenol (1.60 g, 11.5 mmol) and K_2CO_3 (2.38 g, 17.3 mmol, 1.5 equiv.) in DMF (10 mL) was added benzyl bromide dropwise at room temperature (1.4 ml, 12.7 mmol, 1.1 equiv.). Afterwards, the reaction was stirred at 80 °C in an oil bath for 12 h. After the completion of the reaction 20 ml of water were added and the product was extracted with EtOAc 3*25 mL. The combined organic layers were washed with brine, dried over sodium sulphate, filtered and concentrated under reduced pressure. The crude product was purified by column chromatography to give 1-(benzyloxy)-4-nitrobenzene **1n** (2.42 g, 92%).
Analytical data of the product are in agreement with those reported in the literature.[3] ^{1}**H NMR** (600 MHz, $CDCl_3$) δ 8.25 – 8.17 (m, 2H), 7.47 – 7.32 (m, 5H), 7.07 – 6.99 (m, 2H), 5.16 (s, 2H).

Compound **1r** was synthesized following the procedure described by Dahabiyeh, L.A. *et al.*[4]

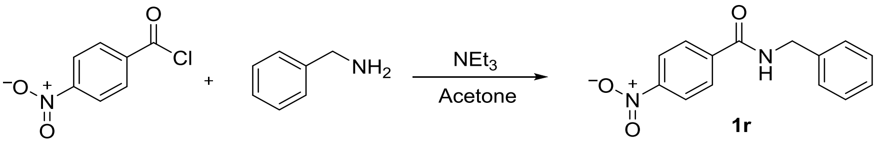

To a solution of benzyl amine (0.5 g, 4.67 mmol, 1 equiv.) and triethylamine (1.3 ml, 9.33 mmol, 2 equiv.) in dry acetone (30 mL) was added slowly 4-nitrobenzoyl chloride (1.73 g, 9.33 mmol, 2 equiv.) in acetone at 0 °C. The solution was allowed to stir at room temperature until completion (monitored by TLC). The reaction mixture was poured slowly into 5 % aqueous sodium bicarbonate solution. The crude product was extracted with EtOAc 3*20 ml. The combined organic layers were washed with brine and dried over sodium sulphate and concentrated under reduced pressure and purified by column chromatography to give *N*-benzyl-4-nitrobenzamide **1r** (0.93 g, 78%.)
Analytical data of the product are in agreement with those reported in the literature.[4] ^{1}**H NMR** (600 MHz, $CDCl_3$) δ 8.30 (d, J = 8.7 Hz, 2H), 7.97 (d, J = 8.7 Hz, 2H), 7.44 – 7.32 (m, 5H), 6.51 (s, 1H), 4.69 (d, J = 5.6 Hz, 2H).

Compound **1q** was synthesized by esterification of the corresponding acid.

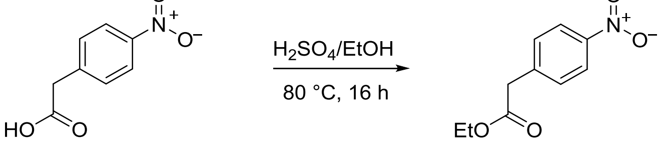

(4-Nitro-phenyl)-acetic acid (3 g, 16.4 mmol) was dissolved in ethanol (30 mL) and sulphuric acid (1 mL) was added carefully to the solution. The resulting reaction mixture was refluxed in an oil bath for 16 hours. Afterwards, the reaction mixture was neutralized with 2N aq. NaOH solution and extracted with EtOAc (3 × 50

mL). The combined organic layers were dried over sodium sulphate, filtered and evaporated in vacuo. Yield of (4-nitro-phenyl)-acetic acid ethyl ester is 84%, 2.9 g, light yellow solid.
Analytical data of the product are in agreement with those reported in the literature.[5] **^{1}H NMR** (600 MHz, $CDCl_3$) δ 8.20 – 8.17 (m, 2H), 7.46 – 7.45 (m, 2H), 4.17 (q, *J* = 7.1 Hz, 2H), 3.72 (s, 2H), 1.26 (t, *J* = 7.1 Hz, 3H).

Compound **1u** was synthesized following the procedure described by Uriac P. *et al.*[6]

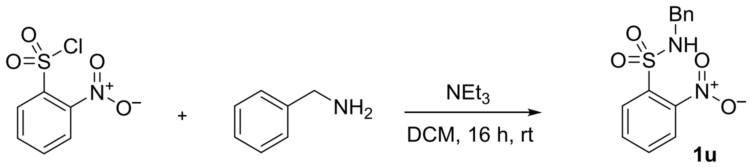

Benzylamine (1.86 mL, 17.1 mmol, 1.05 equiv.) was dissolved in dry DCM (30 mL), followed by the addition of Et_3N (4.53 mL, 32.5 mmol, 2 equiv.) and 2-nitrobenzenesulfonyl chloride (3.60 g, 16.2 mmol, 1 equiv.) at 0 °C. The mixture was allowed to come to room temperature and stirred for 16 h. The reaction mixture was washed with water (2x30 mL), a 10% aqueous solution of $NaHCO_3$ (2x15 mL), an aqueous solution of HCl 1 M (2x15 mL) and brine (2x30 mL). The organic layer was dried over Na_2SO_4, filtered and the solvent was removed under vacuum and the crude mixture was purified using column chromatography to yield **1u** (2.85 g, 60% yield).
Analytical data of the product are in agreement with those reported in the literature.[6] **^{1}H NMR** (600 MHz, $CDCl_3$) δ 8.03 (dd, *J* = 7.7, 1.3 Hz, 1H), 7.87 – 7.82 (m, 1H), 7.70 (td, *J* = 7.7, 1.4 Hz, 1H), 7.66 (td, *J* = 7.6, 1.1 Hz, 1H), 7.28 – 7.21 (m, 5H), 5.74 (t, *J* = 5.7 Hz, 1H), 4.35 (d, *J* = 6.3 Hz, 2H).

General procedure for the synthesis of azoarenes 4

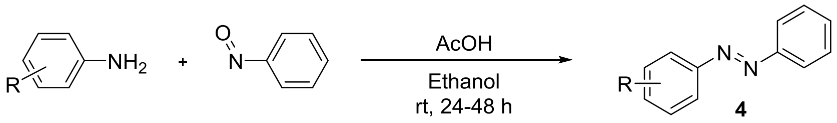

Nitrosobenzene (503 mg, 4.7 mmol, 1 equiv.) was taken in a round-bottomed flask and dissolved in ethanol (100 mL) by stirring. Acetic acid (1 mL) and the corresponding aniline (5.64 mmol, 1.2 equiv.) were added sequentially to the stirring solution of nitrosobenzene. The flask was then closed with a septum equipped with a needle (to avoid over-pressure) and the mixture was stirred at room temperature for 24-48 hours. The reaction was quenched with distilled water (80 mL) and extracted with DCM (2x100 mL). The combined organic phase was washed with brine solution (50 mL) and dried over anhydrous $MgSO_4$. The solvent was removed under reduced pressure and the crude mixture was purified by column chromatography.

(*E*)-1-phenyl-2-(*p*-tolyl)diazene, 4b

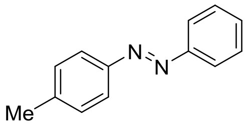

24 hrs; 770 mg, 83%, orange solid (pentane)
Analytical data of the product are in agreement with those reported in the literature.[7]
^{1}H NMR (400 MHz, $CDCl_3$) δ 7.91 (d, *J* = 7.4 Hz, 2H), 7.85 (d, *J* = 8.1 Hz, 2H), 7.54-7.50 (m, 2H), 7.48-7.44 (m, 1H), 7.32 (d, *J* = 8.0 Hz, 2H), 2.45 (s, 3H).

(*E*)-1-(4-(*tert*-butyl) phenyl)-2-phenyldiazene, 4c

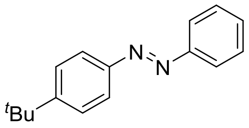

24 hrs; 390 mg, 35%, orange solid (pentane)
Analytical data of the product are in agreement with those reported in the literature.[7]
^{1}H NMR (400 MHz, $CDCl_3$) δ 7.93-7.86 (m, 4H), 7.56-7.44 (m, 5H), 1.39 (s, 9H).

(*E*)-1-(4-chlorophenyl)-2-phenyldiazene, 4d

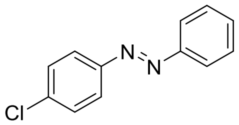

48 hrs; 340 mg, 33%, orange solid (pentane)

Analytical data of the product are in agreement with those reported in the literature.[8]

^{1}H NMR (400 MHz, $CDCl_3$) δ 7.93-7.87 (m, 4H), 7.55-7.47 (m, 5H).

(*E*)-1-(4-iodophenyl)-2-phenyldiazene, 4e

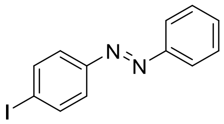

48 hrs; 346 mg, 24%, orange solid (pentane)

Analytical data of the product are in agreement with those reported in the literature.[7]

^{1}H NMR (400 MHz, $CDCl_3$) δ 7.93-7.90 (m, 2H), 7.89-7.85 (m, 2H), 7.68-7.64 (m, 2H), 7.55-7.49 (m, 3H).

(*E*)-1-(3-methoxyphenyl)-2-phenyldiazene, 4f

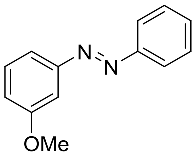

24 hrs; 300 mg, 30%, orange liquid (pentane / diethyl ether = 99:1)

Analytical data of the product are in agreement with those reported in the literature.[9]

^{1}H NMR (400 MHz, $CDCl_3$) δ 7.93 (d, *J* = 7.7 Hz, 2H), 7.58-7.40 (m, 6H), 7.05 (dd, *J* = 8.2, 2.7 Hz, 1H), 3.91 (s, 3H).

General procedure for the hydrogenation of nitroarenes

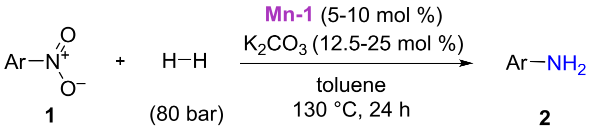

In an argon filled glovebox a 15 mL glass vial was charged with the corresponding nitroarene **1** (0.25 mmol), **Mn-1** (5-10 mol %), K_2CO_3 (12.5-25 mol %) and 1 mL of degassed toluene. The vial was sealed with a cap with a septum and was transferred into a stainless steel autoclave and a hole was made with a needle to allow the access of the gases. The autoclave was carefully flushed three times with nitrogen and then hydrogen gas. After adjusting the final hydrogen pressure to 80 bar, the autoclave was heated to 130 °C for 24 h with stirring. After cooling down the autoclave to room temperature the residual H_2 was carefully released and the mixture was analyzed by TLC. Next, the reaction mixture was purified by column chromatography on silica gel to obtain the pure aniline **2**.

Mn-1 catalyzed hydrogenation of 1-iodo-4-nitrobenzene (scaled up experiment)

In an argon filled glovebox a 15 mL glass vial was charged with 1-iodo-4-nitrobenzene **1j** (4.02 mmol, 1.0 g), **Mn-1** (5 mol %, 127 mg), K_2CO_3 (12.5 mol %, 69.4 mg) and 4 mL of degassed toluene. The vial was sealed with a cap with a septum and was transferred into a stainless steel autoclave and a hole was made with a needle to allow the access of the gases. The autoclave was carefully flushed three times with nitrogen and then hydrogen gas. After adjusting the final hydrogen pressure to 80 bar, the autoclave was heated to 130 °C for 24 h with stirring. After cooling down the autoclave to room temperature the residual H_2 was carefully released and the mixture was analyzed by TLC. Next, the reaction mixtures were purified by column chromatography on silica gel to obtain the pure 4-iodoaniline **2j** in 78% yield.

Mechanistic studies

Direct route

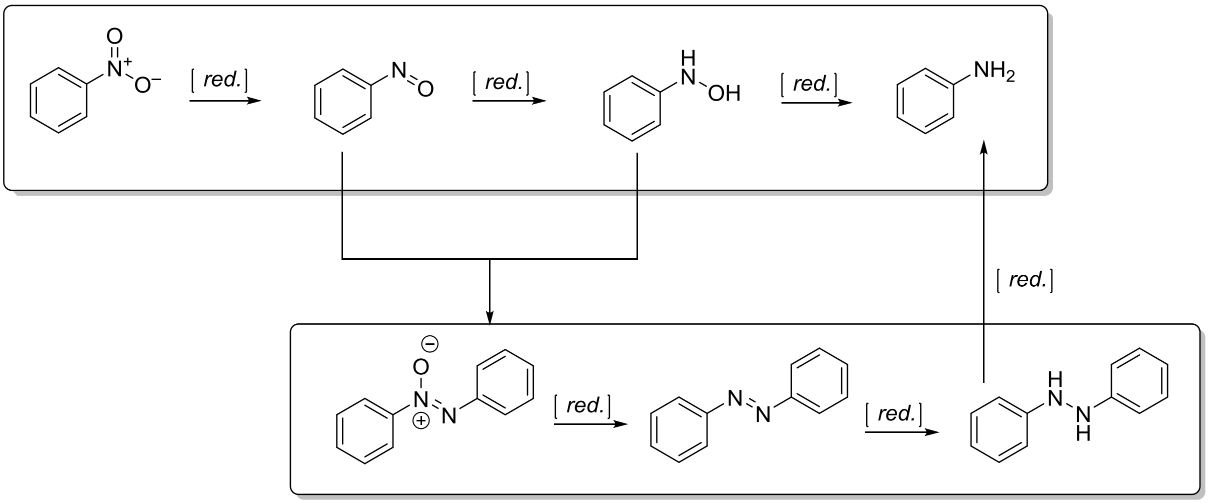

Condensation route

Scheme 1 Possible pathways for the hydrogenation of nitroarenes

General procedure for the hydrogenation of possible intermediates

In an argon filled glovebox a 15 mL glass vial was charged with the corresponding intermediate **3** (0.25 mmol), **4** (0.25 mmol) or **5** (0.25 mmol), **Mn-1** (5 mol %), K_2CO_3 (12.5 mol %) and 1 mL of degassed toluene. The vial was sealed with a cap with a septum and was transferred into a stainless steel autoclave and a hole was made with a needle to allow the access of the gases. The autoclave was carefully flushed three times with nitrogen and then hydrogen gas. After adjusting the final hydrogen pressure to 80 bar, the autoclave was heated to 130 °C for 24 h with stirring. After cooling down the autoclave to room temperature the residual H_2 was carefully released and the mixture was analyzed by GC. Yields of aniline were determined by the GC analysis using dodecane as internal standard.

General procedure for the hydrogenation of azobenzenes

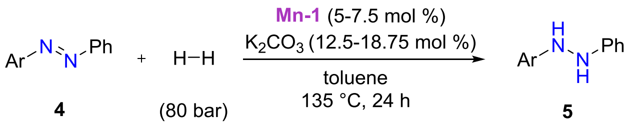

In an argon filled glovebox a 15 mL glass vial was charged with the corresponding azobenzene **4** (0.25 mmol), **Mn-1** (5-7.5 mol %), K_2CO_3 (12.5-18.75 mol %) and 1 mL of degassed toluene. The vial was sealed with a cap with a septum and was transferred into a stainless steel autoclave and a hole was made with a needle to allow the access of the gases. The autoclave was carefully flushed three times with nitrogen and then hydrogen gas. After adjusting the final hydrogen pressure to 80 bar, the autoclave was heated to 135 °C for 24 h with stirring. After cooling down the autoclave to room temperature the residual H_2 was carefully released and the mixture was analyzed by TLC. Next, the reaction mixture was purified by column chromatography on silica gel (pentan / ethyl acetate = 4:1 to 2:1) to obtain the pure hydrazobenzenes **4**.

Spectral data

p-Toluidine, 2b[10]

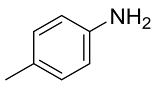

21 mg, 78%, dark brown solid (pentan / ethyl acetate = 4:1)

^{1}H NMR (600 MHz, $CDCl_3$) δ 6.89 (d, *J* = 8.0 Hz, 2H), 6.53 (d, *J* = 8.3 Hz, 2H), 3.35 (s, 2H), 2.16 (s, 3H).

^{13}C NMR (151 MHz, $CDCl_3$) δ 143.8, 129.8, 127.8, 115.3, 20.5.

4-(Tert-butyl)aniline, 2c[11]

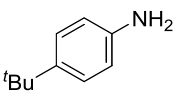

36 mg, 97%, dark brown solid (pentan / ethyl acetate = 4:1)

^{1}H NMR (600 MHz, $CDCl_3$) δ 7.23 – 7.19 (m, 2H), 6.70 – 6.64 (m, 2H), 3.68 (s, 2H), 1.30 (s, 9H).

^{13}C NMR (151 MHz, $CDCl_3$) δ 143.5, 141.6, 126.1, 115.1, 33.9, 31.5.

2-Ethylaniline, 2d[12]

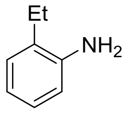

22 mg, 73%, brown oil (pentan / ethyl acetate = 4:1)

^{1}H NMR (600 MHz, $CDCl_3$) δ 7.08 (d, *J* = 7.5 Hz, 1H), 7.05 (td, *J* = 7.6, 1.4 Hz, 1H), 6.76 (td, *J* = 7.4, 1.0 Hz, 1H), 6.72 – 6.66 (m, 1H), 3.62 (s, 2H), 2.53 (q, *J* = 7.5 Hz, 2H), 1.26 (t, *J* = 7.6 Hz, 3H).

^{13}C NMR (151 MHz, $CDCl_3$) δ 144.0, 128.3, 128.1, 126.8, 118.8, 115.4, 24.0, 13.0.

2,6-Dimethylaniline, 2e[10]

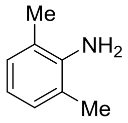

27 mg, 89%, brown oil (pentan / ethyl acetate = 4:1)

^{1}H NMR (400 MHz, $CDCl_3$) δ 6.87 (d, *J* = 7.4 Hz, 2H), 6.57 (t, *J* = 7.4 Hz, 1H), 3.50 (s, 2H), 2.11 (s, 6H).

^{13}C NMR (101 MHz, $CDCl_3$) δ 142.7, 128.2, 121.7, 118.0, 17.6.

4-Fluoroaniline, 2f[11]

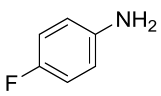

23 mg, 83%, dark brown solid (pentan / ethyl acetate = 4:1)

^{1}H NMR (600 MHz, $CDCl_3$) δ 6.89 – 6.82 (m, 2H), 6.65 – 6.59 (m, 2H), 3.54 (s, 2H).

^{13}C NMR (151 MHz, $CDCl_3$) δ 156.4 (d, C-F, $^{1}J_{C\text{-}F}$ = 235.8 Hz), 142.4 (d, C-F, $^{4}J_{C\text{-}F}$ = 1.5 Hz), 116.1 (d, C-F, $^{3}J_{C\text{-}F}$ = 7.6 Hz), 115.7 (d, C-F, $^{2}J_{C\text{-}F}$ = 22.8 Hz).

^{19}F NMR (564 MHz, $CDCl_3$) δ -126.85 (tt, *J* = 8.7, 4.5 Hz).

4-Chloroaniline, 2g[10]

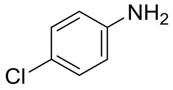

30 mg, 94%, grey solid (pentan / ethyl acetate = 4:1)

^{1}H NMR (600 MHz, $CDCl_3$) δ 7.14 – 7.06 (m, 2H), 6.63 – 6.59 (m, 2H), 3.59 (s, 2H).

^{13}C NMR (151 MHz, $CDCl_3$) δ 144.9, 129.1, 123.2, 116.3.

4-Bromoaniline, 2h[10]

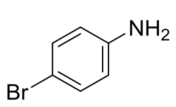

40 mg, 93%, dark brown solid (pentan / ethyl acetate = 4:1)

^{1}H NMR (600 MHz, $CDCl_3$) δ 7.31 – 7.22 (m, 2H), 6.65 – 6.48 (m, 2H), 3.68 (s, 2H).

^{13}C NMR (151 MHz, $CDCl_3$) δ 145.5, 132.0, 116.7, 110.2.

4-Iodoaniline, 2i[13]

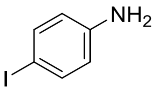

47 mg, 86%, dark brown solid (pentan / ethyl acetate = 4:1)

^{1}H NMR (600 MHz, $CDCl_3$) δ 7.43 – 7.38 (m, 2H), 6.50 – 6.44 (m, 2H), 3.68 (s, 2H).

^{13}C NMR (151 MHz, CDCl3) δ 146.1, 137.9, 117.3, 79.4.

3-(trifluoromethyl)aniline, 2j[14]

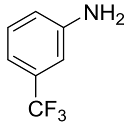

26 mg, 65%, yellow oil (pentan / ethyl acetate = 4:1)

^{1}H NMR (600 MHz, $CDCl_3$) δ 7.27 (t, J = 7.9 Hz, 1H), 7.02 (d, J = 7.7 Hz, 1H), 6.92 (s, 1H), 6.84 (dd, J = 8.0, 1.8 Hz, 1H), 3.85 (s, 2H).

^{13}C NMR (151 MHz, $CDCl_3$) δ 146.7, 131.6 (q, C-F, $^2J_{C\text{-}F}$ = 31.8 Hz), 129.7, 124.2 (q, C-F, $^1J_{C\text{-}F}$ = 272.2 Hz), 118.0, 115.0 (q, C-F, $^3J_{C\text{-}F}$ = 4.0 Hz), 111.3 (q, C-F, $^3J_{C\text{-}F}$ = 3.9 Hz).

^{19}F NMR (565 MHz, $CDCl_3$) δ -62.9.

(E)-4-Styrylaniline, 2k[15]

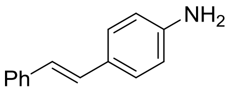

46 mg, 94%, beige solid (pentan / ethyl acetate = 4:1)

^{1}H NMR (400 MHz, $CDCl_3$) δ 7.47 (d, J = 7.7 Hz, 2H), 7.35-7.31 (m, 4H), 7.21 (t, J = 7.4 Hz, 1H), 7.03 (d, J = 16.3 Hz, 1H), 6.92 (d, J = 16.3 Hz, 1H), 6.68 (d, J = 8.4 Hz, 2H), 3.73 (s, 2H).

^{13}C NMR (101 MHz, $CDCl_3$) δ 146.1, 137.9, 128.7, 128.6, 128.0, 127.7, 126.9, 126.1, 125.1, 115.2.

4-(Methylthio)aniline, 2l[11]

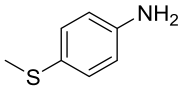

34.5 mg, 99%, dark brown solid (pentan / ethyl acetate = 4:1)

^{1}H NMR (600 MHz, $CDCl_3$) δ 7.19 (s, 2H), 6.63 (s, 2H), 3.75 (s, 2H), 2.42 (s, 3H).

^{13}C NMR (151 MHz, $CDCl_3$) δ 145.3, 131.0, 125.8, 115.9, 18.8.

4-Methoxyaniline, 2m[10]

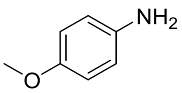

27 mg, 88%, grey solid (pentan / ethyl acetate = 4:1)

^{1}H NMR (600 MHz, $CDCl_3$) δ 6.77 – 6.73 (m, 2H), 6.67 – 6.63 (m, 2H), 3.75 (s, 3H), 3.32 (s, 2H).

^{13}C NMR (151 MHz, $CDCl_3$) δ 152.8, 139.9, 116.4, 114.8, 55.7.

4-(Benzyloxy)aniline, 2n[10]

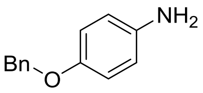

48 mg, 96%, brown solid (pentan / ethyl acetate = 4:1)

^{1}H NMR (400 MHz, $CDCl_3$) δ 7.47 – 7.27 (m, 5H), 6.82 (d, J = 8.8 Hz, 2H), 6.64 (d, J = 8.8 Hz, 2H), 4.99 (s, 2H), 3.43 (s, 2H).

^{13}C NMR (101 MHz, $CDCl_3$) δ 152.0, 140.2, 137.5, 128.5, 127.8, 127.5, 116.4, 116.1, 70.8.

Benzo[d][1,3]dioxol-5-amine, 2o[11]

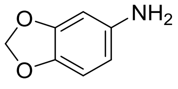

33 mg, 96%, dark brown solid (pentan / ethyl acetate = 4:1)

^{1}H NMR (600 MHz, $CDCl_3$) δ 6.62 (d, J = 8.2 Hz, 1H), 6.29 (d, J = 2.3 Hz, 1H), 6.13 (dd, J = 8.2, 2.3 Hz, 1H), 5.86 (s, 2H), 3.47 (s, 2H).

^{13}C NMR (151 MHz, $CDCl_3$) δ 148.2, 141.4, 140.3, 108.6, 106.9, 100.7, 98.1.

Methyl 4-aminobenzoate, 2p[13]

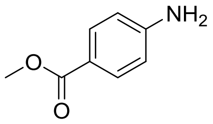

30 mg, 79%, beige solid (pentan / ethyl acetate = 4:1)

^{1}H NMR (400 MHz, $CDCl_3$) δ 7.84 (d, *J* = 8.6 Hz, 2H), 6.63 (d, *J* = 8.6 Hz, 2H), 4.07 (s, 2H), 3.85 (s, 3H).

^{13}C NMR (101 MHz, $CDCl_3$) δ 167.2, 150.9, 131.6, 119.7, 113.8, 51.6.

Ethyl 2-(4-aminophenyl)acetate, 2q[16]

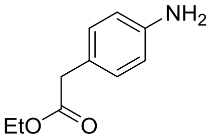

37 mg, 83%, brown oil (pentan / ethyl acetate = 4:1)

^{1}H NMR (600 MHz, $CDCl_3$) δ 6.99 (d, *J* = 8.3 Hz, 2H), 6.56 (d, *J* = 8.3 Hz, 2H), 4.05 (q, *J* = 7.1 Hz, 2H), 3.55 (s, 2H), 3.41 (s, 2H), 1.16 (t, *J* = 7.1 Hz, 3H).

^{13}C NMR (151 MHz, $CDCl_3$) δ 172.2, 145.4, 130.1, 124.0, 115.3, 60.7, 40.6, 14.2.

4-Amino-N-benzylbenzamide, 2r[17]

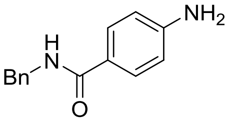

48 mg, 85%, beige solid (pentan / ethyl acetate = 2:1)

^{1}H NMR (600 MHz, $CDCl_3$) δ 7.62 (d, *J* = 8.1 Hz, 2H), 7.34 (d, *J* = 4.3 Hz, 4H), 7.30-7.27 (m, 1H), 6.65 (d, *J* = 8.1 Hz, 2H), 6.29 (s, 1H), 4.62 (d, *J* = 5.5 Hz, 2H), 3.98 (s, 2H).

^{13}C NMR (151 MHz, $CDCl_3$) δ 149.6, 138.6, 128.7, 127.9, 127.5, 123.9, 114.2, 44.0.

Benzene-1,4-diamine, 2s[10]

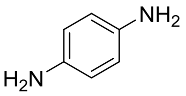

25 mg, 93%, dark brown solid (pentan / ethyl acetate = 2:1)

^{1}H NMR (400 MHz, $CDCl_3$) δ 6.56 (s, 4H), 3.28 (s, 4H).

^{13}C NMR (101 MHz, $CDCl_3$) δ 138.6, 116.7.

Benzene-1,2-diamine, 2t[18]

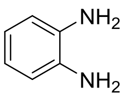

23 mg, 85%, grey solid (pentan / ethyl acetate = 2:1)

^{1}H NMR (600 MHz, $CDCl_3$) δ 6.63 (s, 4H), 3.38 (s, 4H).

^{13}C NMR (151 MHz, $CDCl_3$) δ 134.9, 120.3, 116.9.

2-Amino-N-benzylbenzenesulfonamide, 2u[19]

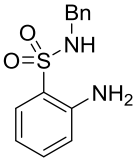

51 mg, 78%, brown oil (pentan / ethyl acetate = 2:1)

^{1}H NMR (600 MHz, $CDCl_3$) δ 7.75 (dd, *J* = 8.0, 1.4 Hz, 1H), 7.37 – 7.33 (m, 1H), 7.31 – 7.24 (m, 3H), 7.23 – 7.18 (m, 2H), 6.85 – 6.81 (m, 1H), 6.78 (d, *J* = 8.1 Hz, 1H), 5.10 (t, *J* = 5.7 Hz, 1H), 4.87 (s, 2H), 4.06 (d, *J* = 6.2 Hz, 2H).

^{13}C NMR (151 MHz, $CDCl_3$) δ 145.1, 136.3, 134.3, 129.8, 128.7, 127.9(89), 127.9(88), 121.5, 117.9, 117.8, 47.3.

Naphthalen-1-amine, 2v[10]

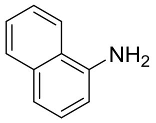

27 mg, 75%, dark brown solid (pentan / ethyl acetate = 4:1)

^{1}H NMR (400 MHz, $CDCl_3$) δ 7.76 – 7.68 (m, 2H), 7.42 – 7.33 (m, 2H), 7.26 – 7.14 (m, 2H), 6.69 (dd, *J* = 6.8, 1.5 Hz, 1H), 4.02 (s, 2H).

^{13}C NMR (101 MHz, $CDCl_3$) δ 142.1, 134.4, 128.6, 126.4, 125.9, 124.9, 123.7, 120.8, 119.0, 109.7.

Synthesis of 2-((2,4-dimethylphenyl)thio)aniline (**2w**) was performed following the procedure described by M. M. Rathore[20]

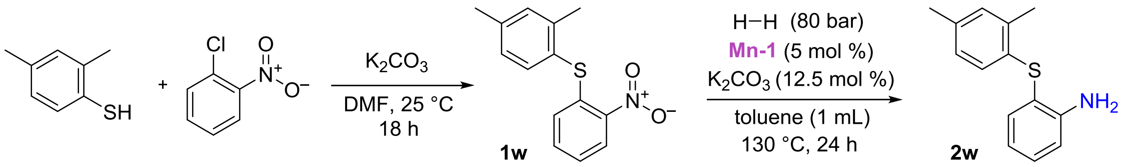

(2,4-dimethylphenyl)(2-nitrophenyl)sulfane (**1w**): 2,4-dimethylbenzenethiol (1.8 g, 13.3 mmol) was added to a stirred solution of 1-chloro-2-nitrobenzene (2.0 g, 12.7 mmol) and K_2CO_3 (2.1 g, 15.2 mmol) in 10 mL of dry DMF, afterwards the reaction mixture was stirred at room temperature for 18 h. 30 mL of water was added to the reaction mixture and was further stirred at room temperature for 30 min. The formed yellow precipitate was then filtered off, washed several times with water and dried to afford the title compound **1w** as yellow solid.
Yield: 3.2 g, 97 %. **^{1}H NMR** (600 MHz, $CDCl_3$) δ 8.25 (dd, *J* = 8.3, 1.3 Hz, 1H), 7.47 (d, *J* = 7.8 Hz, 1H), 7.32-7.29 (m, 1H), 7.23 – 7.15 (m, 2H), 7.11 (d, *J* = 7.7 Hz, 1H), 6.70 (dd, *J* = 8.2, 1.1 Hz, 1H), 2.39 (s, 3H), 2.30 (s, 3H). **^{13}C NMR** (151 MHz, $CDCl_3$) δ 144.9, 143.1, 141.0, 139.3, 137.2, 133.5, 132.2, 128.4, 127.3, 126.3, 126.0, 124.5, 21.3, 20.4.
2-((2,4-dimethylphenyl)thio)aniline (**2w**): In an argon filled glovebox a 15 mL glass vial was charged with (2,4-dimethylphenyl)(2-nitrophenyl)sulfane (**1w**) (0,77 mmol, 200 mg), **Mn-1** (5 mol%, 24,4 mg), K_2CO_3 (12,5 mol %, 13,3 mg) and 1 mL of degassed toluene. The vial was sealed with a cap with a septum and was transferred into a stainless steel autoclave and a hole was made with a needle to allow the access of the gases. The autoclave was carefully flushed three times with nitrogen and then hydrogen gas. After adjusting the final hydrogen pressure to 80 bar, the autoclave was heated to 130 °C for 24 h with stirring. After cooling down the autoclave to room temperature the residual H_2 was carefully released and the mixture was analyzed by TLC. Next, the reaction mixtures were purified by column chromatography on silica gel (pentan / ethyl acetate = 4:1) to obtain the 2-((2,4-dimethylphenyl)thio)aniline **2w** in 74% yield. **^{1}H NMR** (600 MHz, $CDCl_3$) δ 7.40 (dd, *J* = 7.7, 1.5 Hz, 1H), 7.28 – 7.23 (m, 1H), 7.04 (s, 1H), 6.89 (d, *J* = 8.0 Hz, 1H), 6.82 (dd, *J* = 8.0, 1.1 Hz, 1H), 6.79 (td, *J* = 7.5, 1.3 Hz, 1H), 6.75 (d, *J* = 8.0 Hz, 1H), 4.25 (s, 2H), 2.44 (s, 3H), 2.31 (s, 3H). **^{13}C NMR** (151 MHz, $CDCl_3$) δ 148.4, 136.6, 135.8, 135.4, 131.8, 131.2, 130.5, 127.4, 126.6, 118.9, 115.3, 115.2, 20.8, 20.1.

1,2-Diphenylhydrazine, 5a[21]

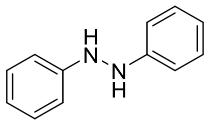

38 mg, 83%, white solid (pentan / ethyl acetate = 9:1)
^{1}H NMR (400 MHz, $CDCl_3$) δ 7.26 – 7.19 (m, 4H), 6.88-6.82 (m, 6H), 5.59 (br, 2H).
^{13}C NMR (101 MHz, $CDCl_3$) δ 148.8, 129.3, 119.9, 112.3.

1-Phenyl-2-(p-tolyl)hydrazine, 5b[22]

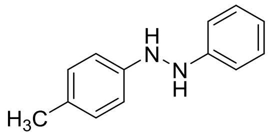

39 mg, 79%, white solid (pentan / ethyl acetate = 9:1)
^{1}H NMR (400 MHz, $CDCl_3$) δ 7.23 – 7.16 (m, 2H), 7.01 (d, *J* = 8.3 Hz, 2H), 6.86-6.79 (m, 3H), 6.75 (d, *J* = 8.4 Hz, 2H), 5.55 (d, *J* = 24.2 Hz, 2H), 2.25 (s, 3H).
^{13}C NMR (101 MHz, $CDCl_3$) δ 149.0, 146.5, 129.8, 129.3, 129.2, 119.8, 112.4, 112.3, 20.5.

1-(4-(*tert*-Butyl)phenyl)-2-phenylhydrazine, 5c[23]

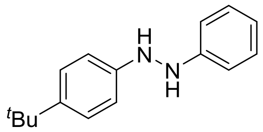

44 mg, 73%, white solid (pentan / ethyl acetate = 9:1)

^{1}H NMR (400 MHz, $CDCl_3$) δ 7.27 – 7.17 (m, 4H), 6.91 – 6.76 (m, 5H), 5.55 (d, *J* = 8.9 Hz, 2H), 1.28 (s, 9H).

^{13}C NMR (101 MHz, $CDCl_3$) δ 149.1, 146.5, 142.8, 129.3, 126.1, 119.8, 112.3, 112.1, 34.0, 31.5.

1-(4-Chlorophenyl)-2-phenylhydrazine, 5d[22]

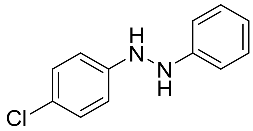

48 mg, 88%, white solid (pentan / ethyl acetate = 9:1)

^{1}H NMR (600 MHz, $CDCl_3$) δ 7.25-7.21 (m, 2H), 7.19 – 7.15 (m, 2H), 6.89 – 6.85 (m, 1H), 6.84-6.79 (m, 4H), 5.61 (d, *J* = 9.9 Hz, 2H).

^{13}C NMR (151 MHz, $CDCl_3$) δ 148.5, 147.5, 129.4, 129.3, 124.4, 120.2, 113.5, 112.4.

1-(4-Iodophenyl)-2-phenylhydrazine, 5e[24]

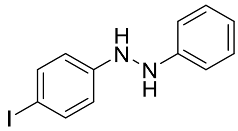

53 mg, 68%, white solid (pentan / ethyl acetate = 9:1)

^{1}H NMR (600 MHz, $CDCl_3$) δ 7.50 – 7.46 (m, 2H), 7.25 – 7.20 (m, 2H), 6.89 – 6.84 (m, 1H), 6.83 – 6.79 (m, 2H), 6.68 – 6.63 (m, 2H), 5.61 (br, 2H).

^{13}C NMR (151 MHz, $CDCl_3$) δ 148.6, 148.3, 138.0, 129.4, 120.3, 114.5, 112.4, 81.0.

1-(3-Methoxyphenyl)-2-phenylhydrazine, 5f

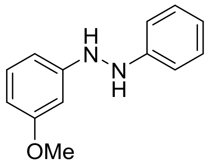

48 mg, 90%, yellow oil (pentan / ethyl acetate = 9:1)

^{1}H NMR (400 MHz, $CDCl_3$) δ 7.23 (t, *J* = 7.9 Hz, 2H), 7.16 – 7.10 (m, 1H), 6.89 – 6.81 (m, 3H), 6.49 – 6.38 (m, 3H), 5.60 (d, *J* = 2.8 Hz, 2H), 3.76 (s, 3H).

^{13}C NMR (101 MHz, $CDCl_3$) δ 160.9, 150.4, 148.8, 130.2, 129.3, 119.9, 112.4, 105.1, 105.0, 98.3, 55.1.

IR (ATR) ν (cm-1): 3349, 2937, 2326, 2087, 1840, 1597, 1488, 1284, 1255, 1202, 1156, 1039, 838, 755, 689.

HRMS (ESI) m/z: $[M+H]^+$: Calc. for $C_{13}H_{15}N_2O$ 215.1179; Found 215.1174.

In order to get an idea about the stability of the catalyst we conducted the NMR experiment of the following reaction:

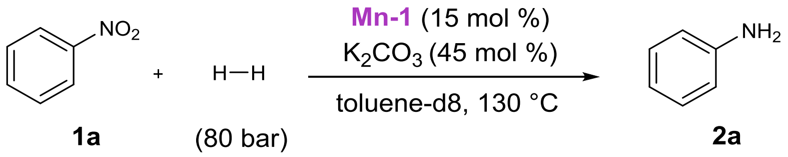

before the reaction

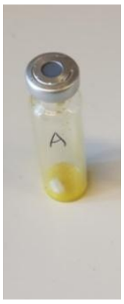

after the reaction

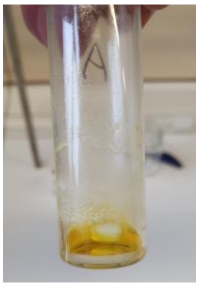

NMR spectra after reaction:

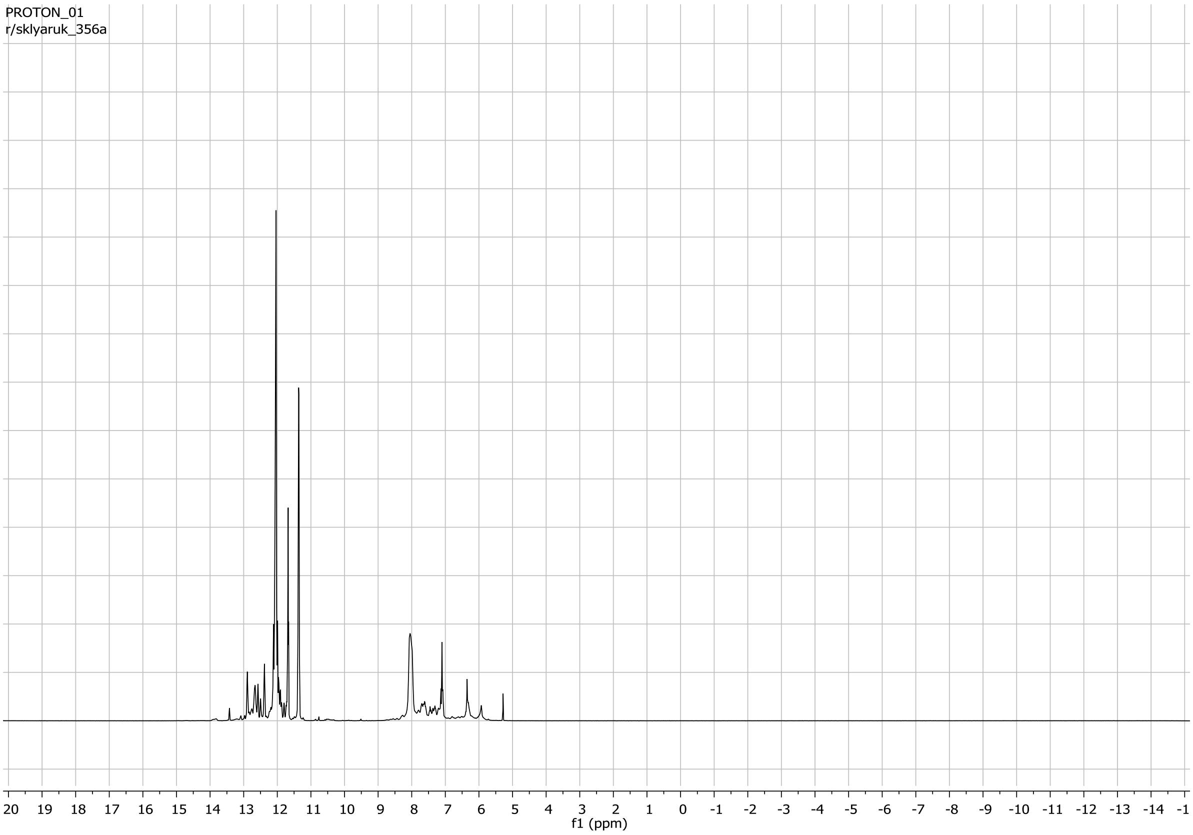

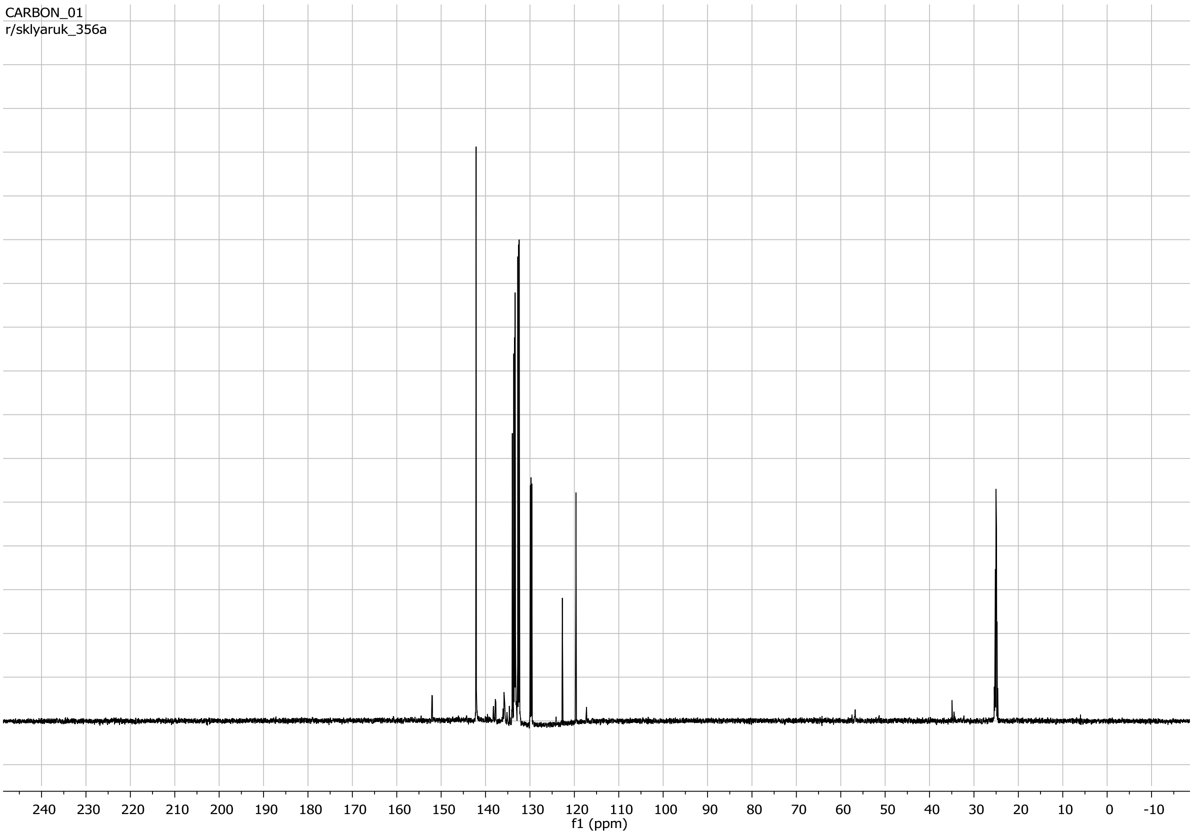

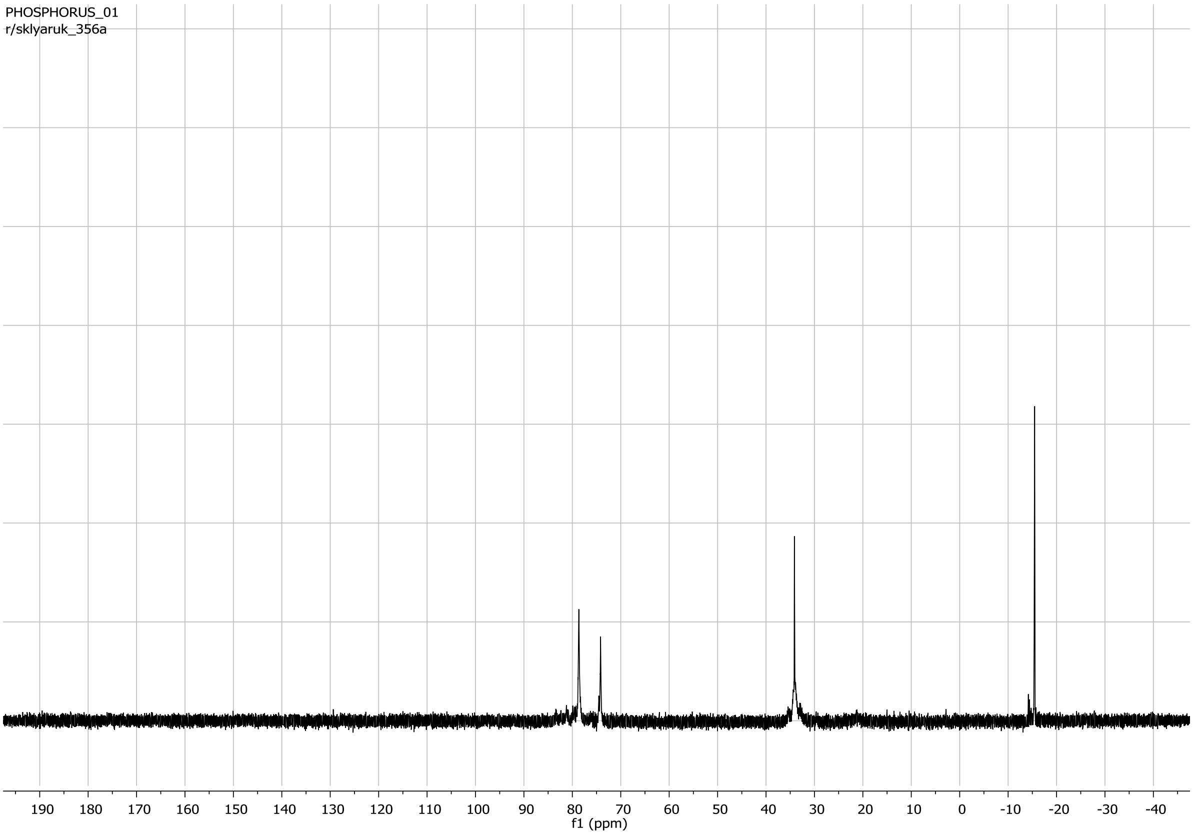

The NMR-spectra indicate that the complex is still present. However, more experiments need to be conducted in order to fully characterize the catalyst after the reaction.

NMR Spectra

^{1}H NMR spectra of **2b** ($CDCl_3$, 600 MHz)

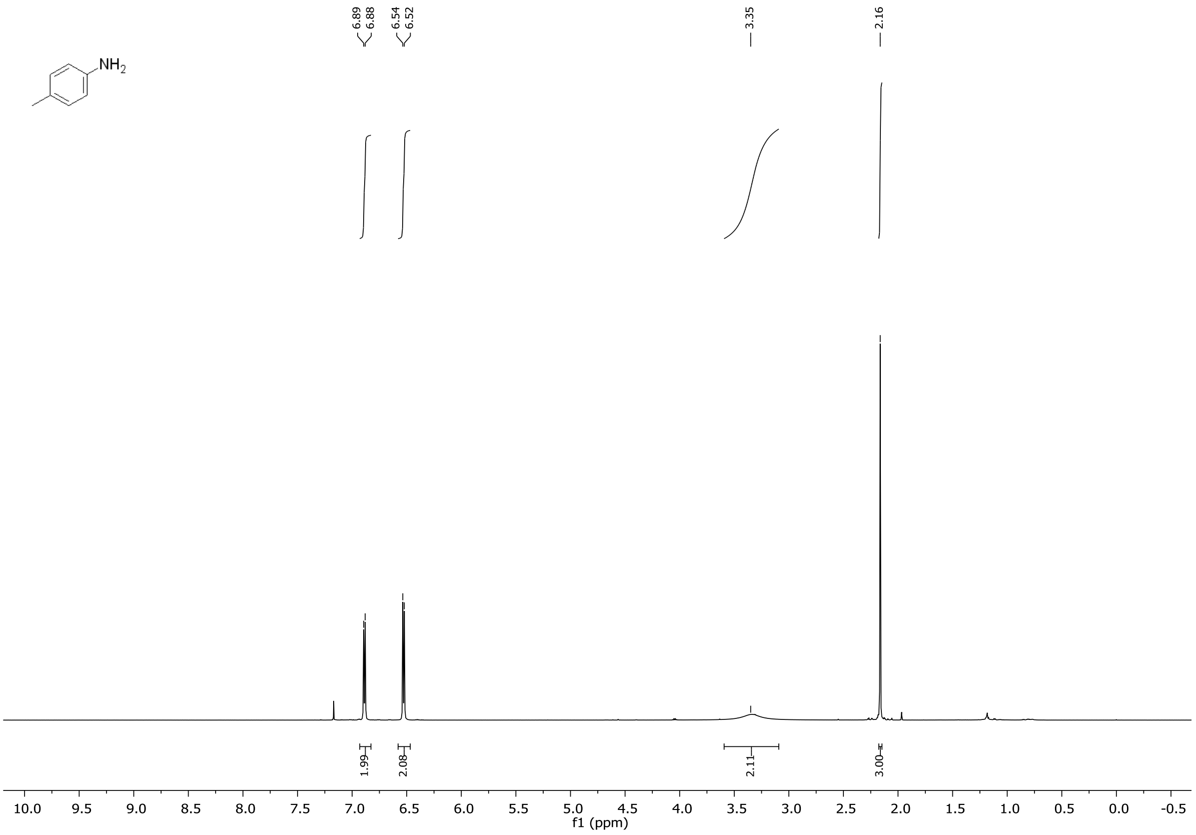

^{13}C NMR spectra of **2b** ($CDCl_3$, 151 MHz)

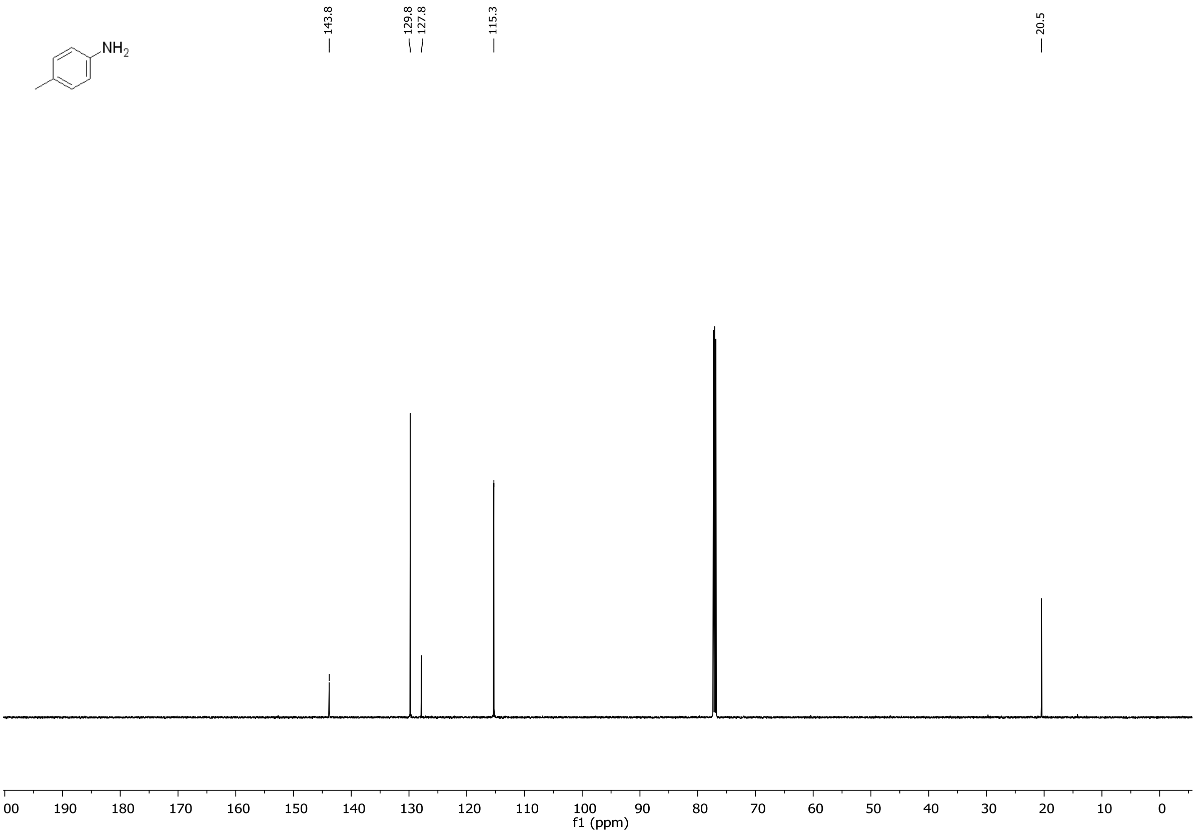

^{1}H NMR spectra of **2c** ($CDCl_3$, 600 MHz)

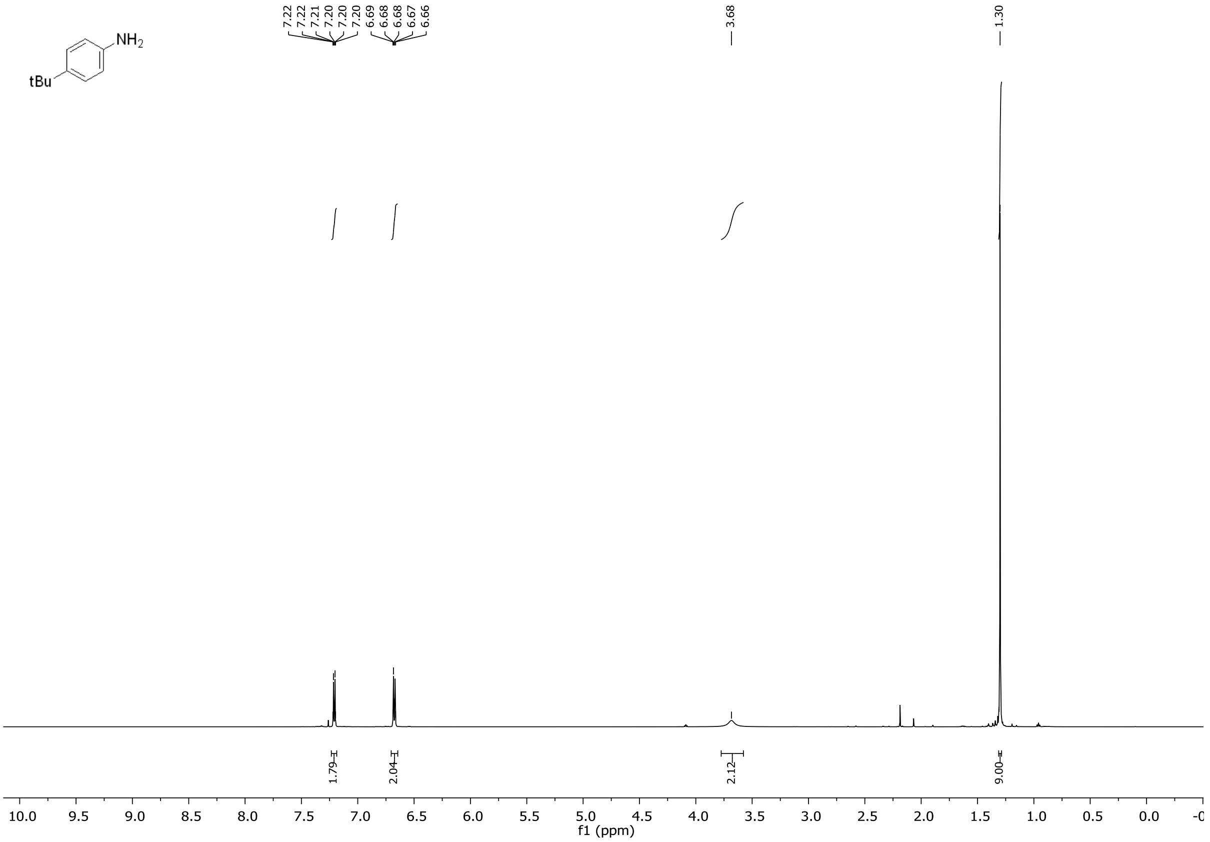

^{13}C NMR spectra of **2c** ($CDCl_3$, 151 MHz)

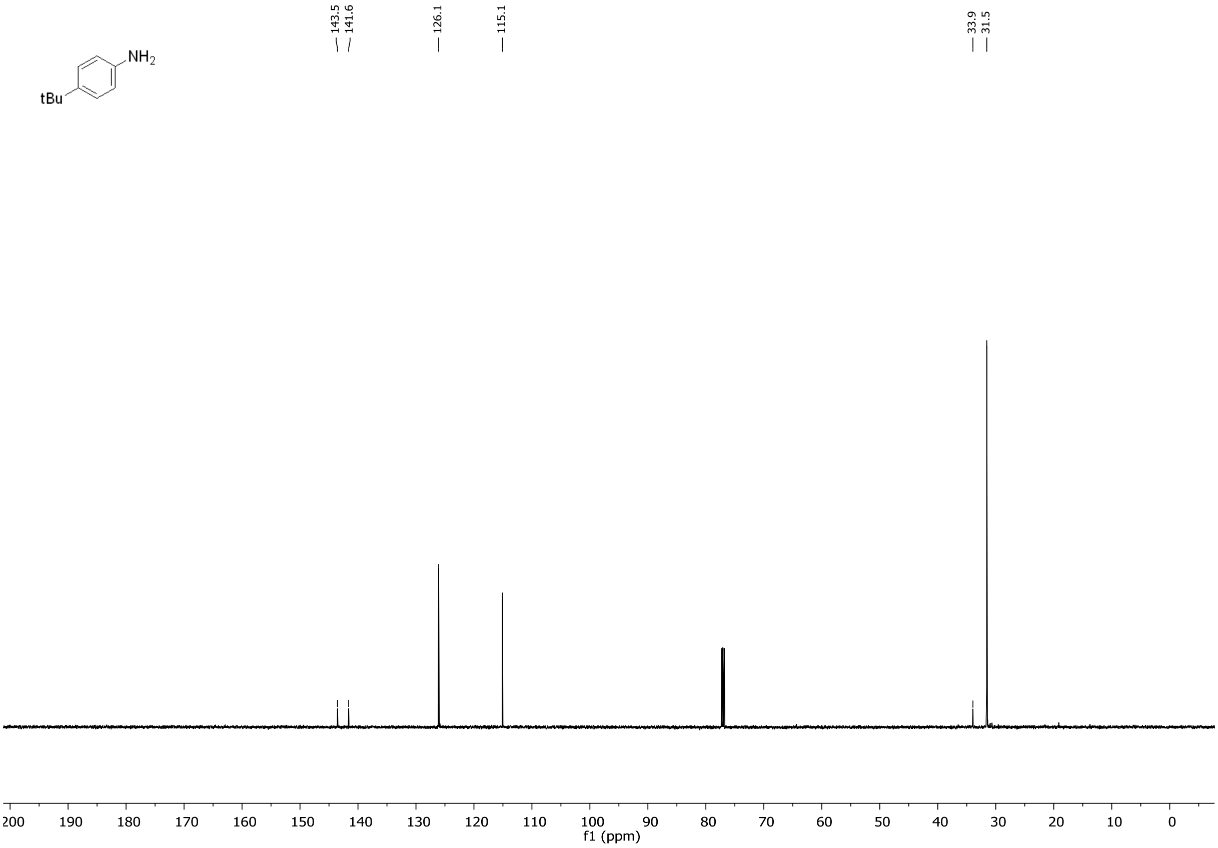

^{1}H NMR spectra of **2d** ($CDCl_3$, 600 MHz)

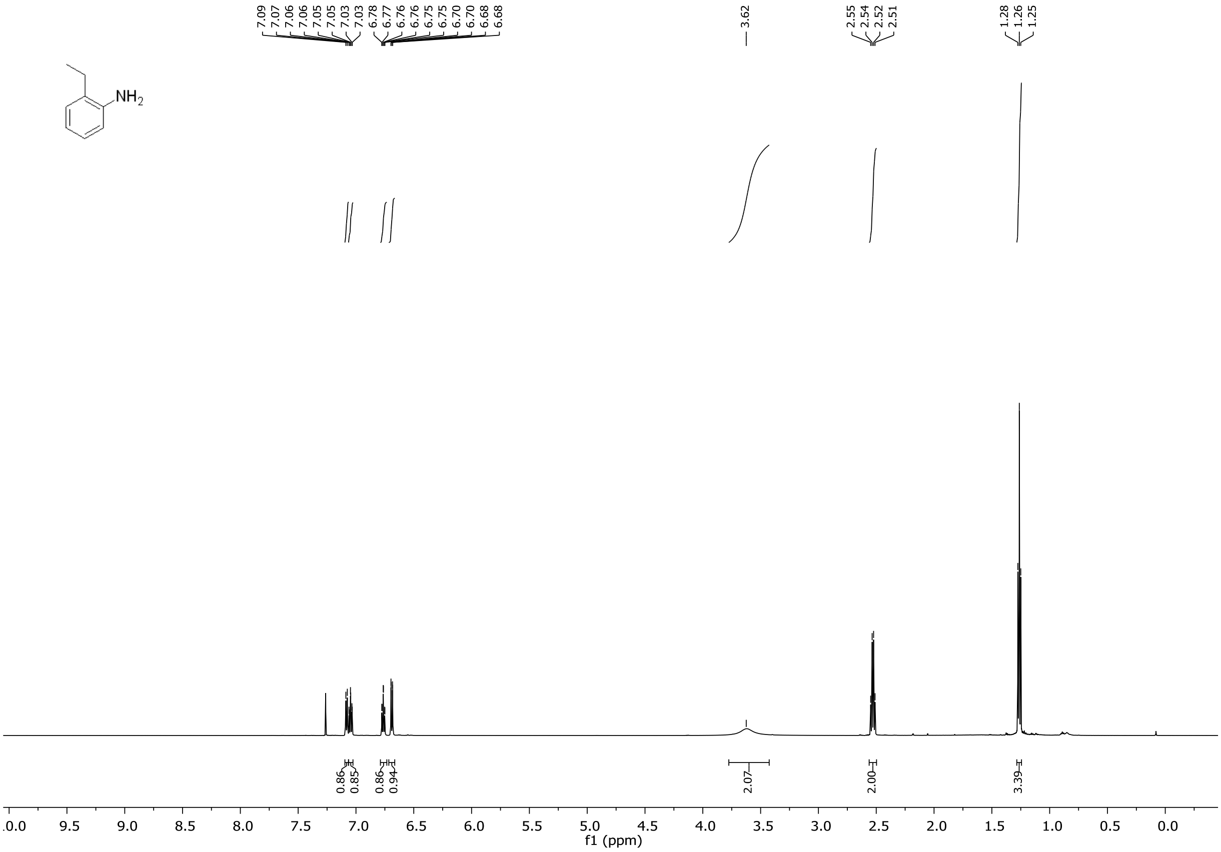

^{13}C NMR spectra of **2d** ($CDCl_3$, 151 MHz)

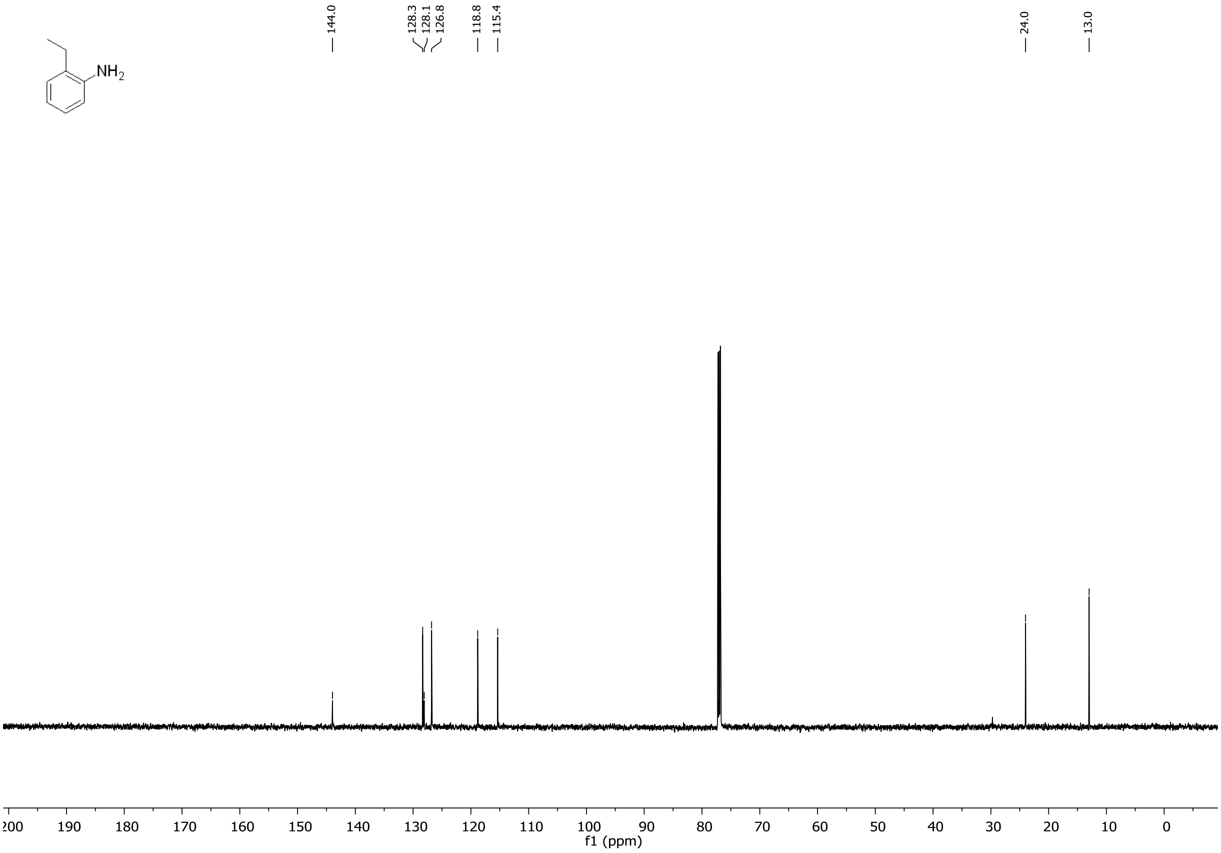

^{1}H NMR spectra of **2e** ($CDCl_3$, 400 MHz)

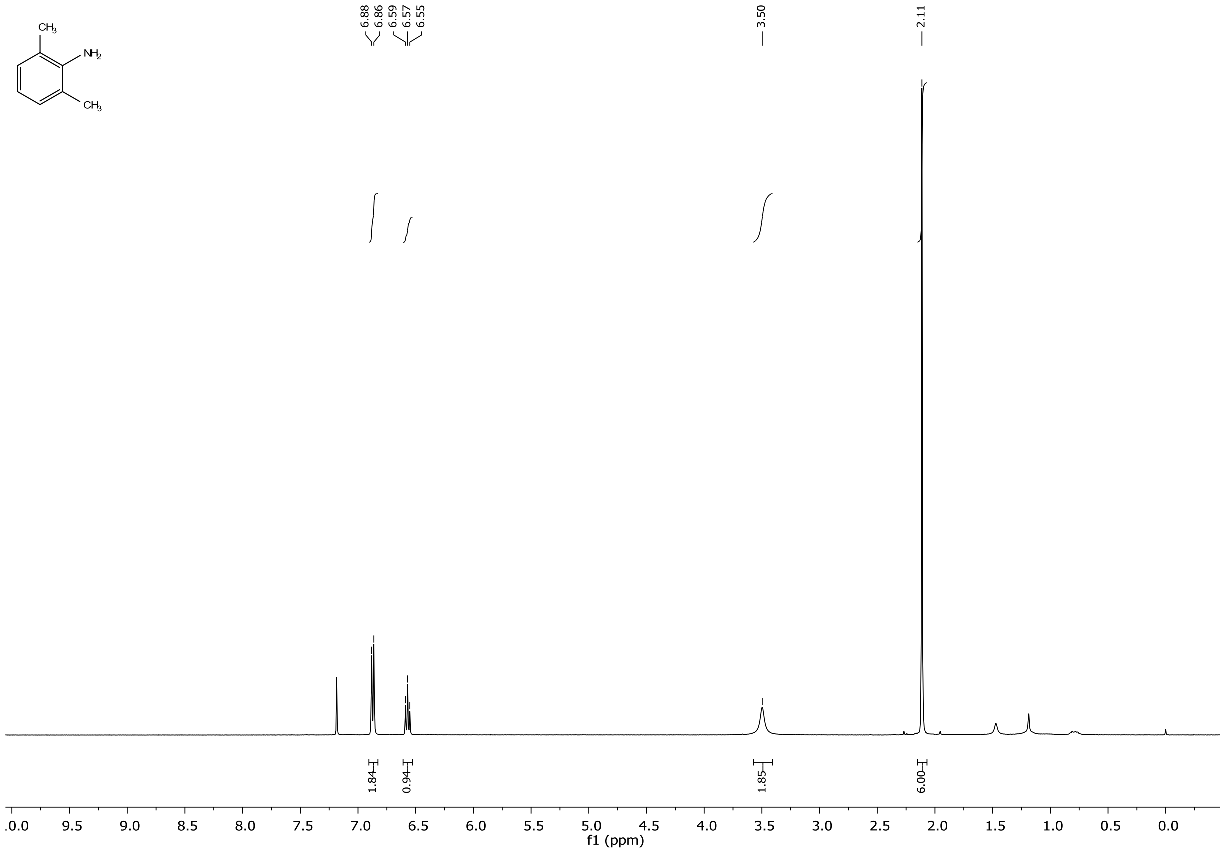

^{13}C NMR spectra of **2e** ($CDCl_3$, 101 MHz)

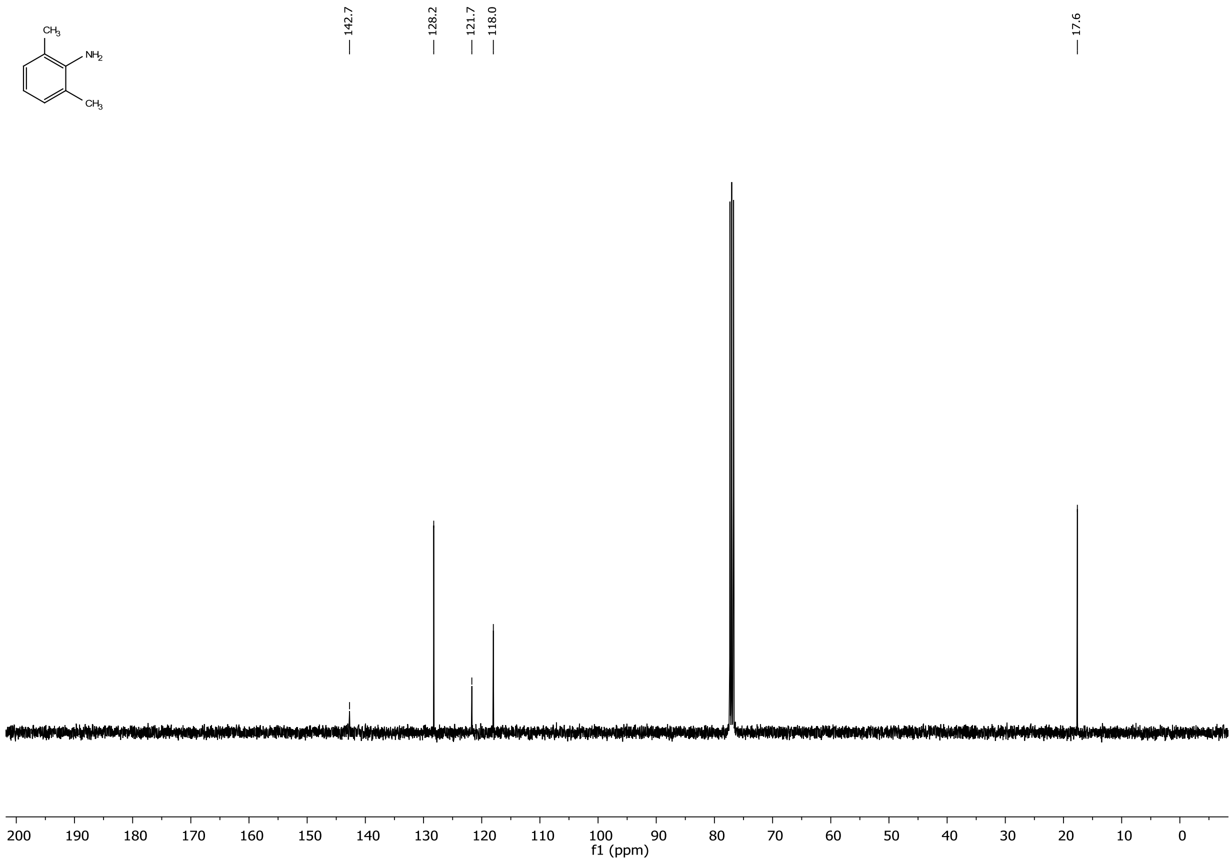

^{1}H NMR spectra of **2f** ($CDCl_3$, 600 MHz)

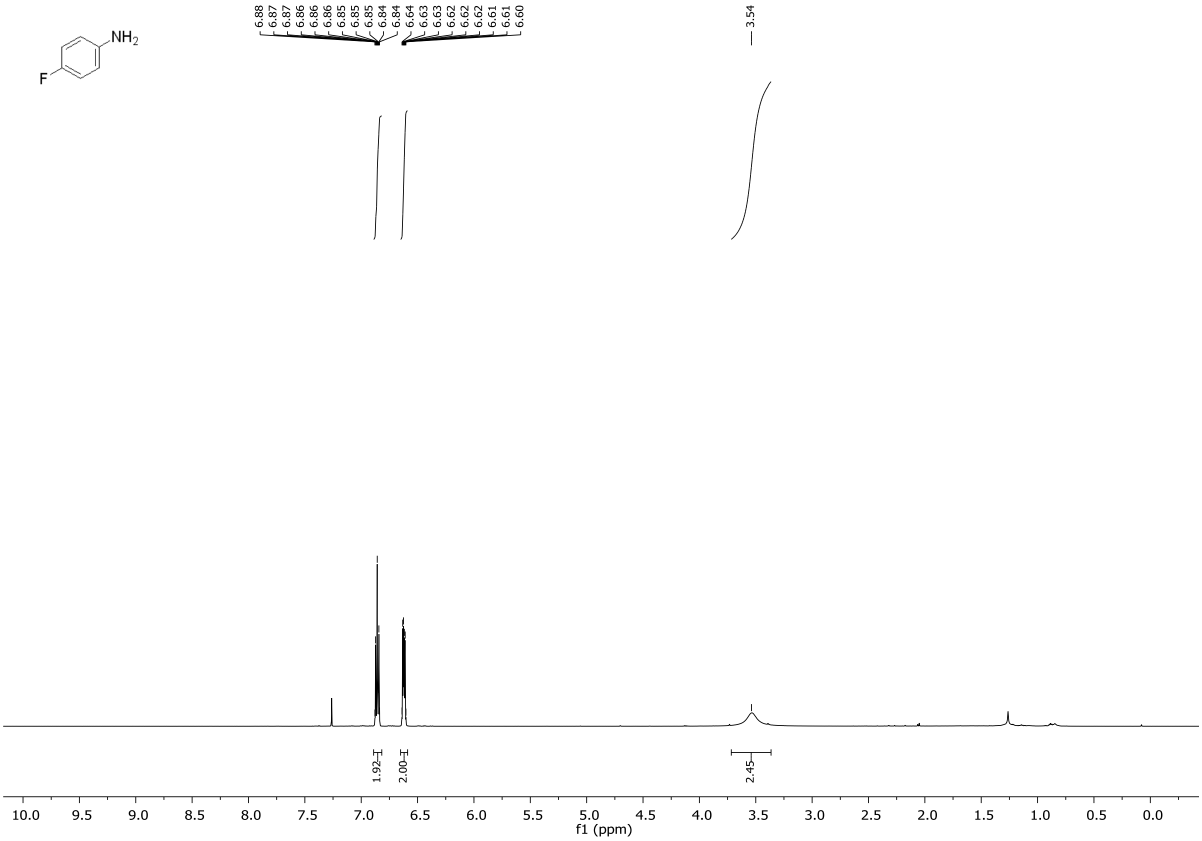

^{13}C NMR spectra of **2f** ($CDCl_3$, 151 MHz)

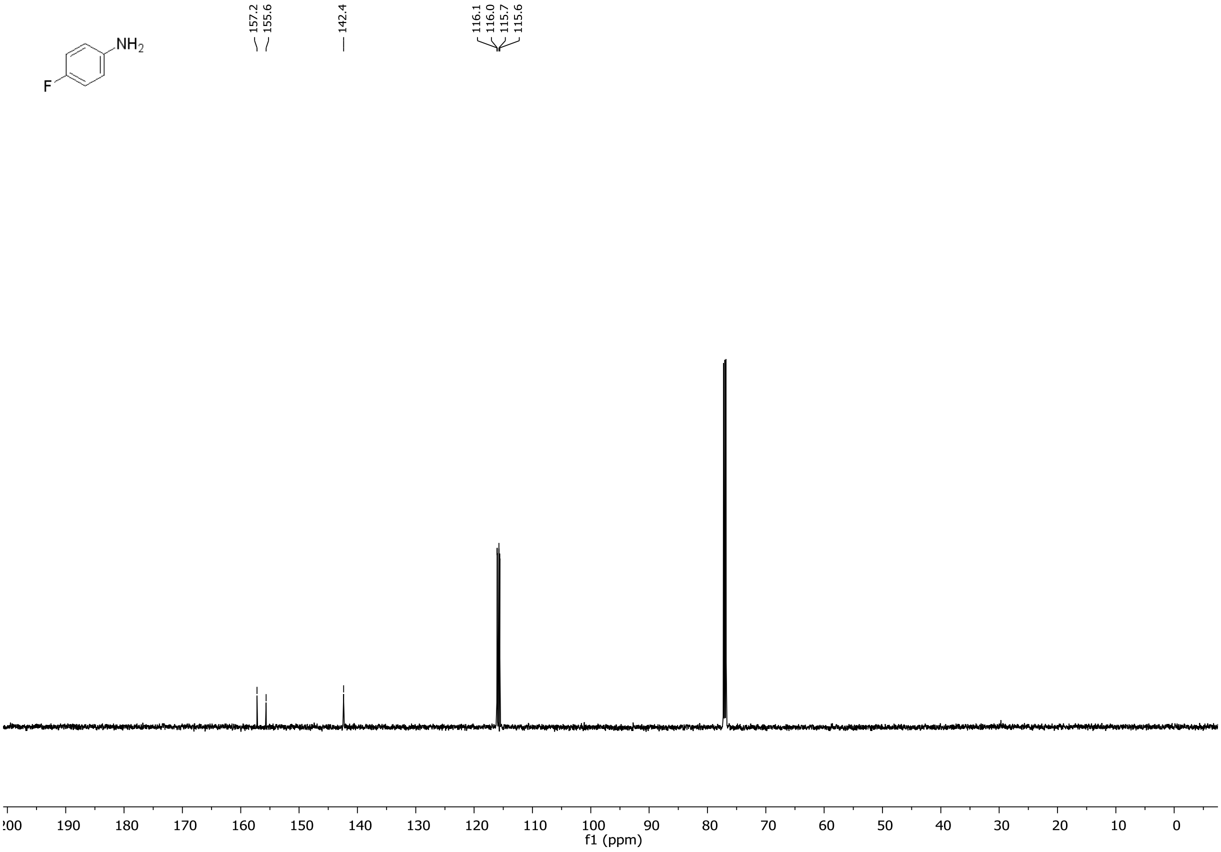

^{19}F NMR spectra of **2f** ($CDCl_3$, 564 MHz)

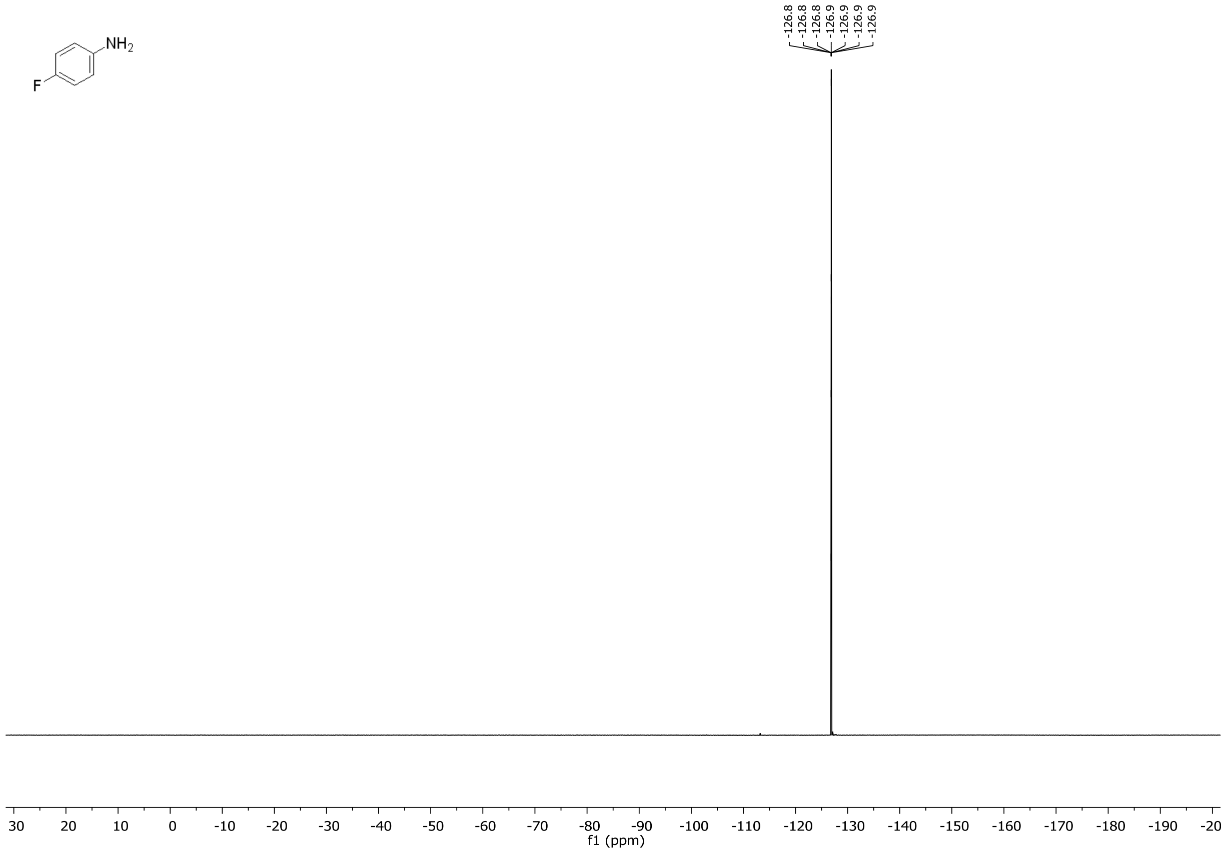

^{1}H NMR spectra of **2g** ($CDCl_3$, 600 MHz)

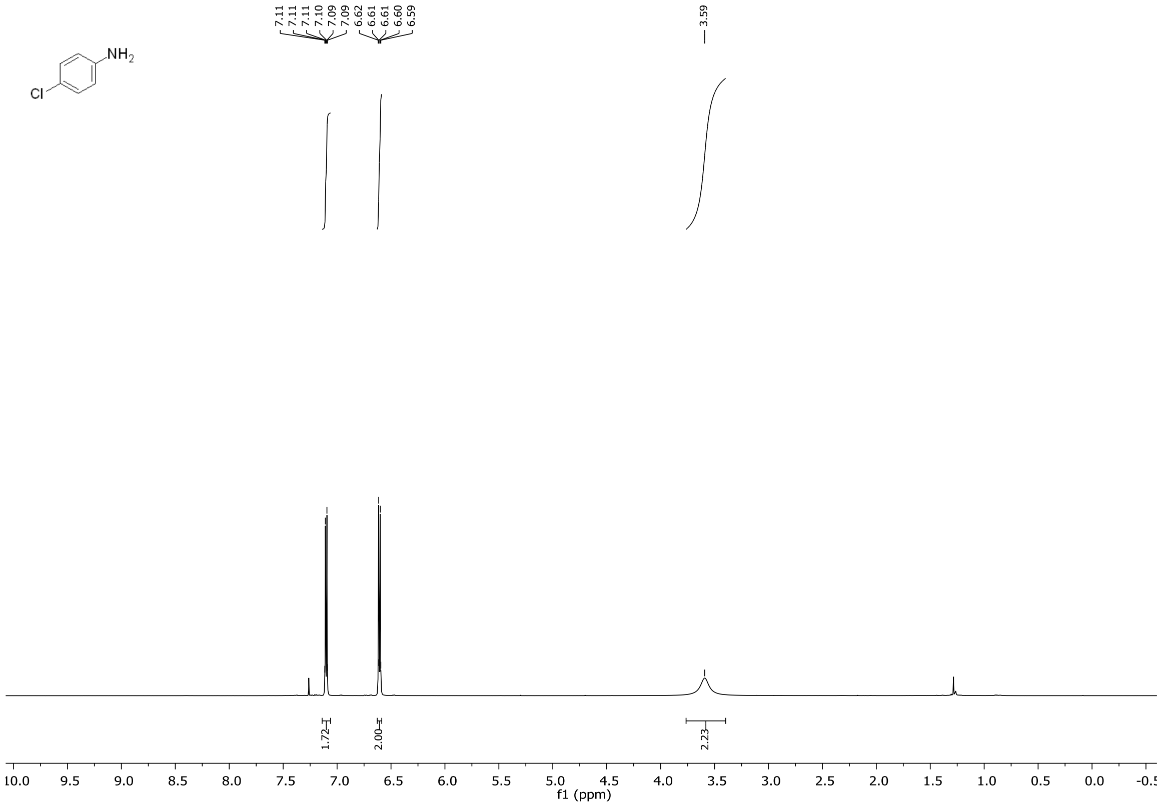

^{13}C NMR spectra of **2g** ($CDCl_3$, 151 MHz)

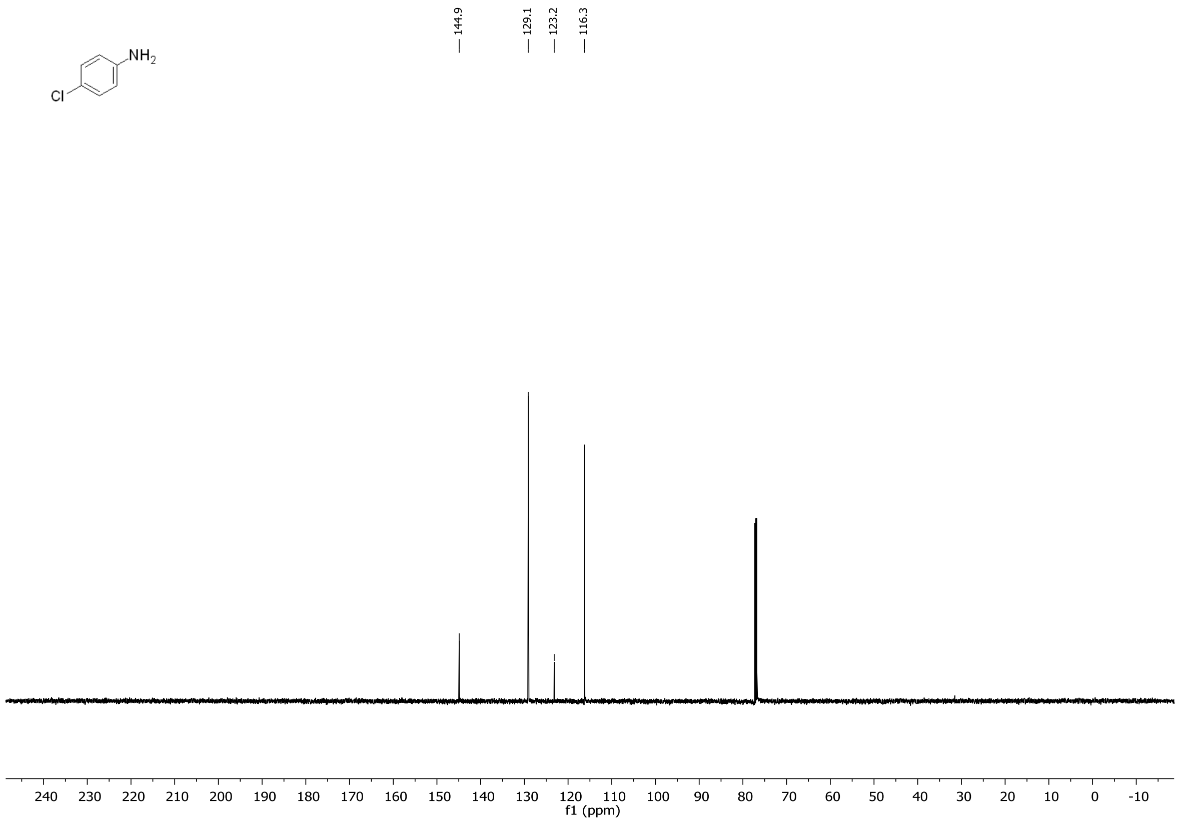

^{1}H NMR spectra of **2h** ($CDCl_3$, 600 MHz)

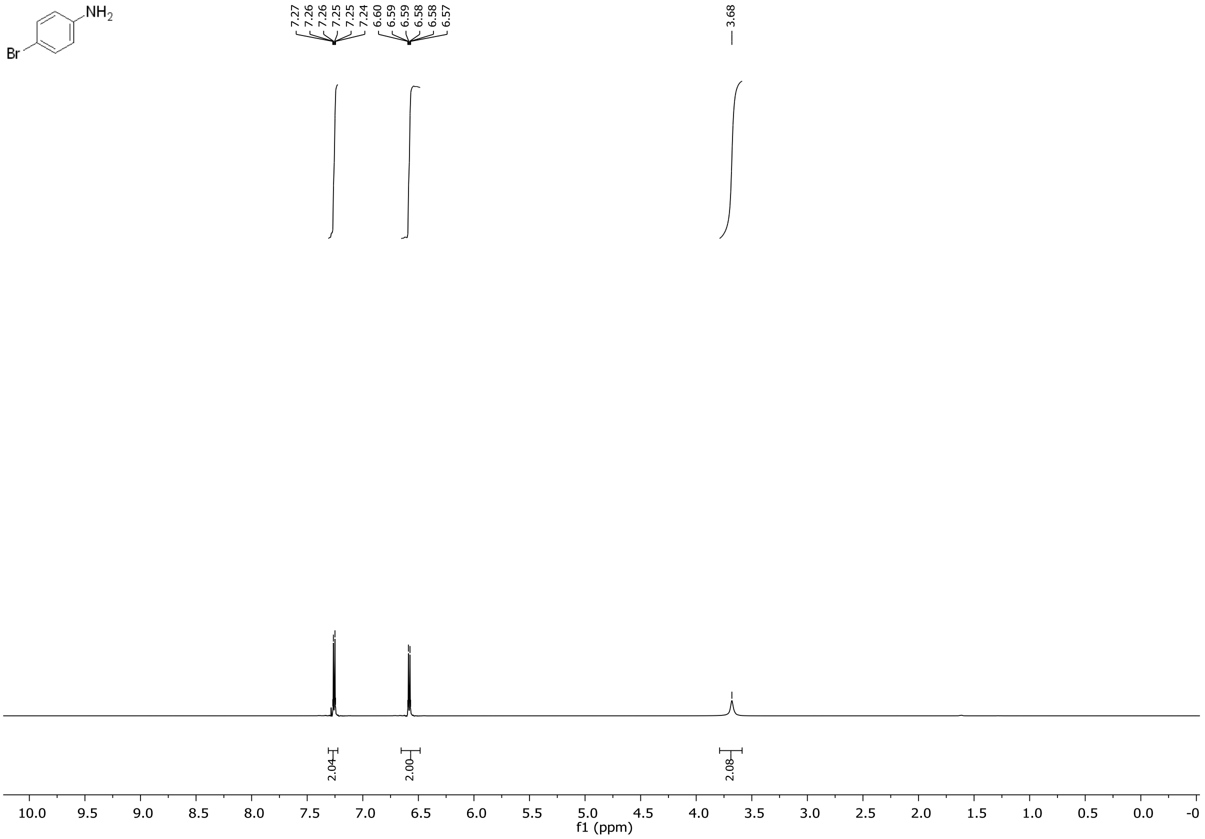

^{13}C NMR spectra of **2h** ($CDCl_3$, 151 MHz)

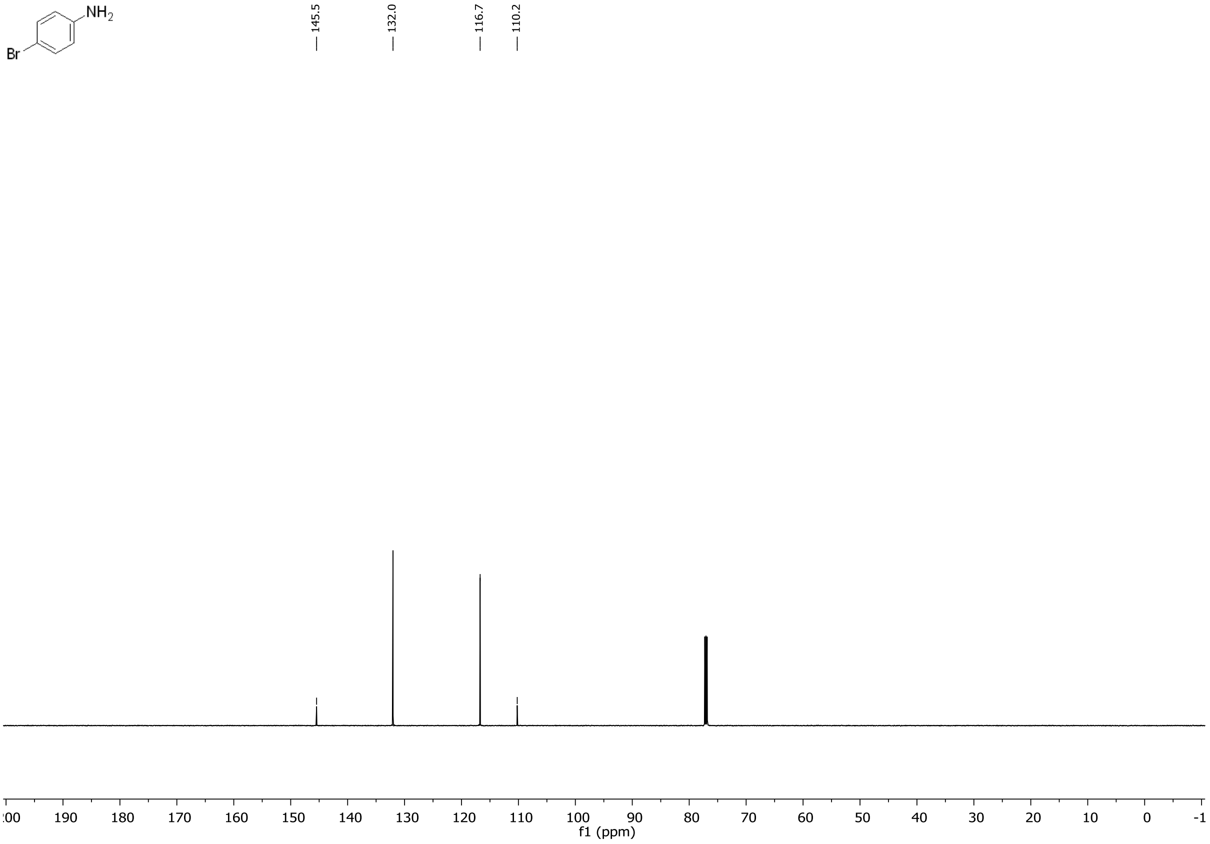

^{1}H NMR spectra of **2i** ($CDCl_3$, 600 MHz)

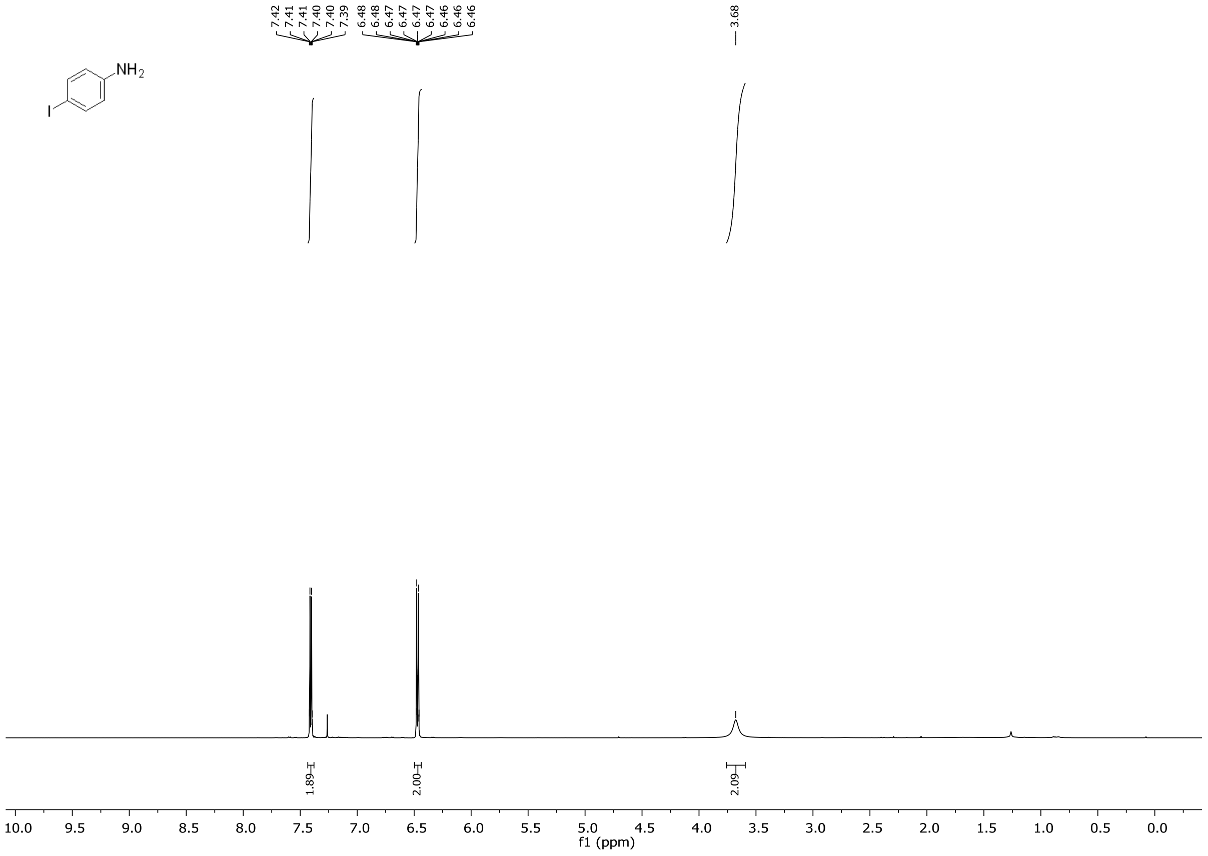

^{13}C NMR spectra of **2i** ($CDCl_3$, 151 MHz)

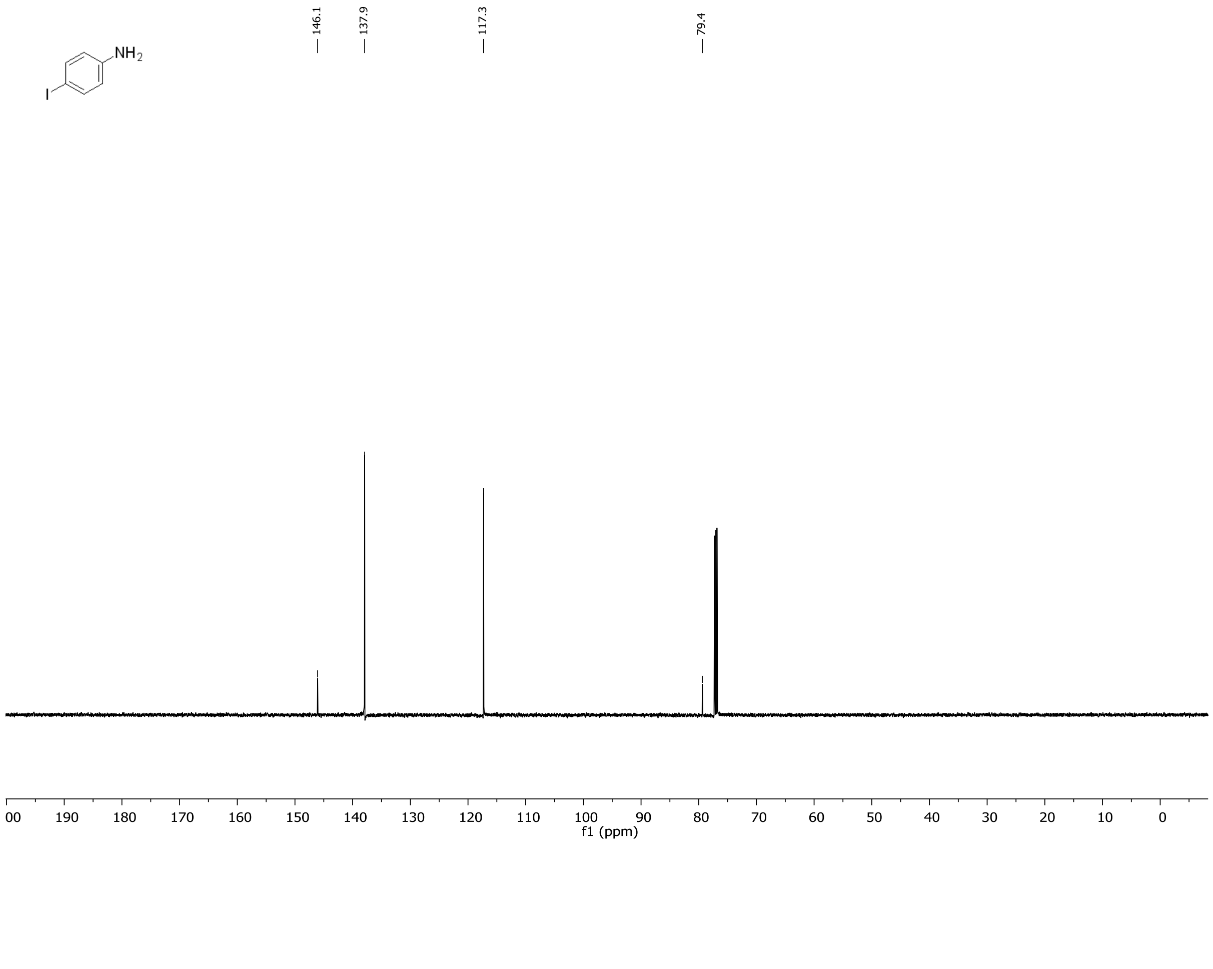

^{1}H NMR spectra of **2j** ($CDCl_3$, 600 MHz)

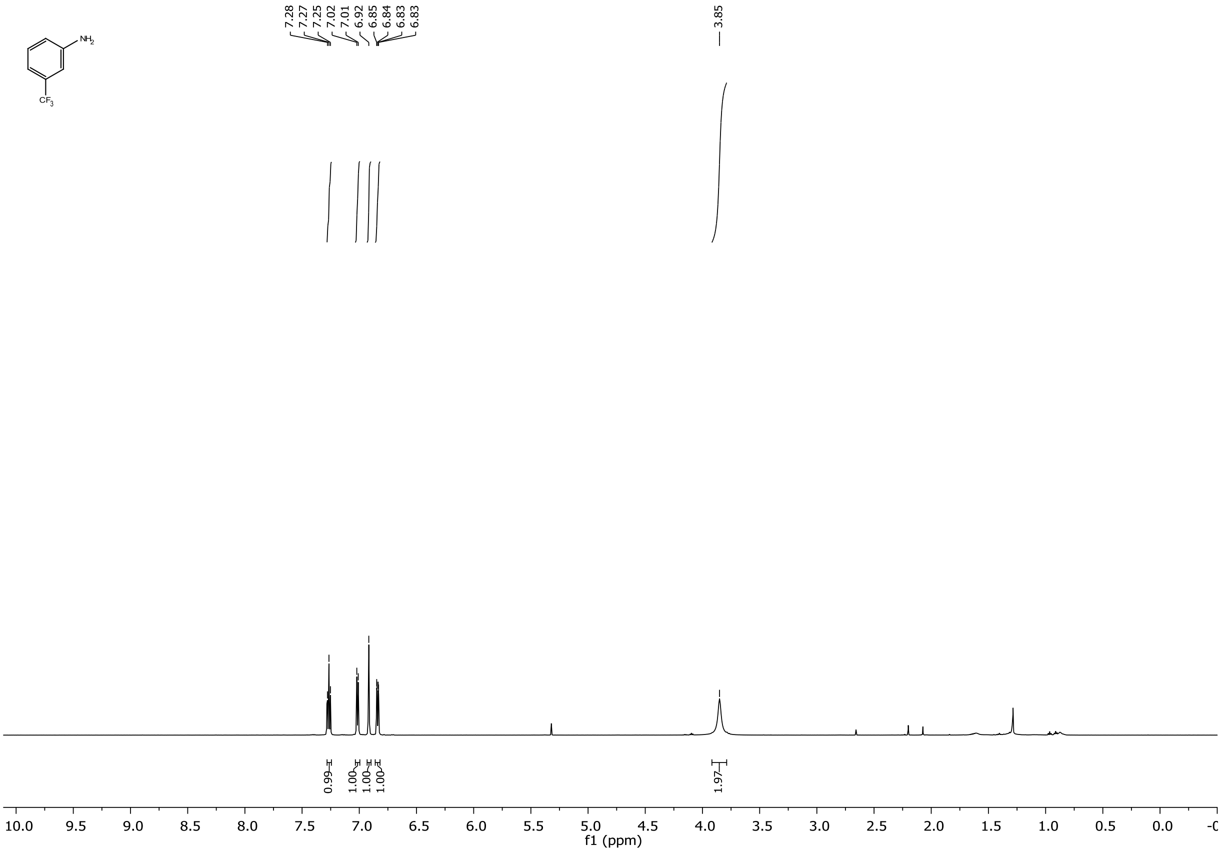

^{13}C NMR spectra of **2j** ($CDCl_3$, 151 MHz)

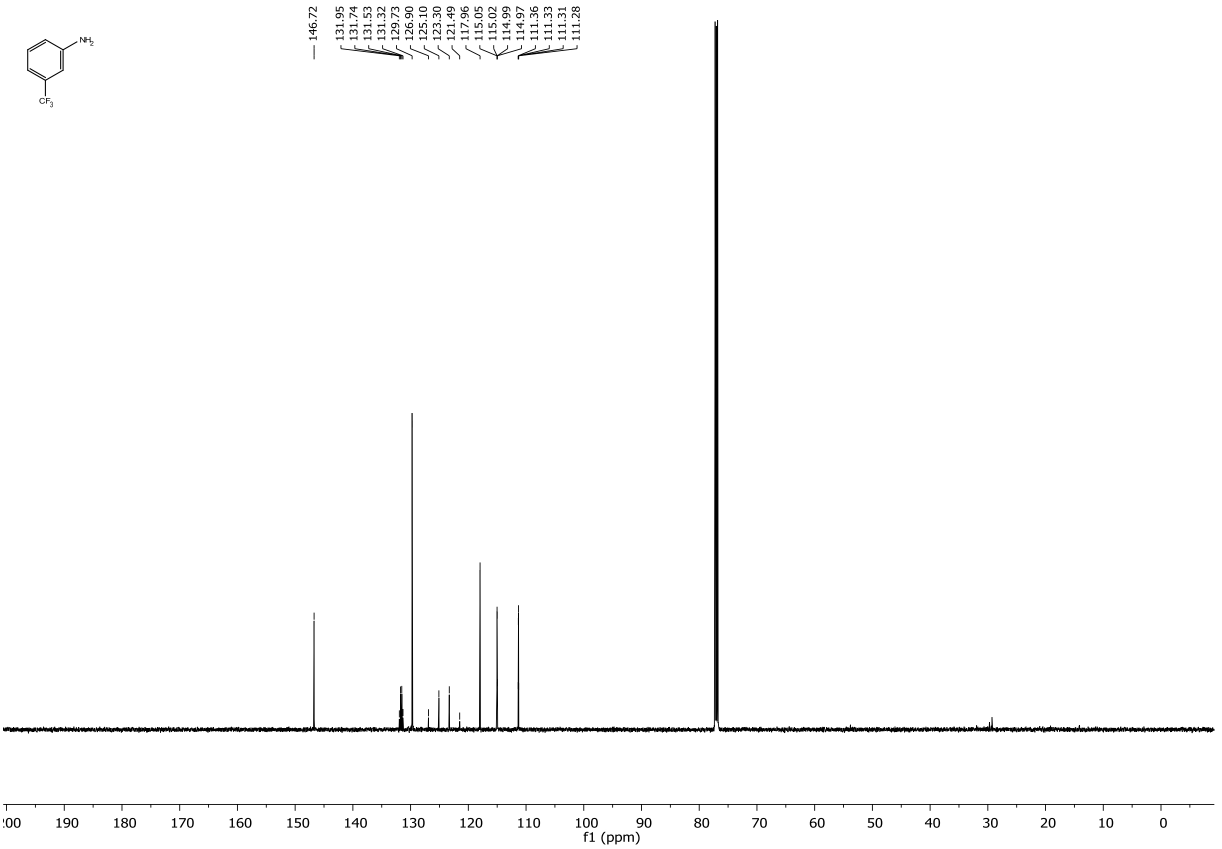

^{19}F NMR spectra of **2j** ($CDCl_3$, 565 MHz)

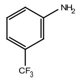

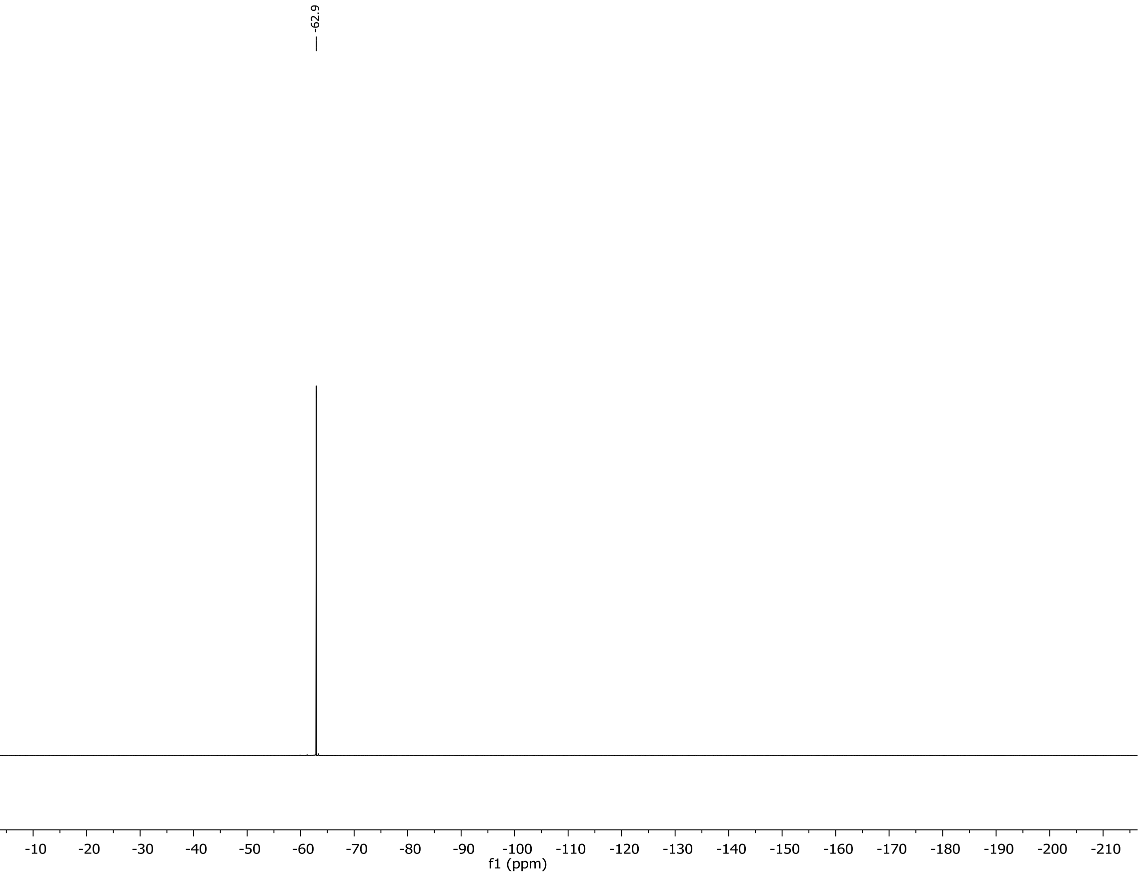

^{1}H NMR spectra of **2k** ($CDCl_3$, 400 MHz)

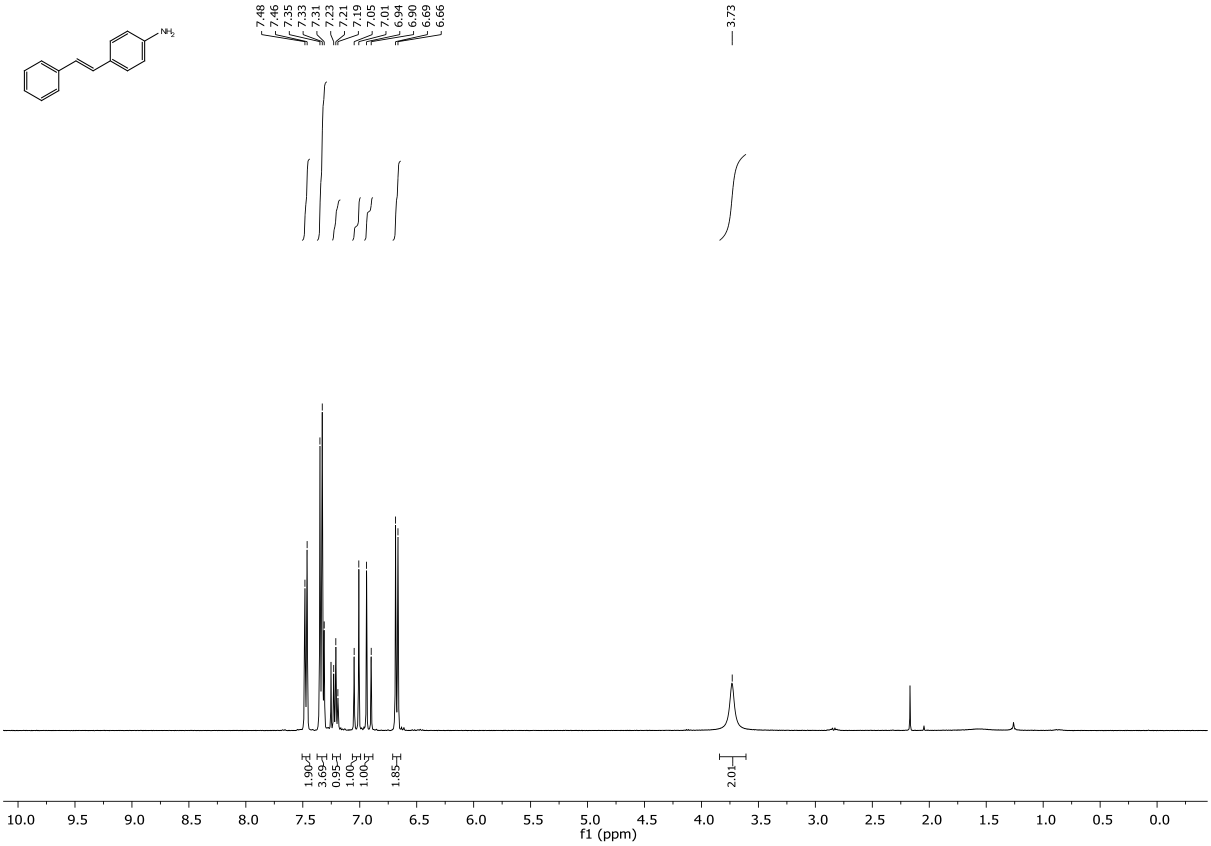

^{13}C NMR spectra of **2k** ($CDCl_3$, 101 MHz)

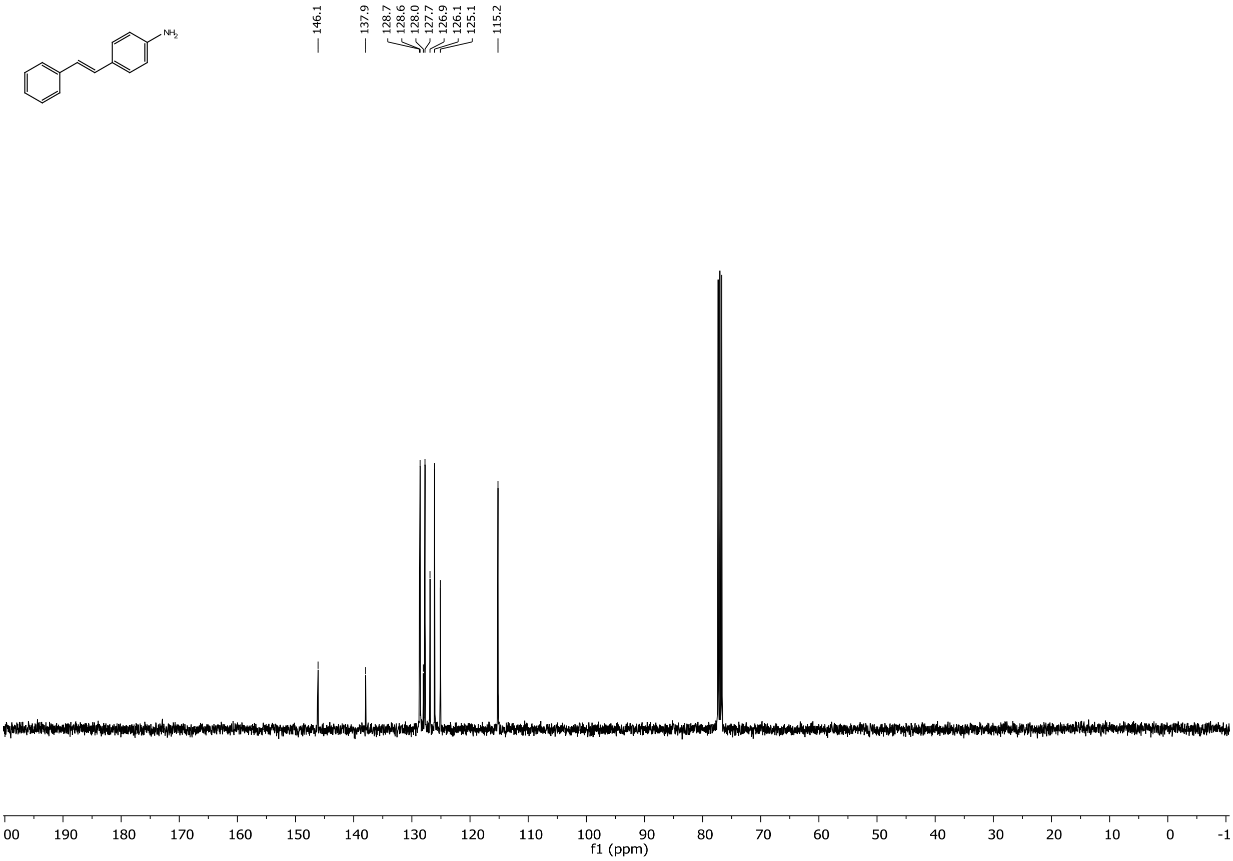

^{1}H NMR spectra of **2l** ($CDCl_3$, 600 MHz)

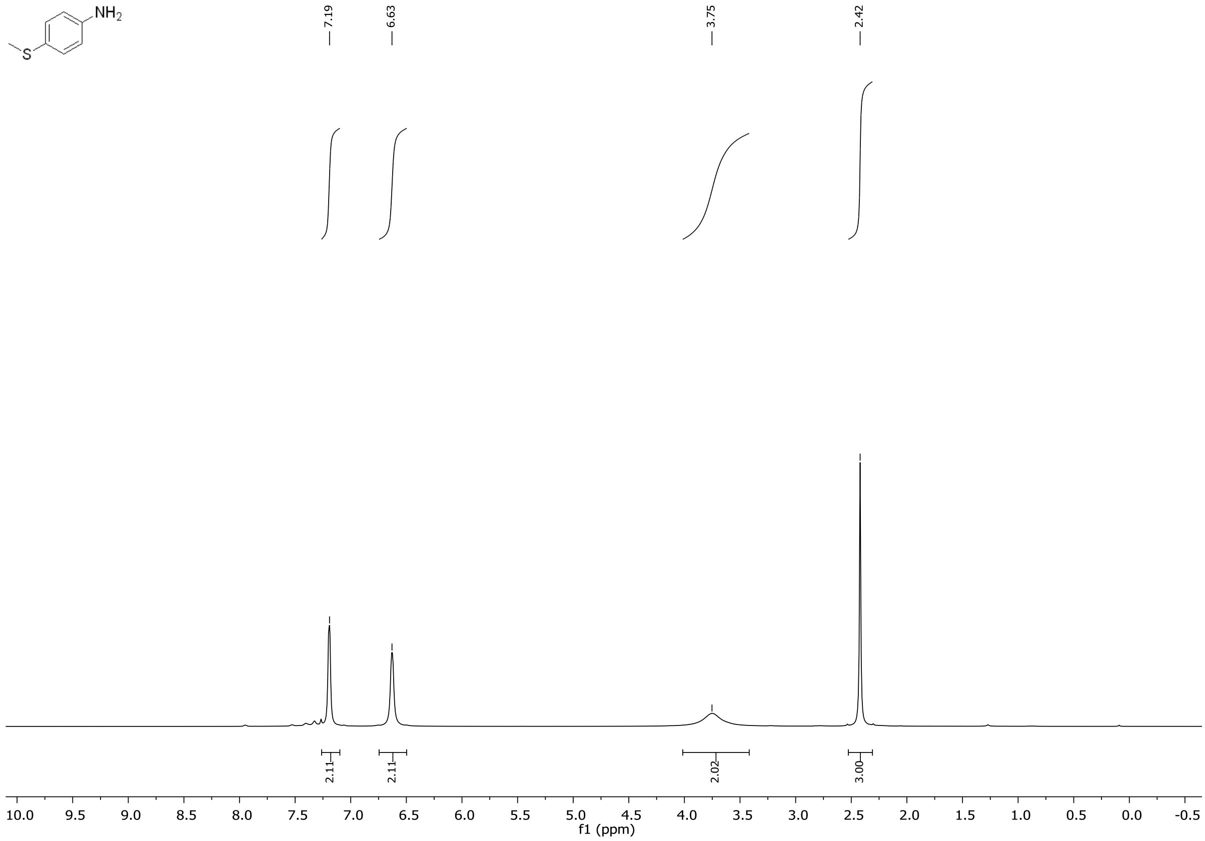

^{13}C NMR spectra of **2l** ($CDCl_3$, 151 MHz)

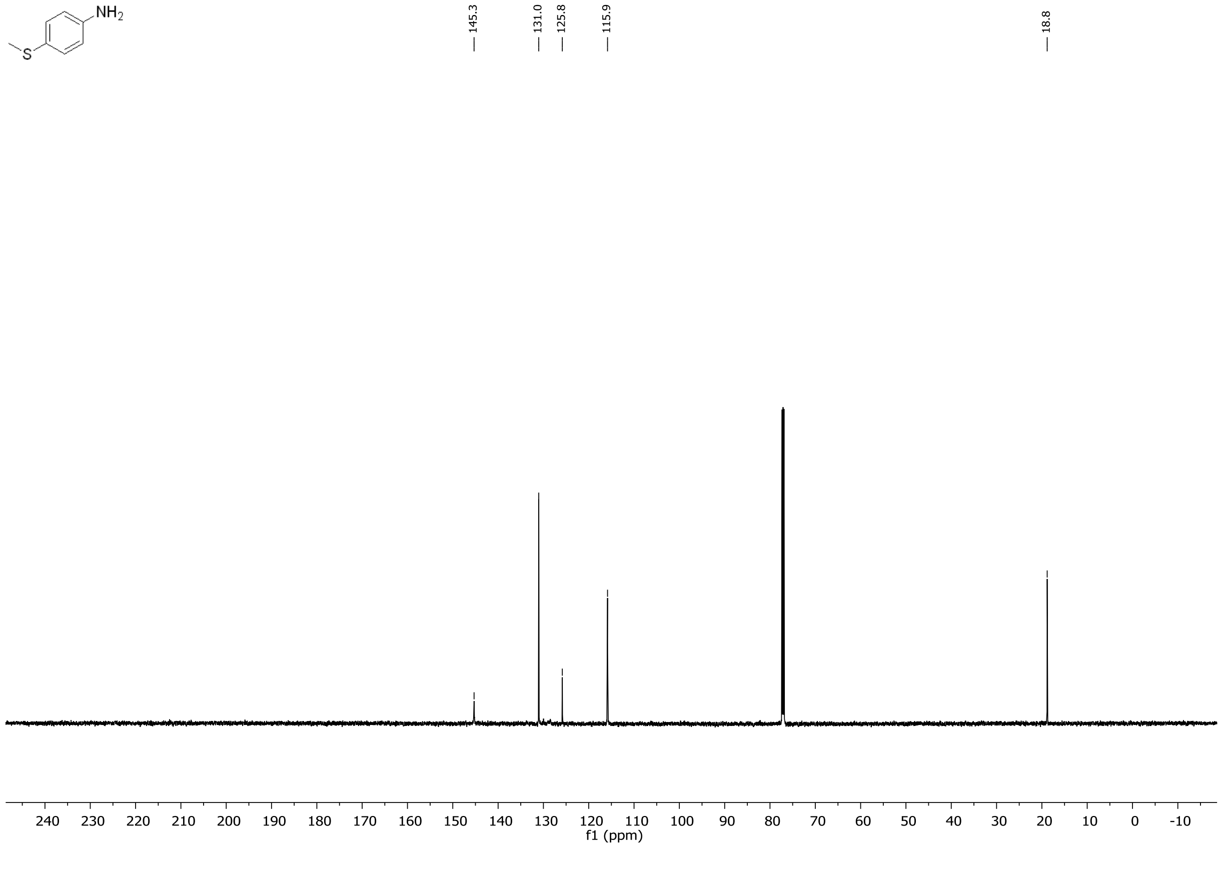

^{1}H NMR spectra of **2m** ($CDCl_3$, 600 MHz)

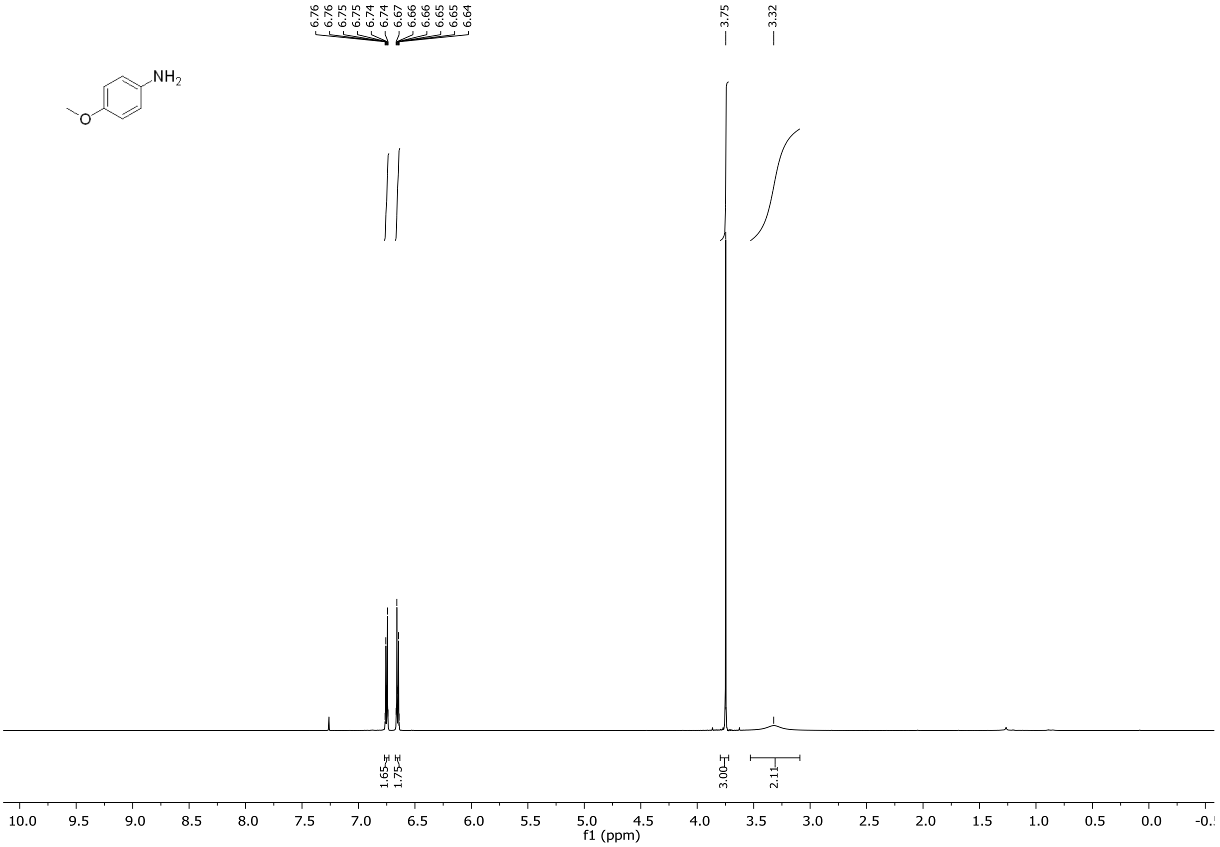

^{13}C NMR spectra of **2m** ($CDCl_3$, 151 MHz)

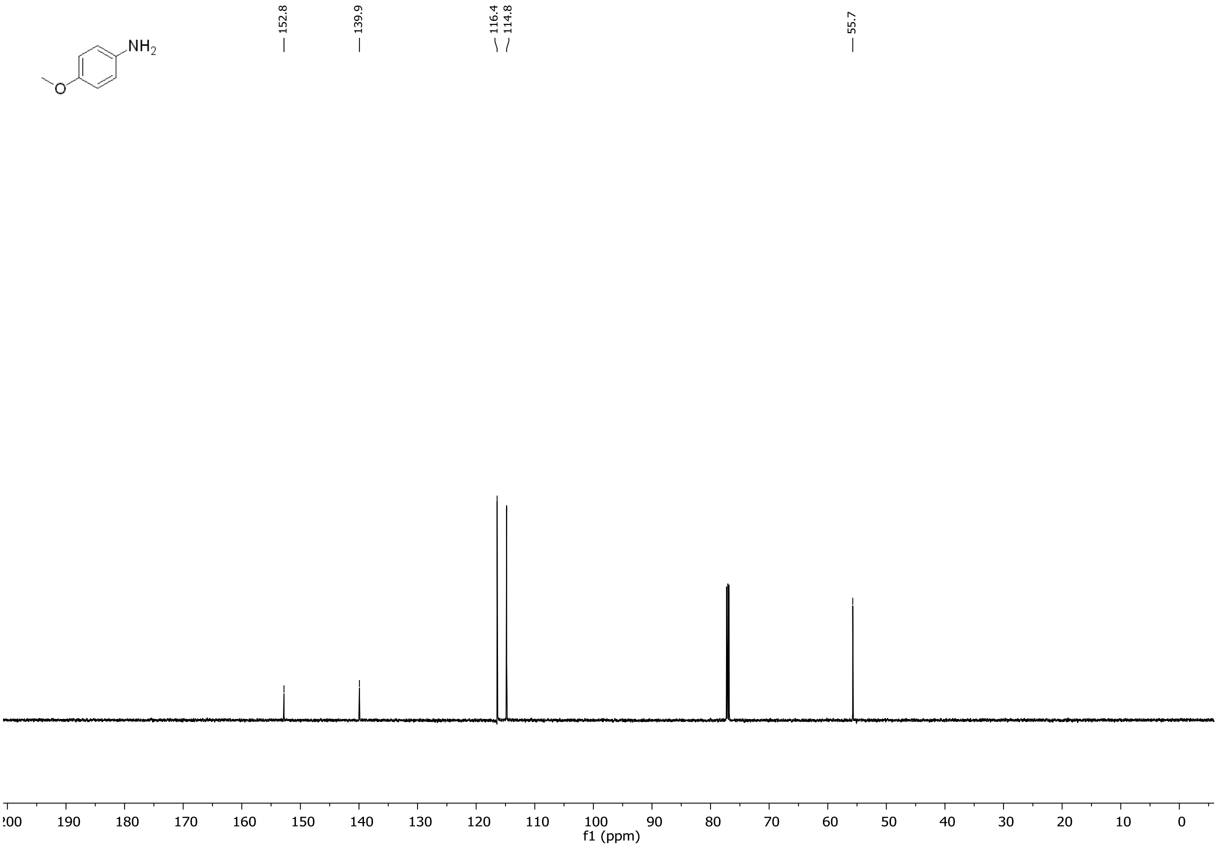

^{1}H NMR spectra of **2n** ($CDCl_3$, 400 MHz)

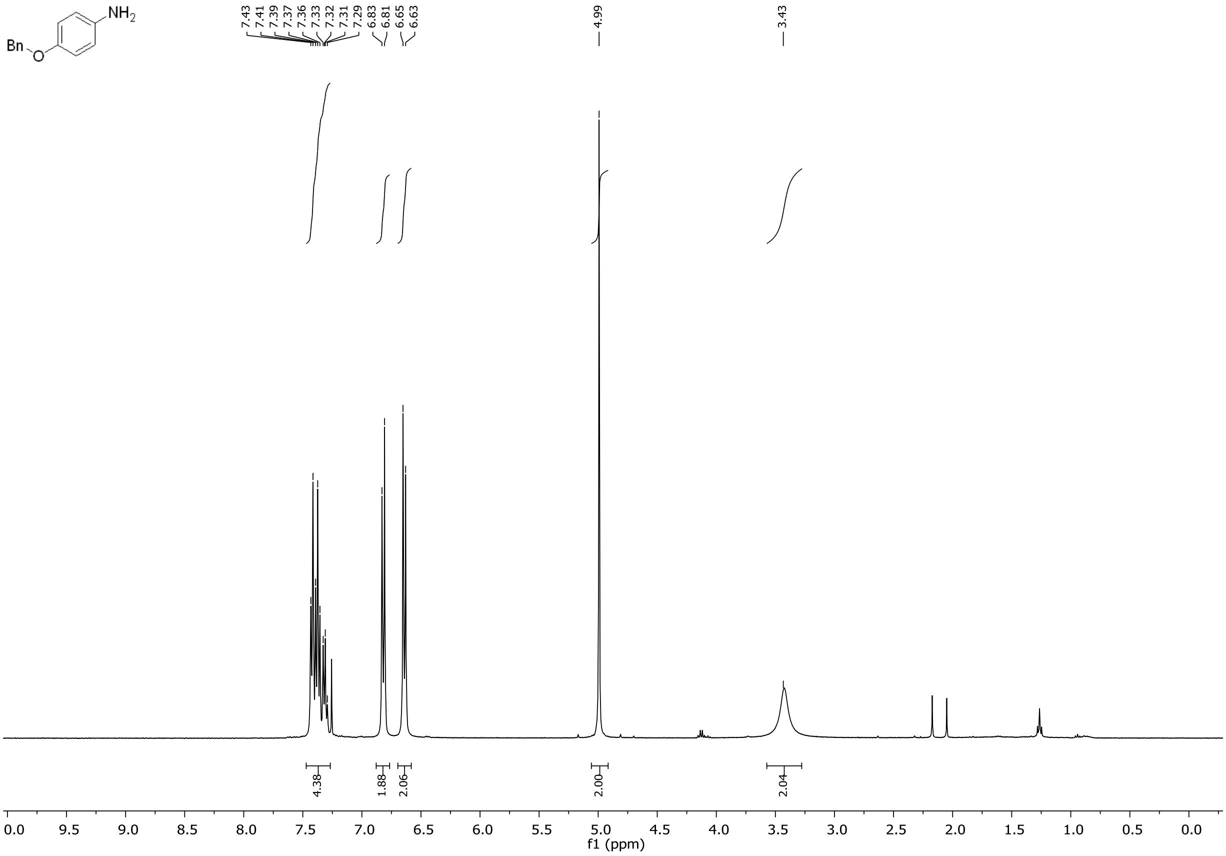

^{13}C NMR spectra of **2n** ($CDCl_3$, 101 MHz)

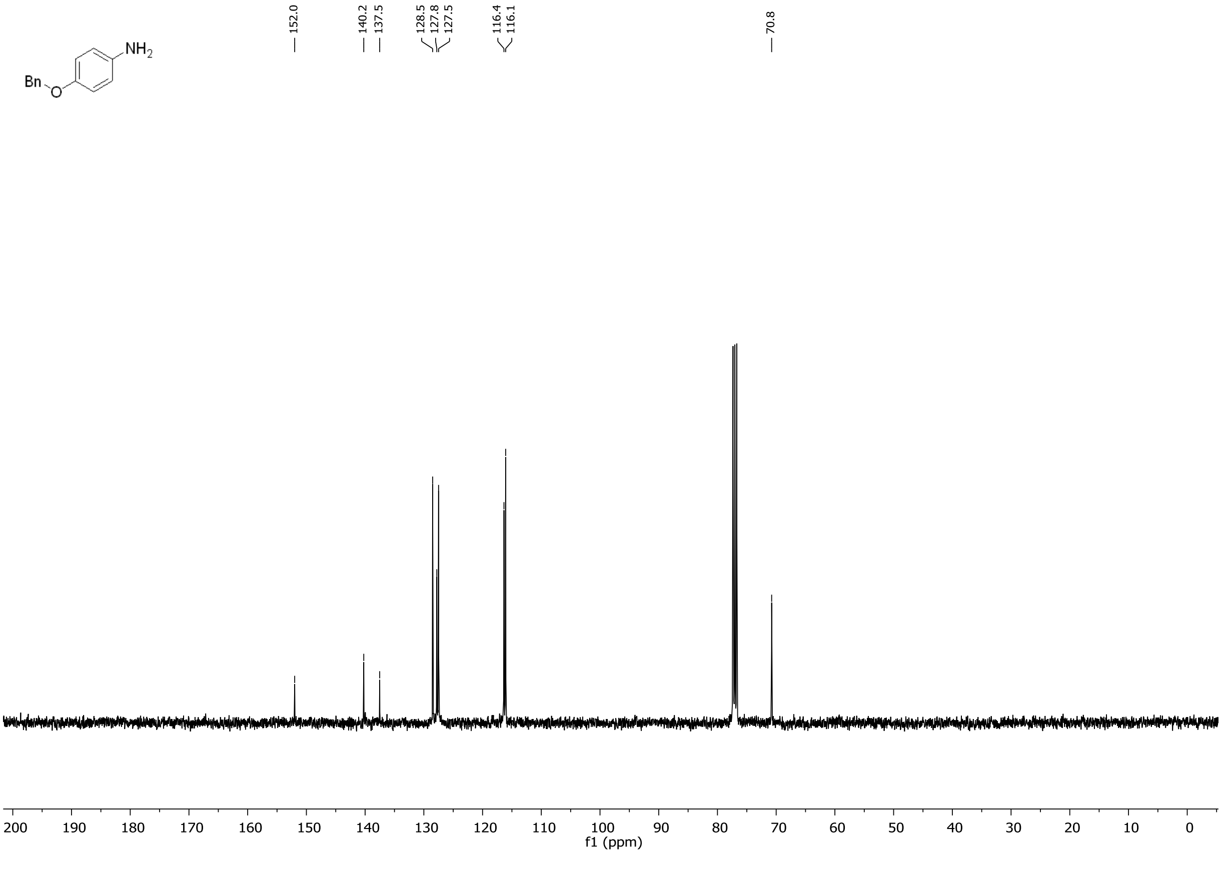

^{1}H NMR spectra of **2o** ($CDCl_3$, 600 MHz)

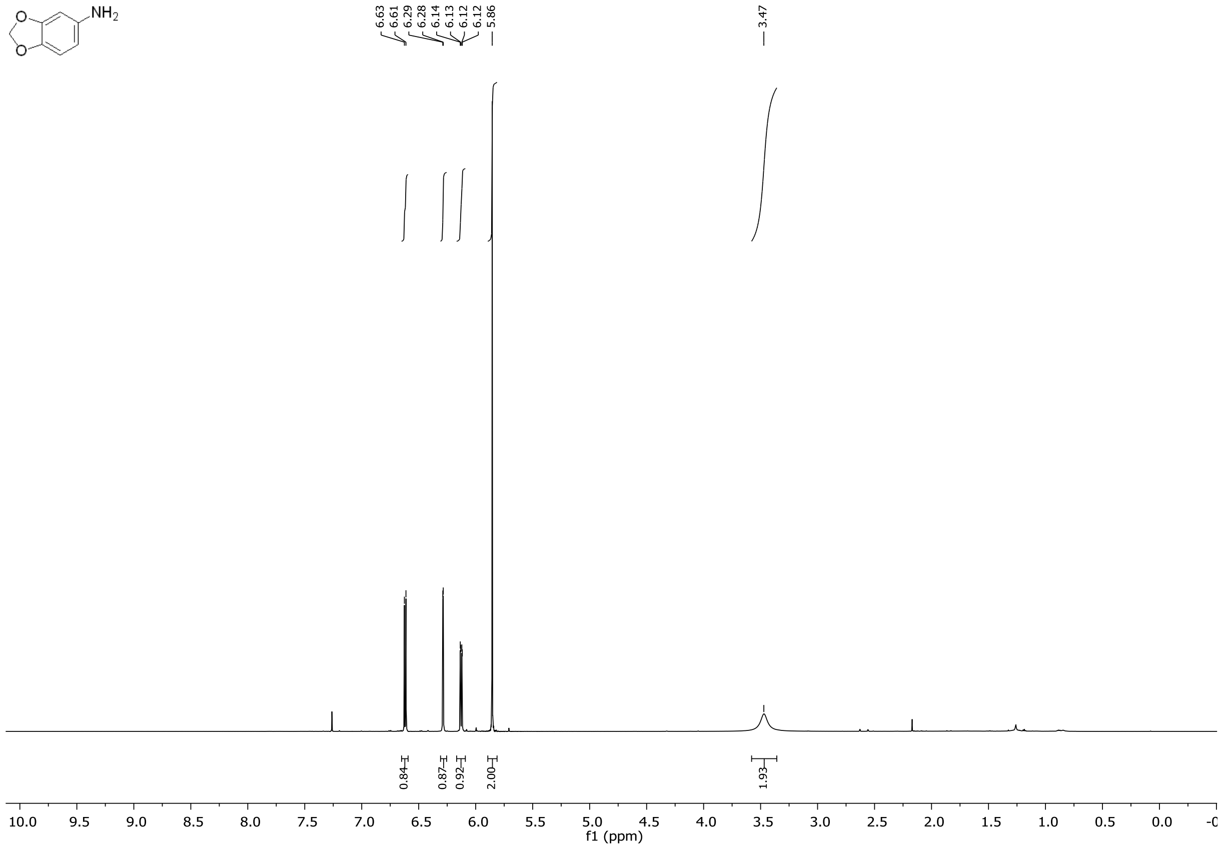

^{13}C NMR spectra of **2o** ($CDCl_3$, 151 MHz)

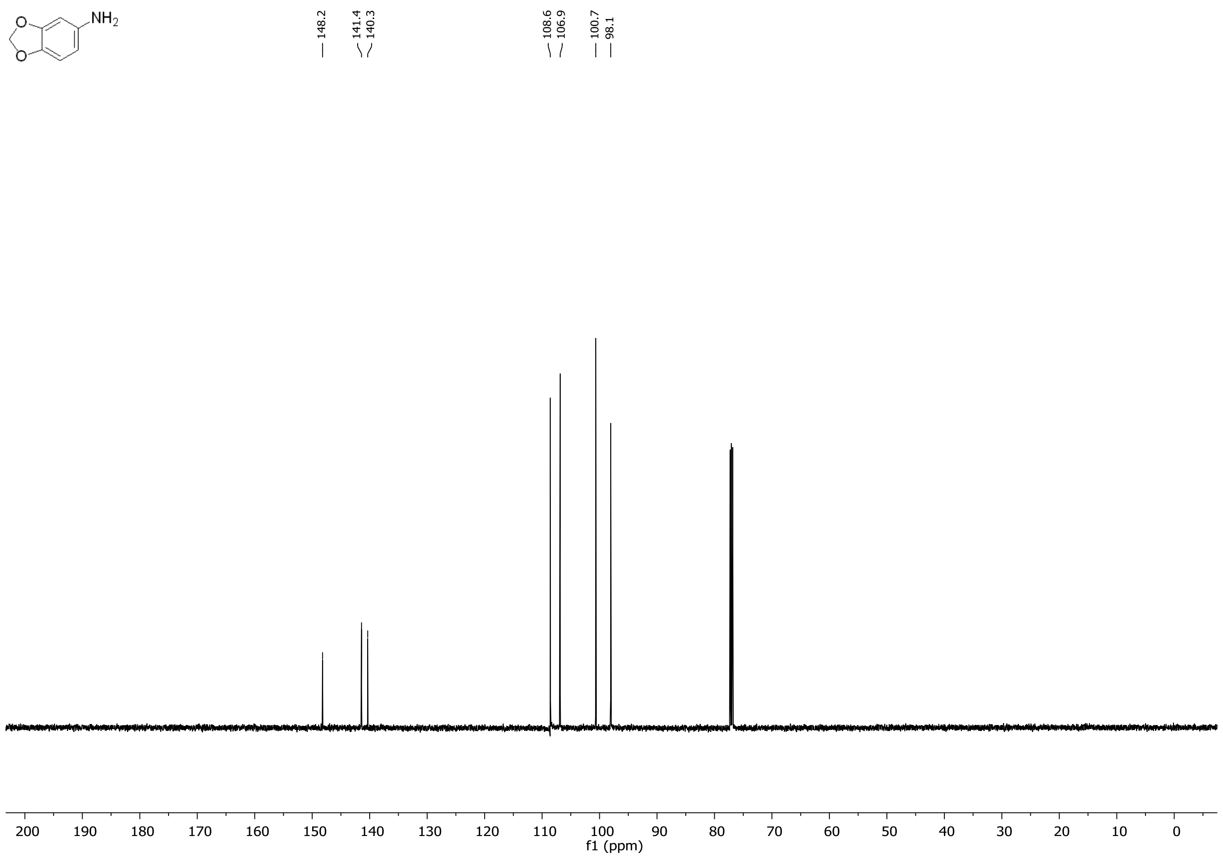

^{1}H NMR spectra of **2p** ($CDCl_3$, 400 MHz)

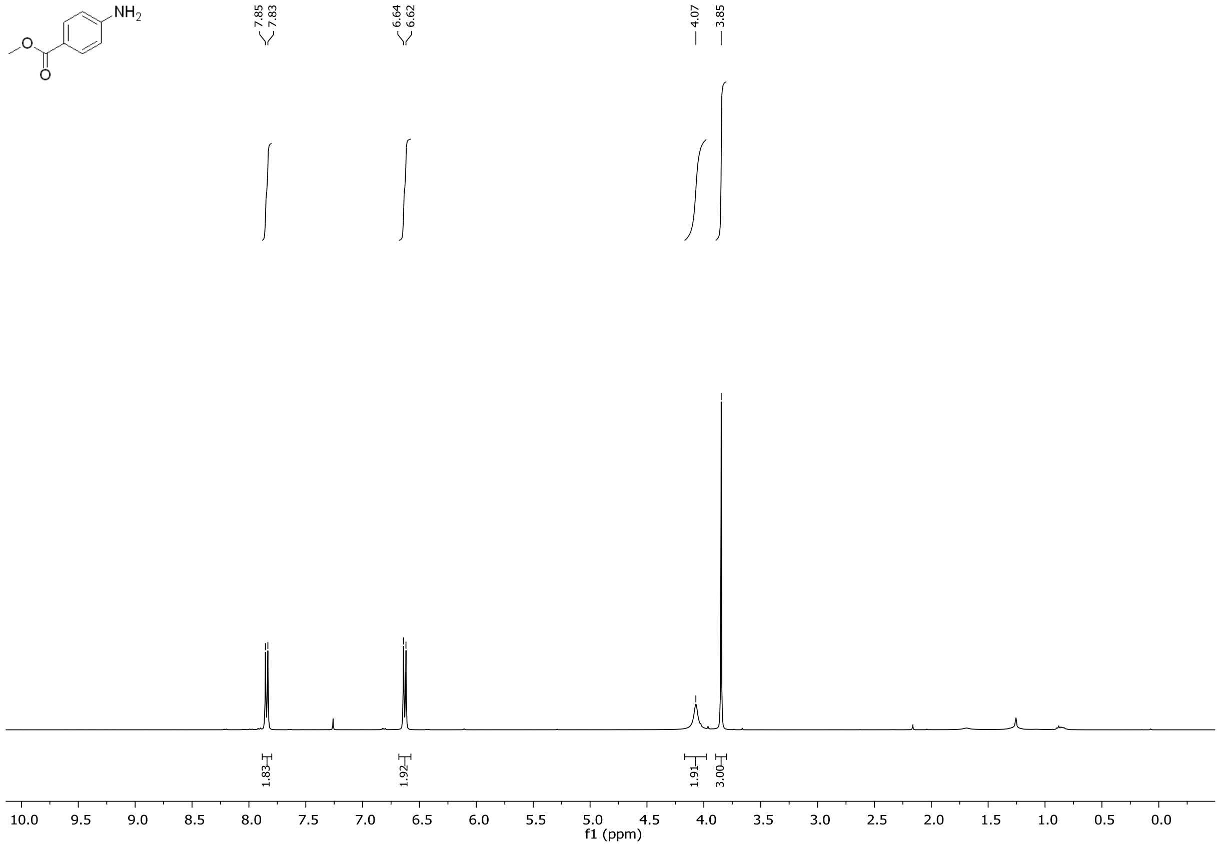

^{13}C NMR spectra of **2p** ($CDCl_3$, 101 MHz)

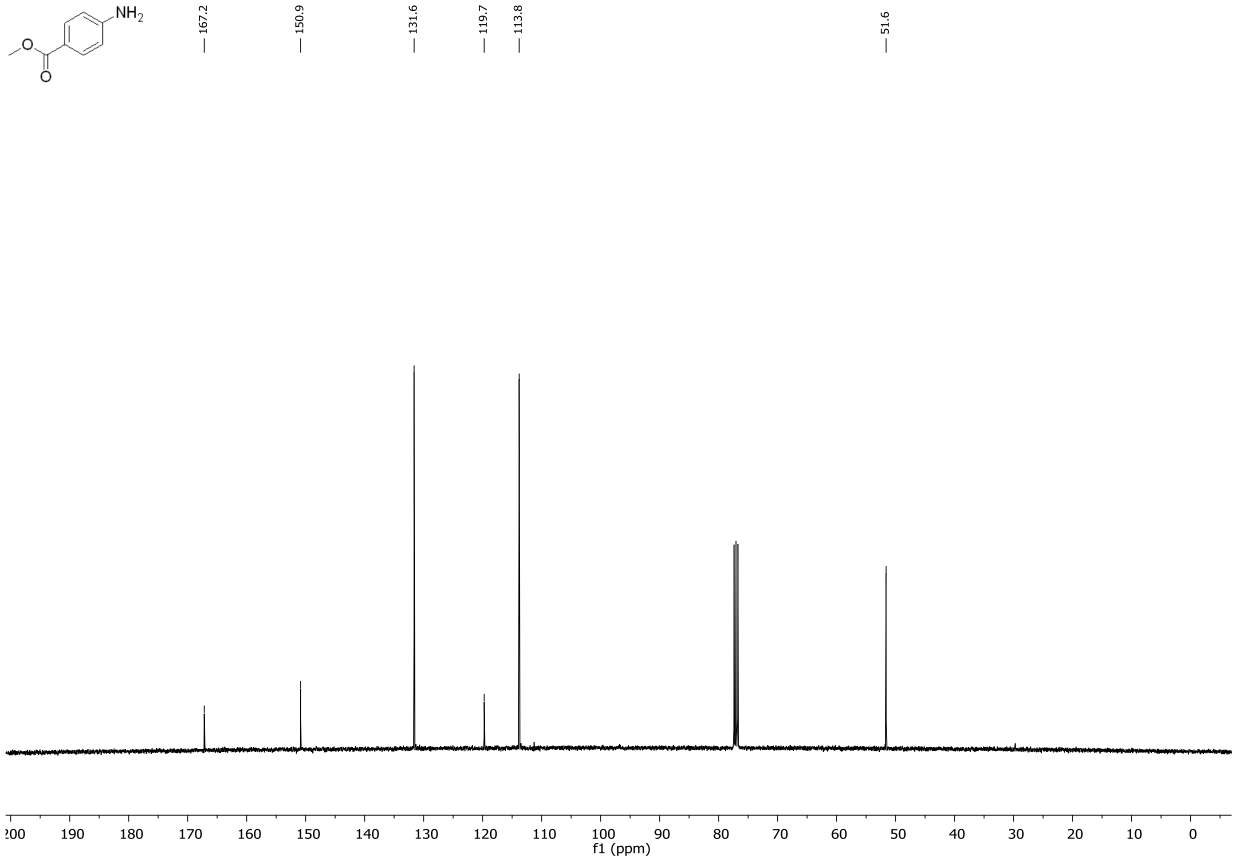

^{1}H NMR spectra of **2q** ($CDCl_3$, 600 MHz)

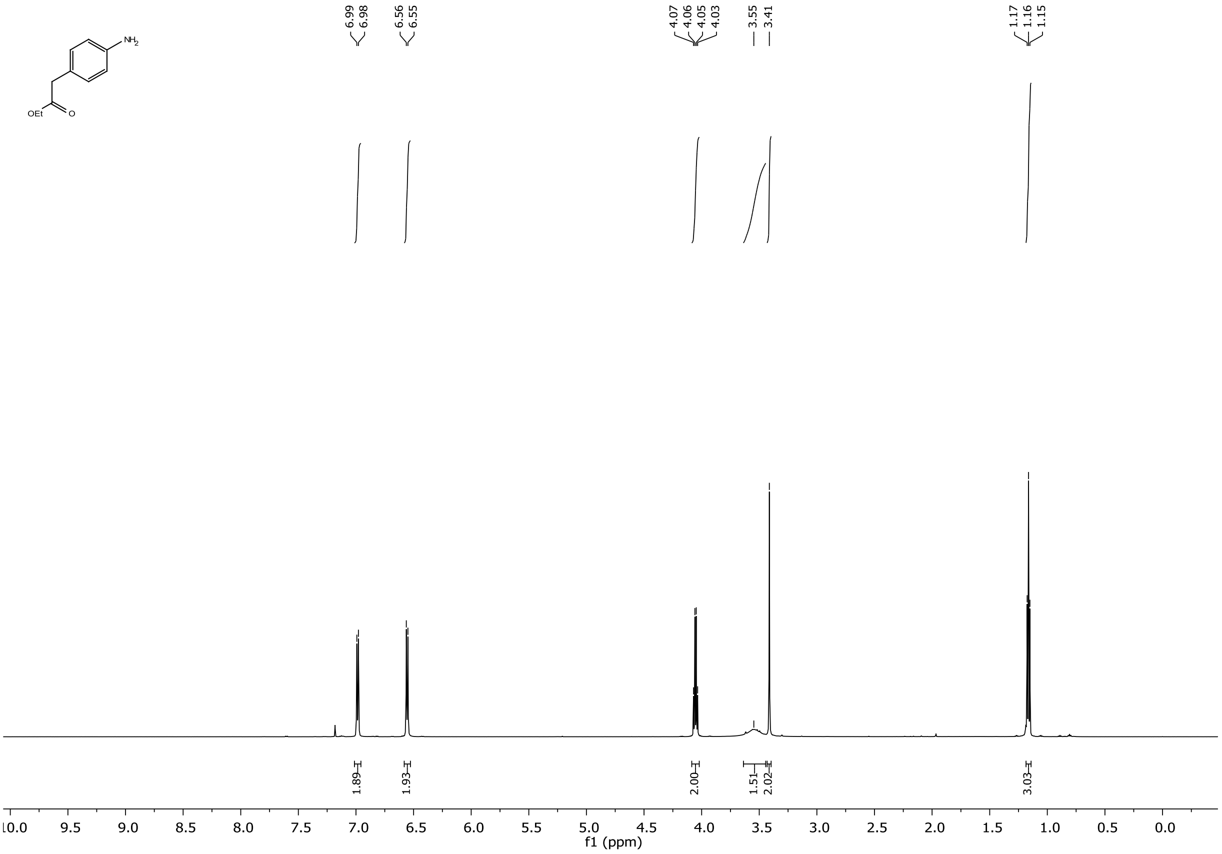

^{13}C NMR spectra of **2q** ($CDCl_3$, 151 MHz)

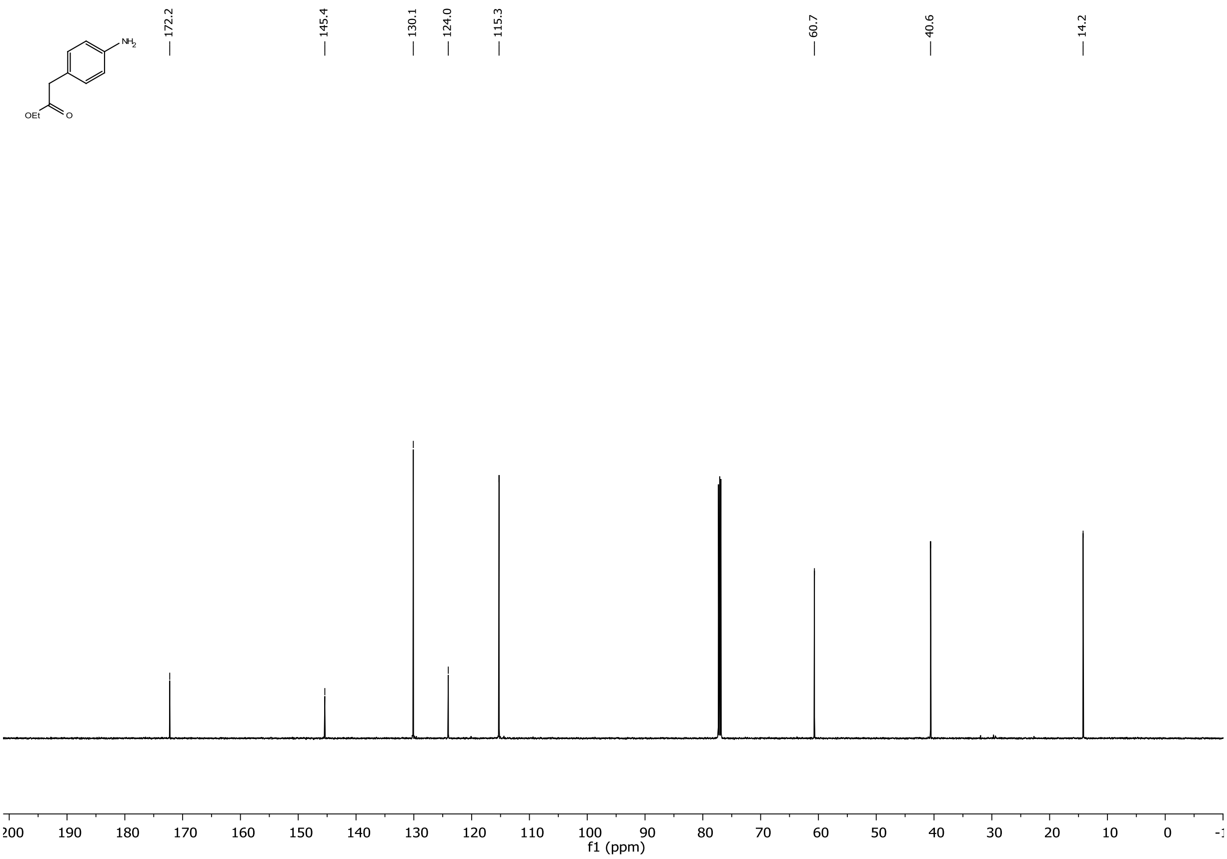

^{1}H NMR spectra of **2r** ($CDCl_3$, 600 MHz)

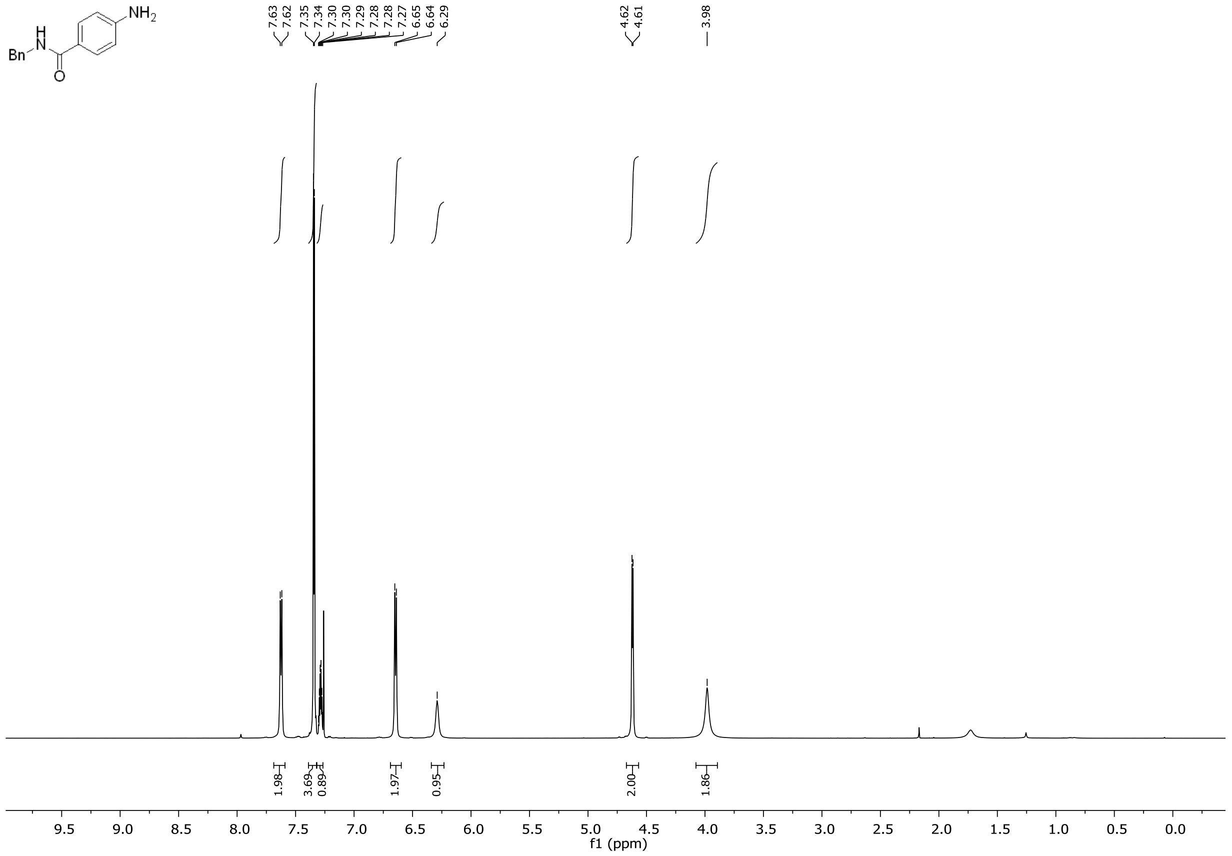

^{13}C NMR spectra of **2r** ($CDCl_3$, 151 MHz)

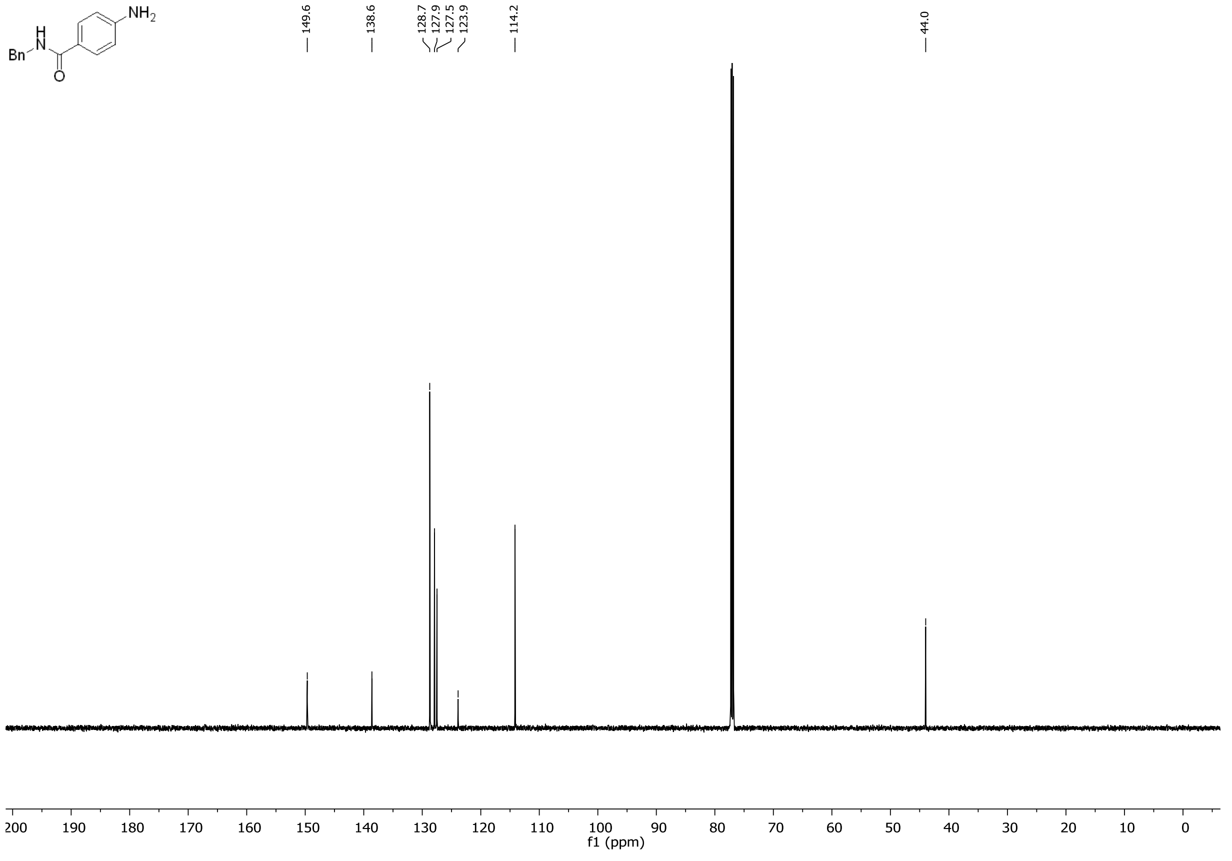

^{1}H NMR spectra of **2s** ($CDCl_3$, 400 MHz)

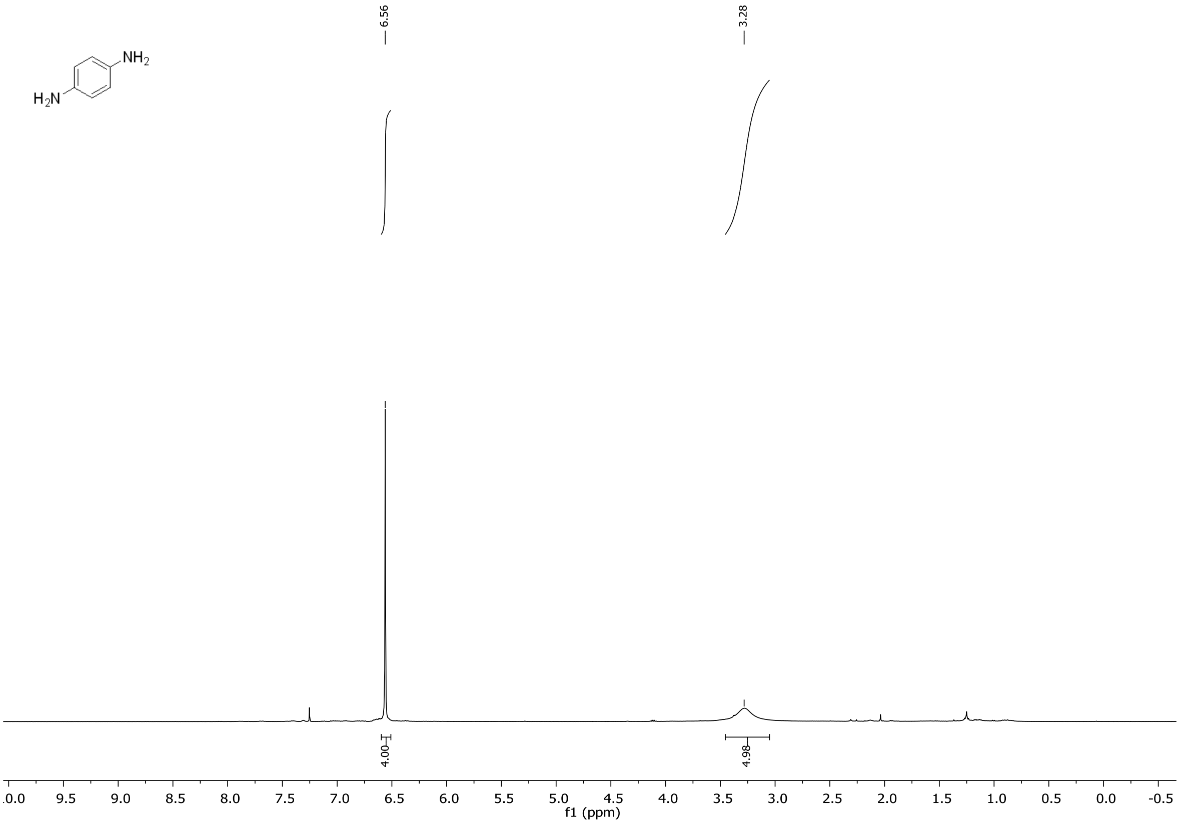

^{13}C NMR spectra of **2s** ($CDCl_3$, 101 MHz)

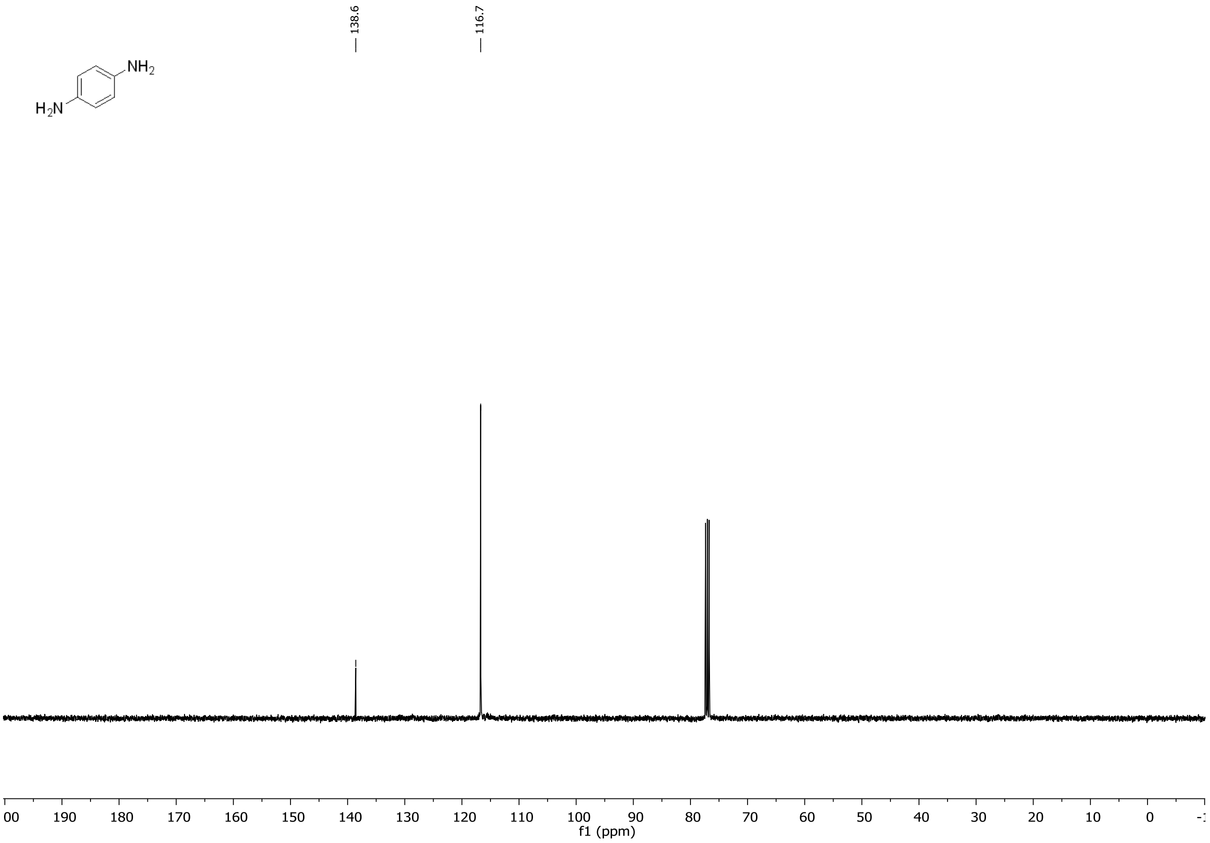

^{1}H NMR spectra of **2t** ($CDCl_3$, 600 MHz)

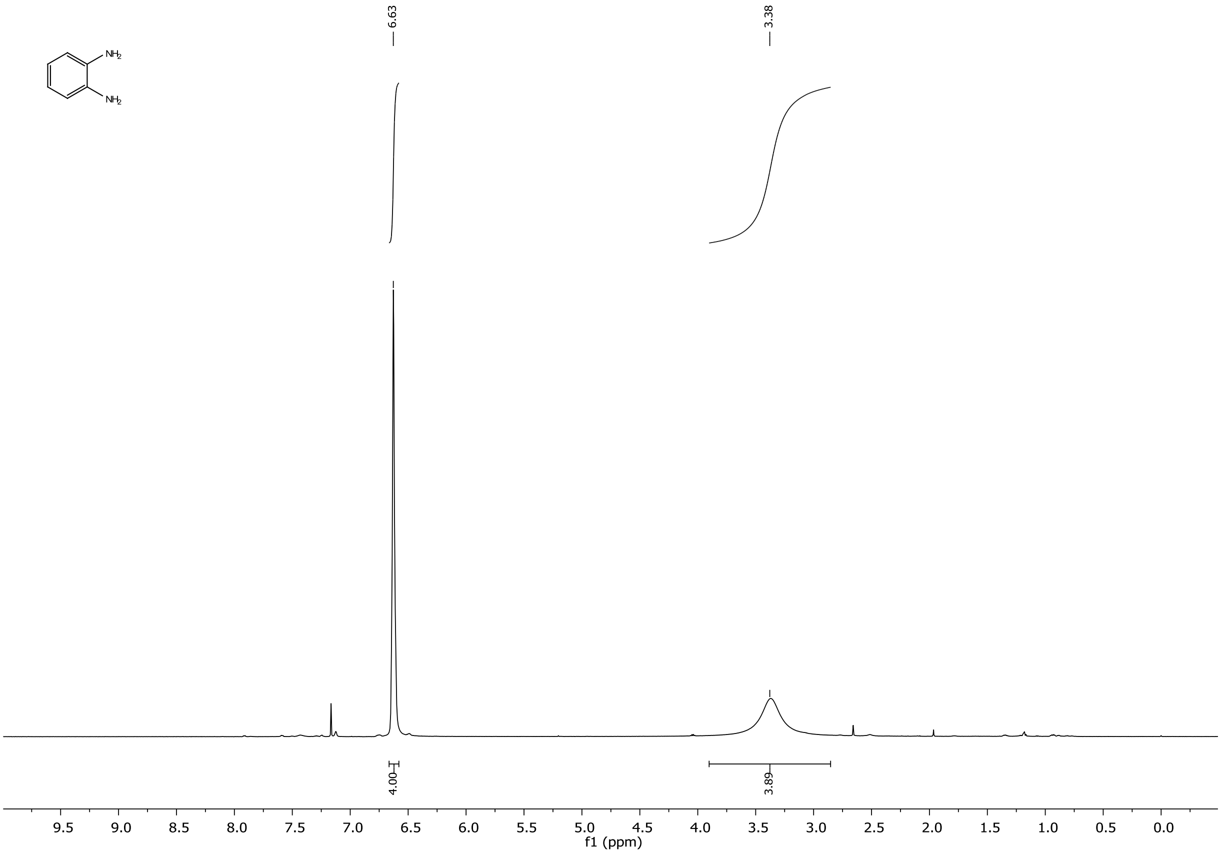

^{13}C NMR spectra of **2t** ($CDCl_3$, 151 MHz)

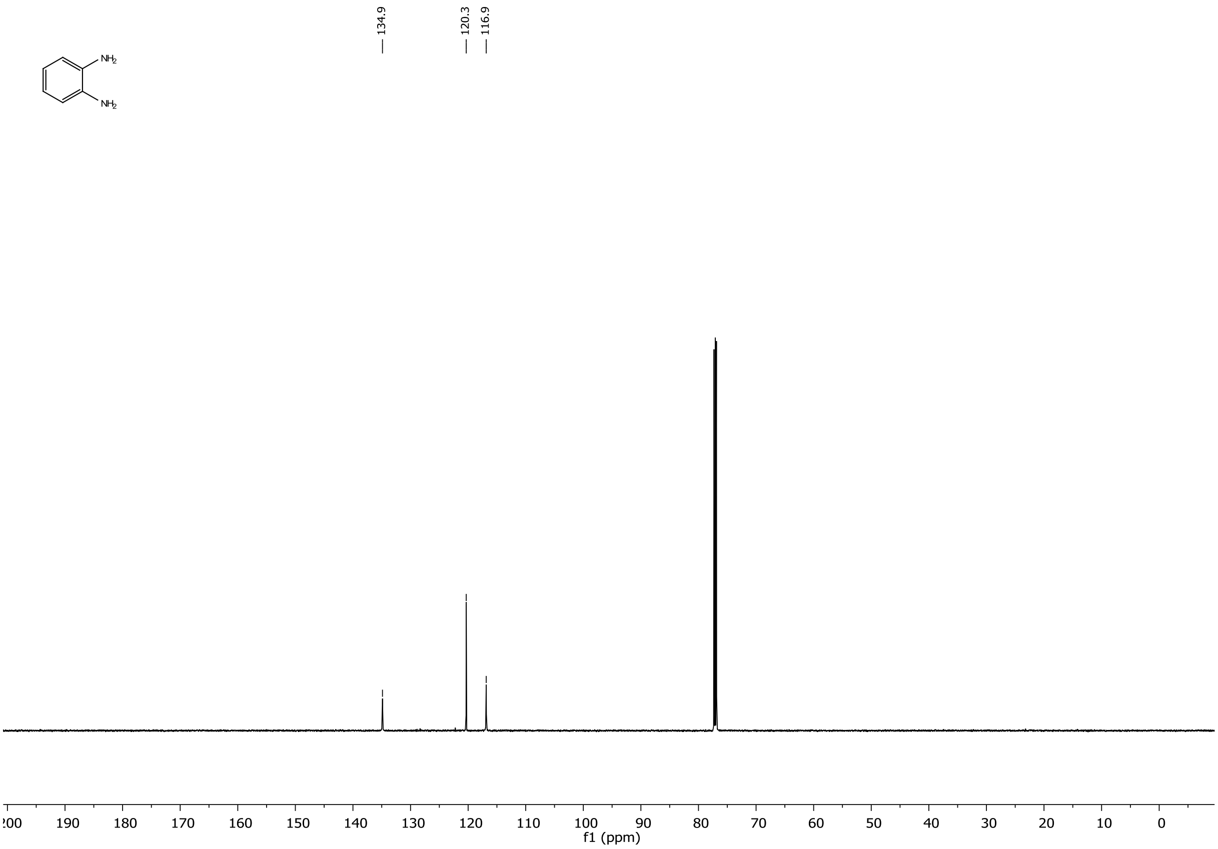

^{1}H NMR spectra of **2u** ($CDCl_3$, 600 MHz)

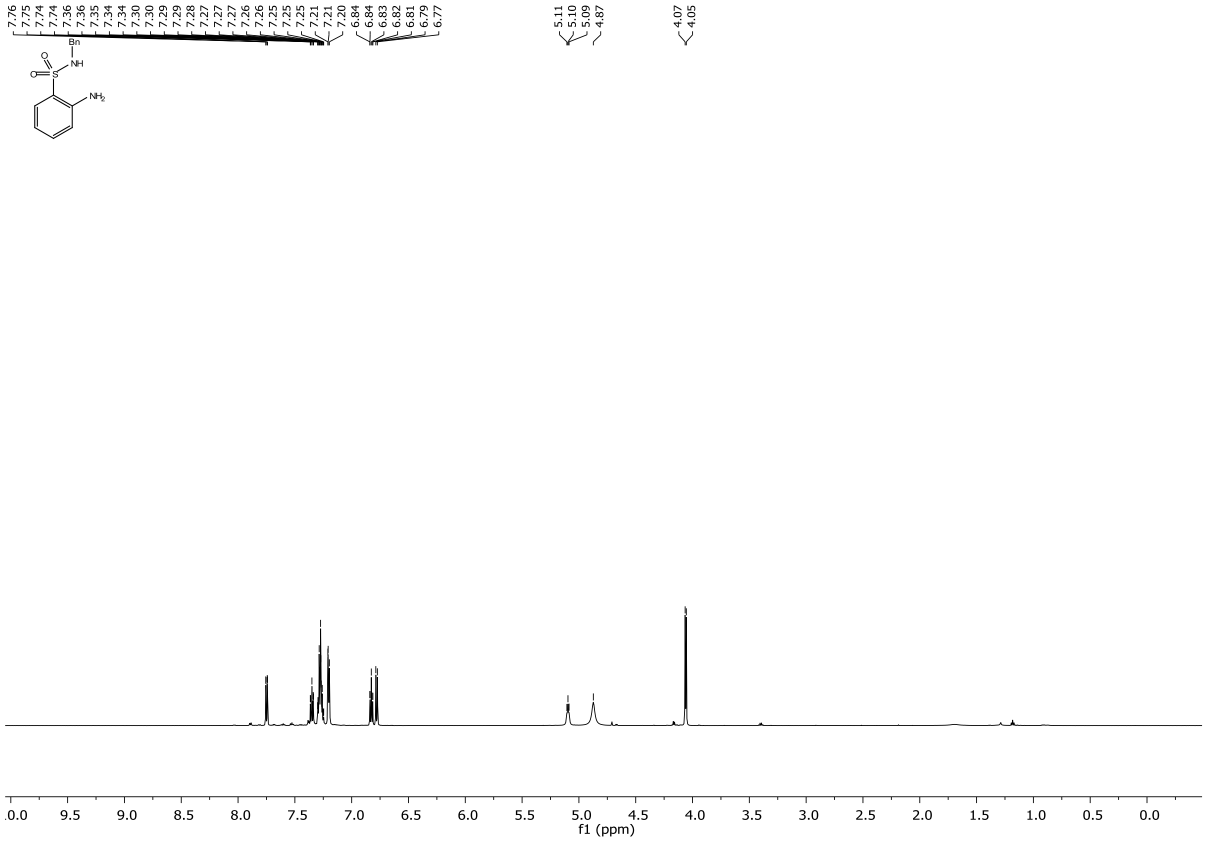

^{13}C NMR spectra of **2u** ($CDCl_3$, 151 MHz)

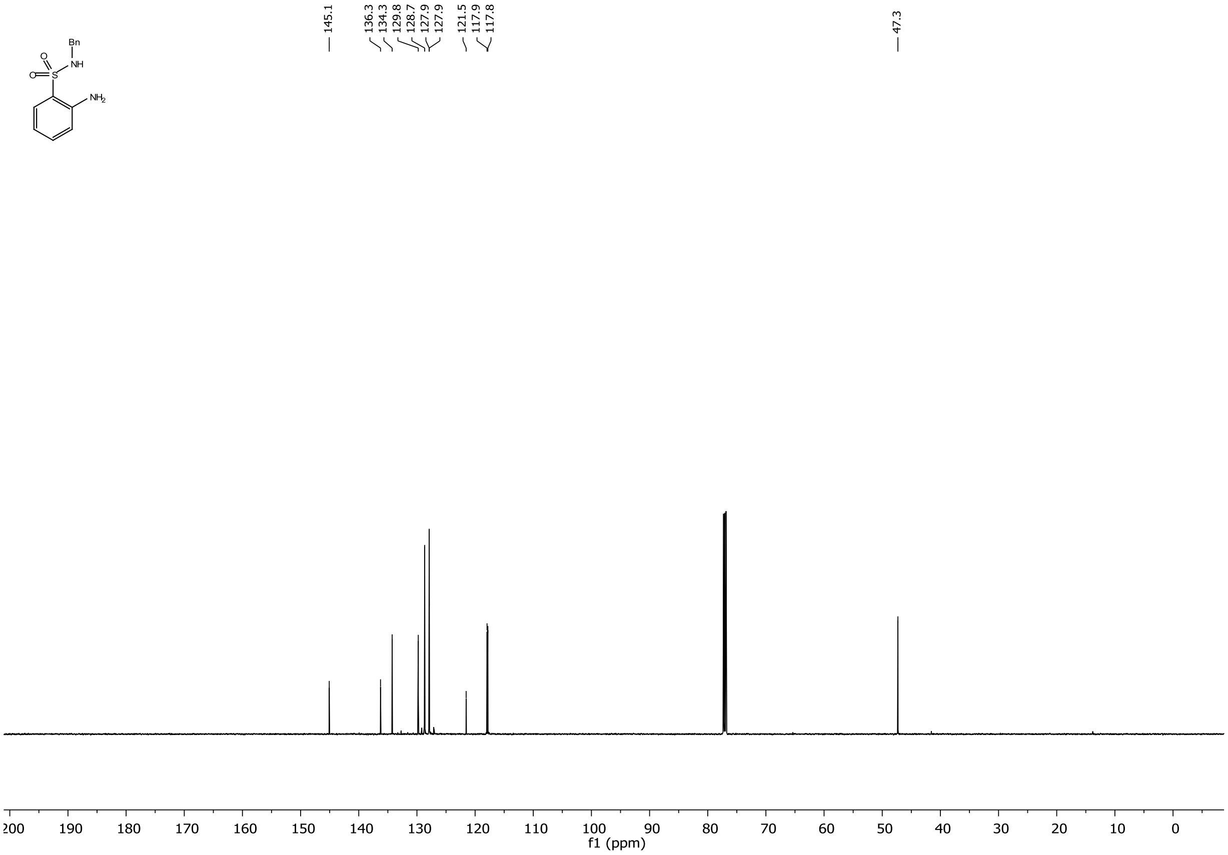

^{1}H NMR spectra of **2v** ($CDCl_3$, 400 MHz)

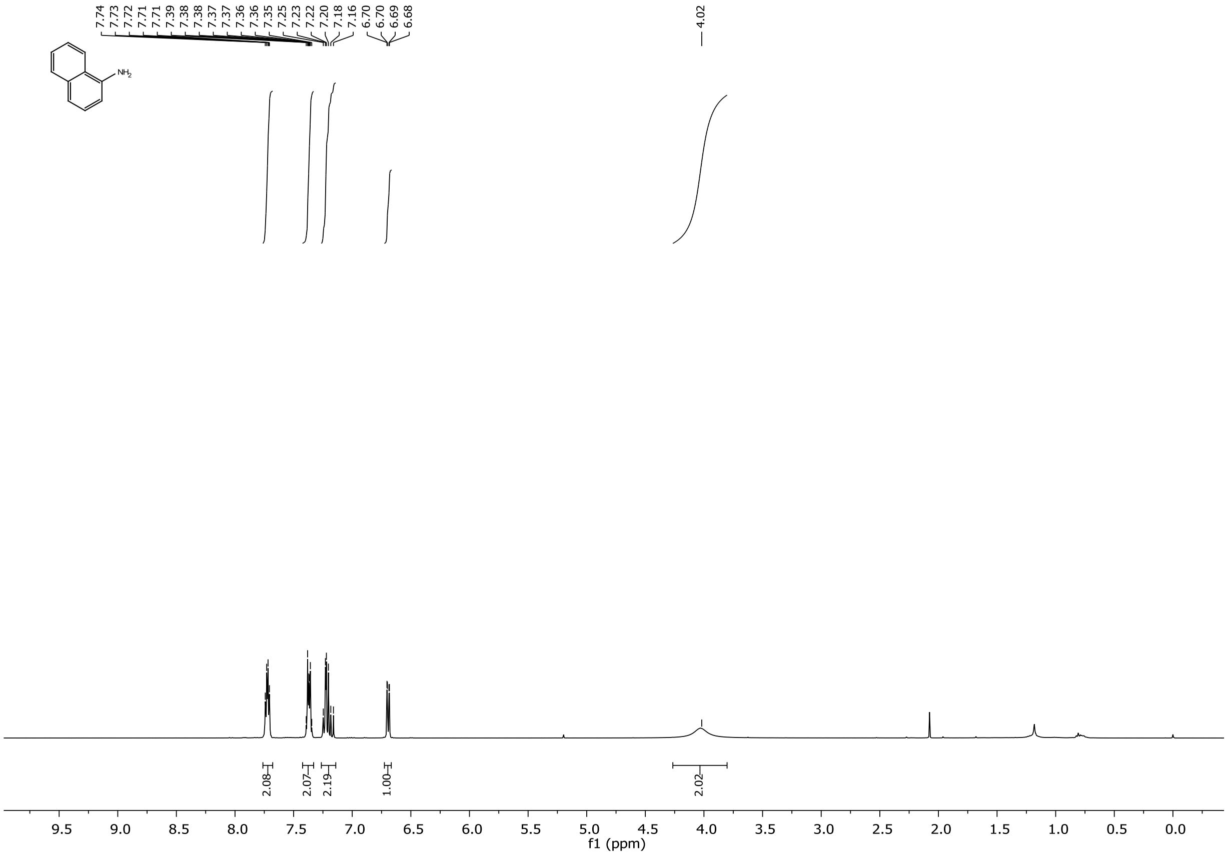

^{13}C NMR spectra of **2v** ($CDCl_3$, 101 MHz)

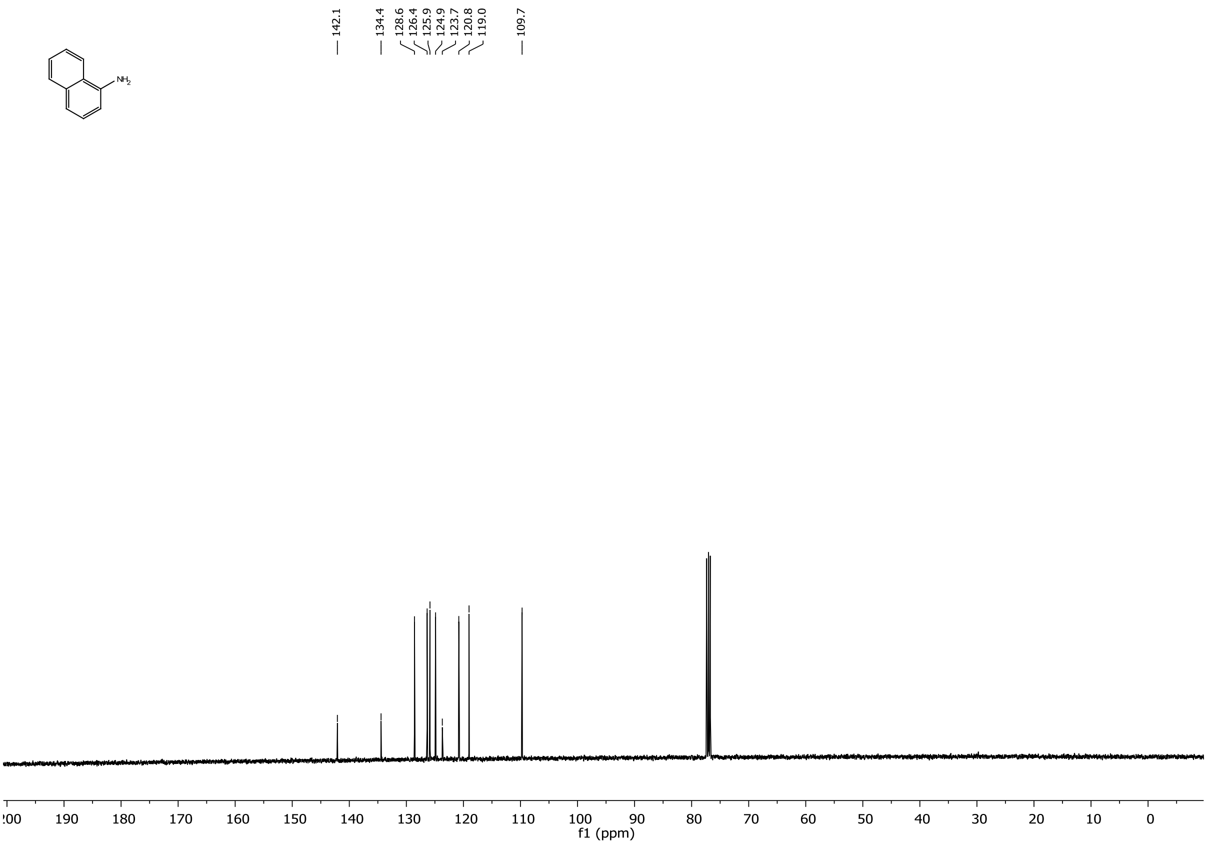

^{1}H NMR spectra of **1w** ($CDCl_3$, 600 MHz)

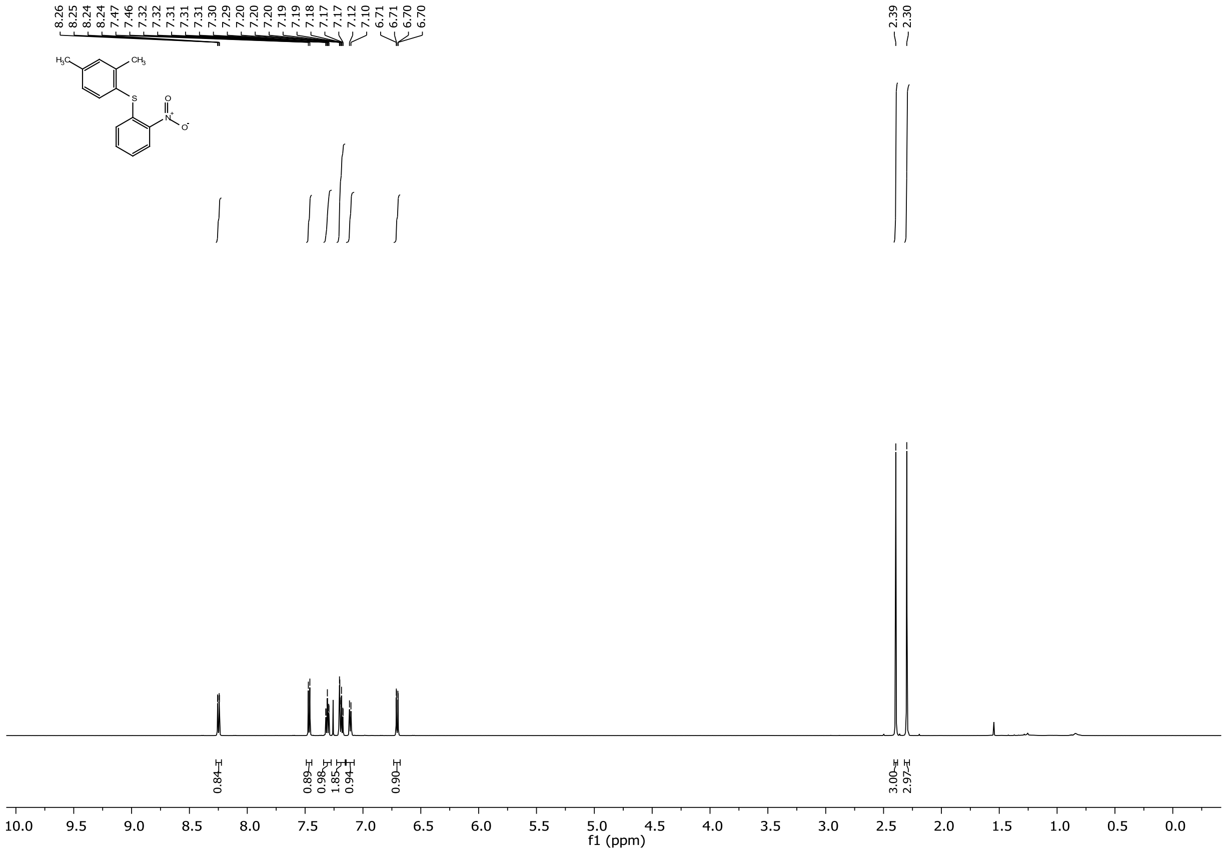

^{13}C NMR spectra of **1w** ($CDCl_3$, 151 MHz)

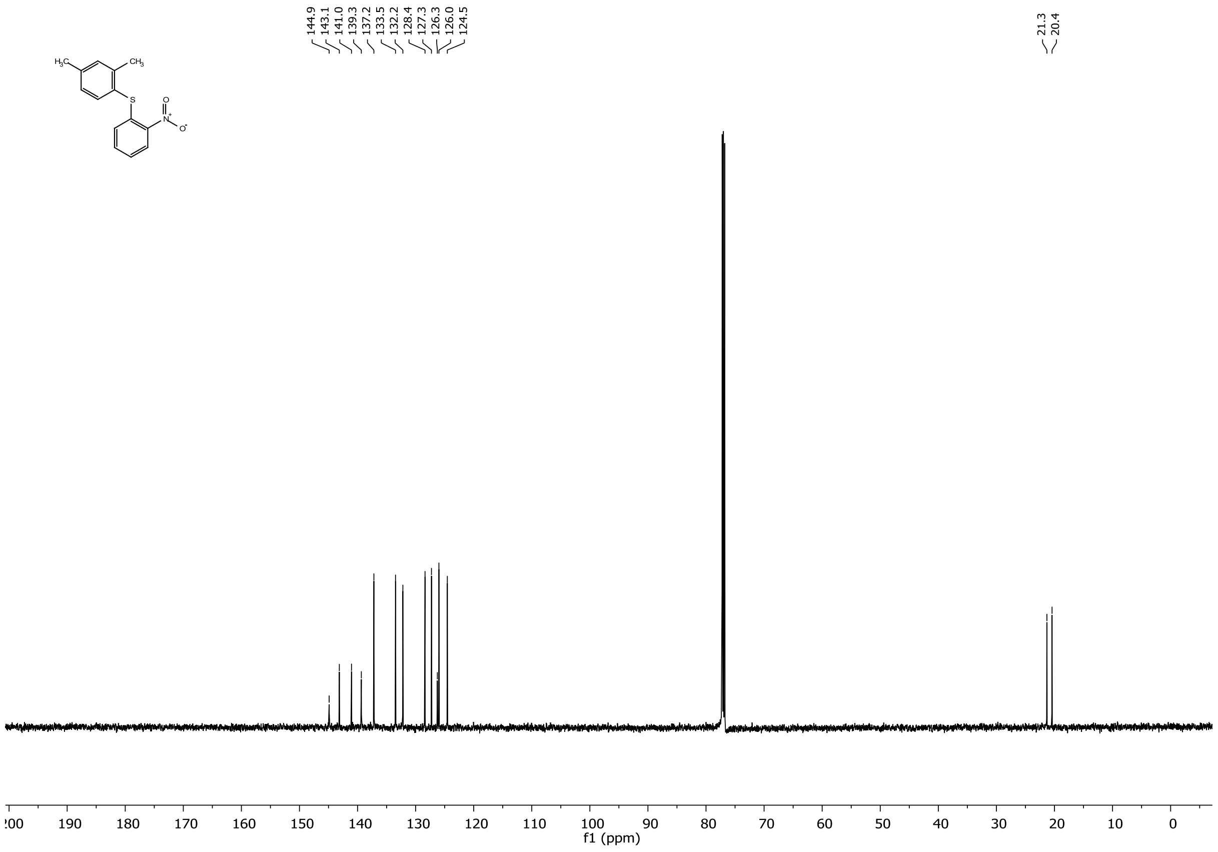

^{1}H NMR spectra of **2w** ($CDCl_3$, 600 MHz)

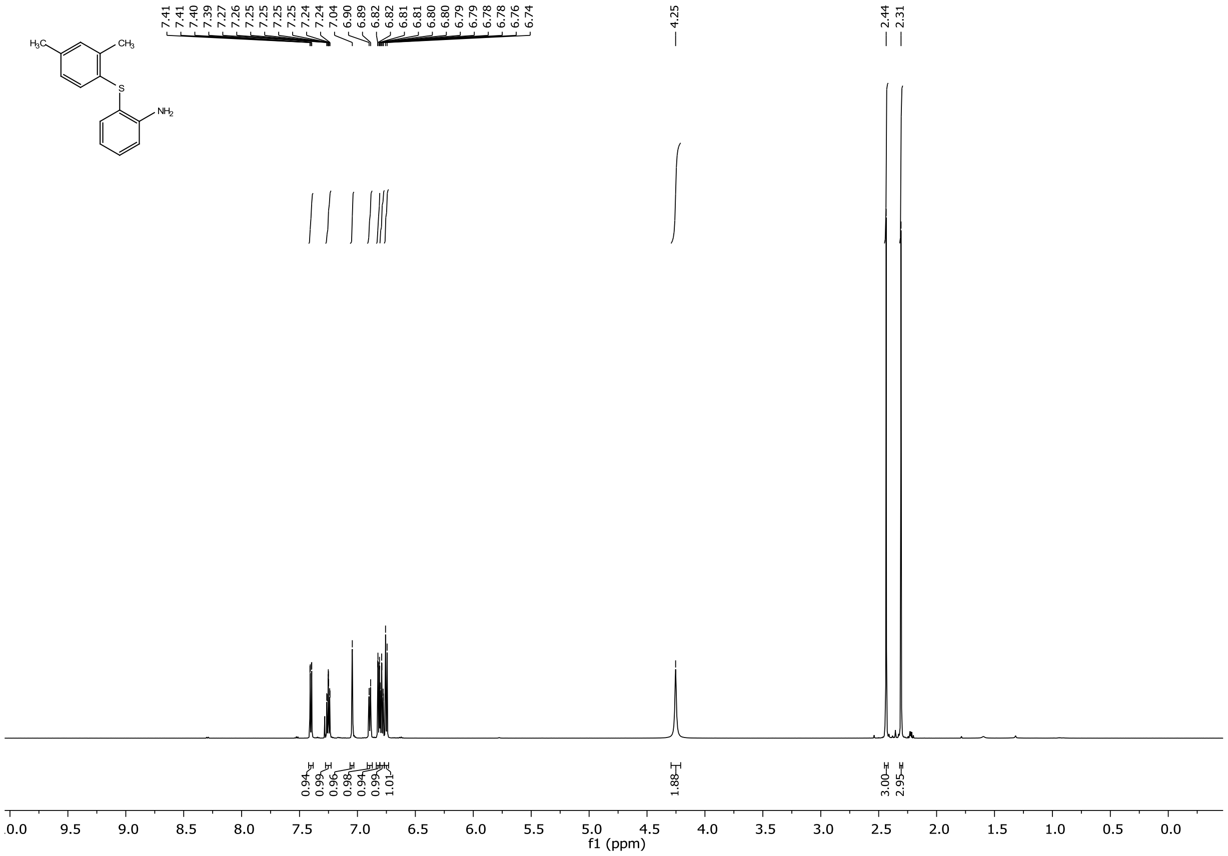

^{13}C NMR spectra of **2w** ($CDCl_3$, 151 MHz)

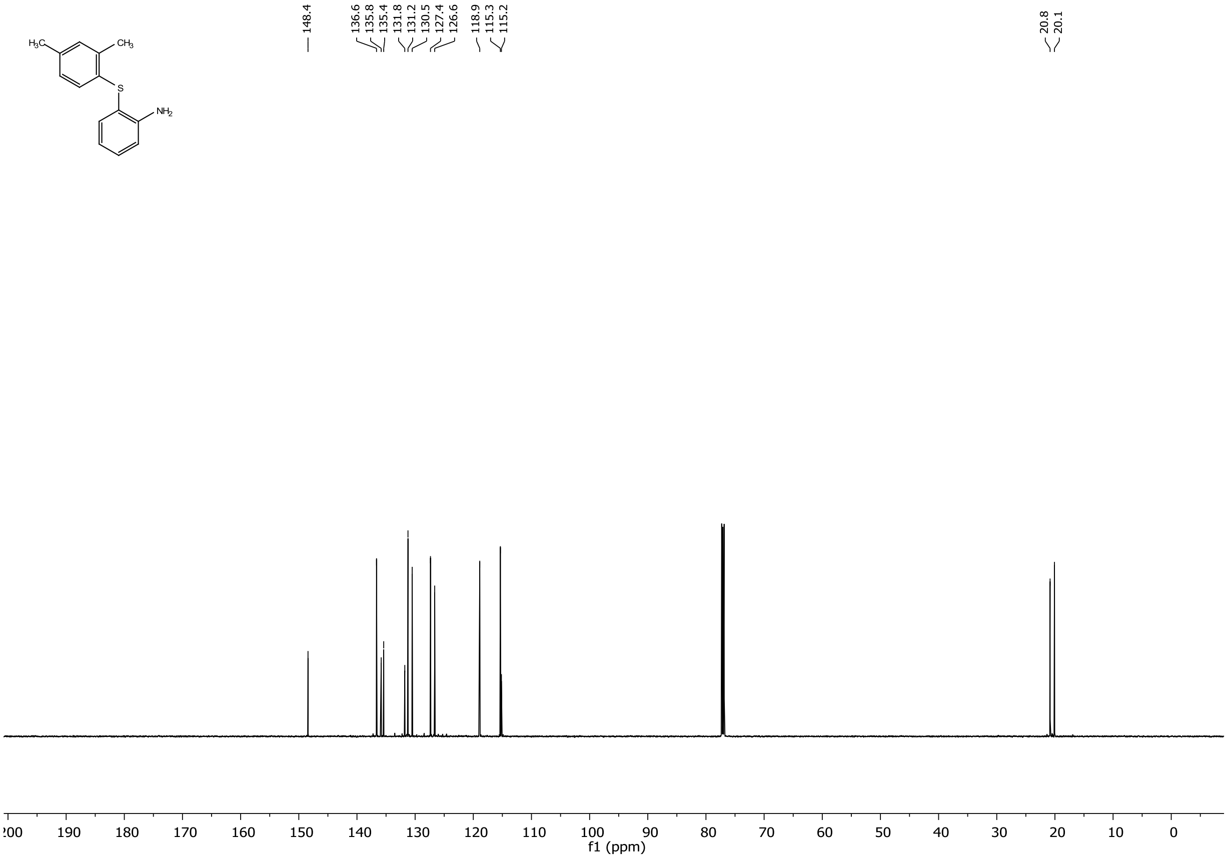

^{1}H NMR spectra of **5a** ($CDCl_3$, 400 MHz)

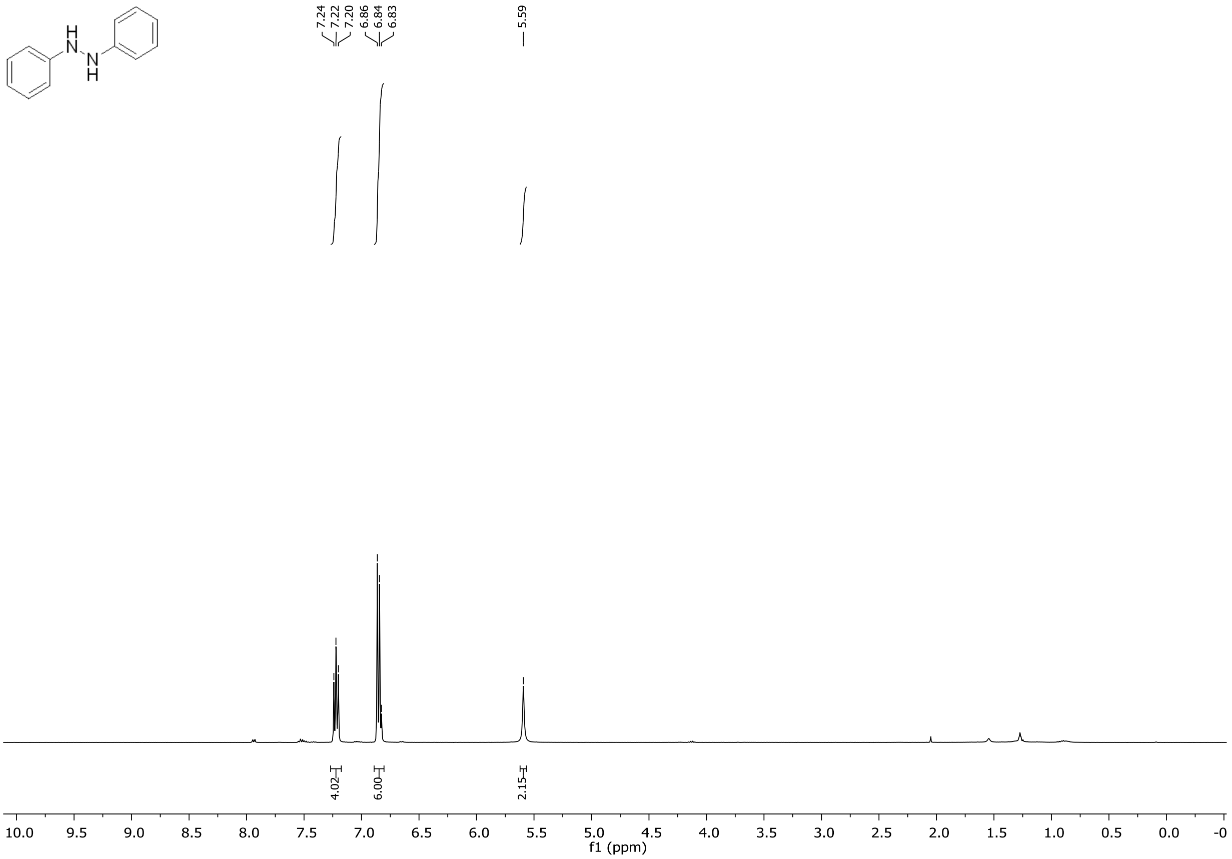

^{13}C NMR spectra of **5a** ($CDCl_3$, 101 MHz)

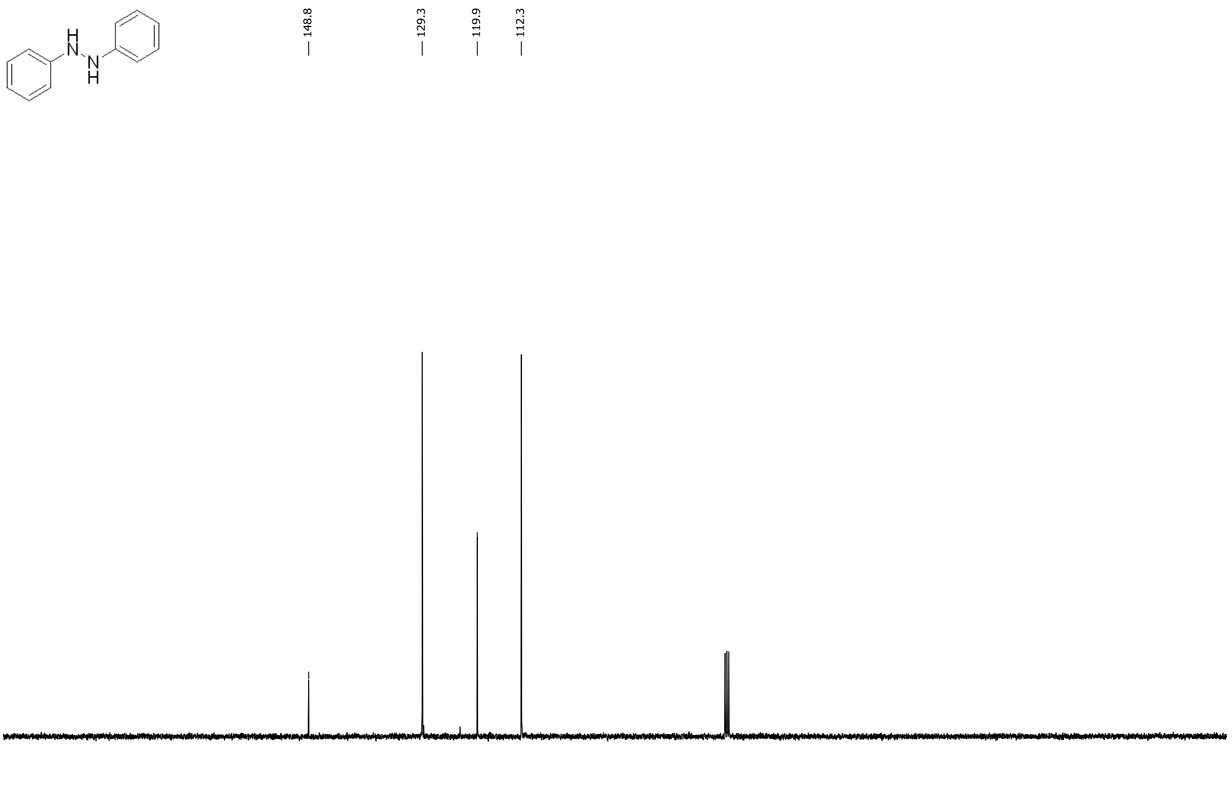

^{1}H NMR spectra of **5b** ($CDCl_3$, 400 MHz)

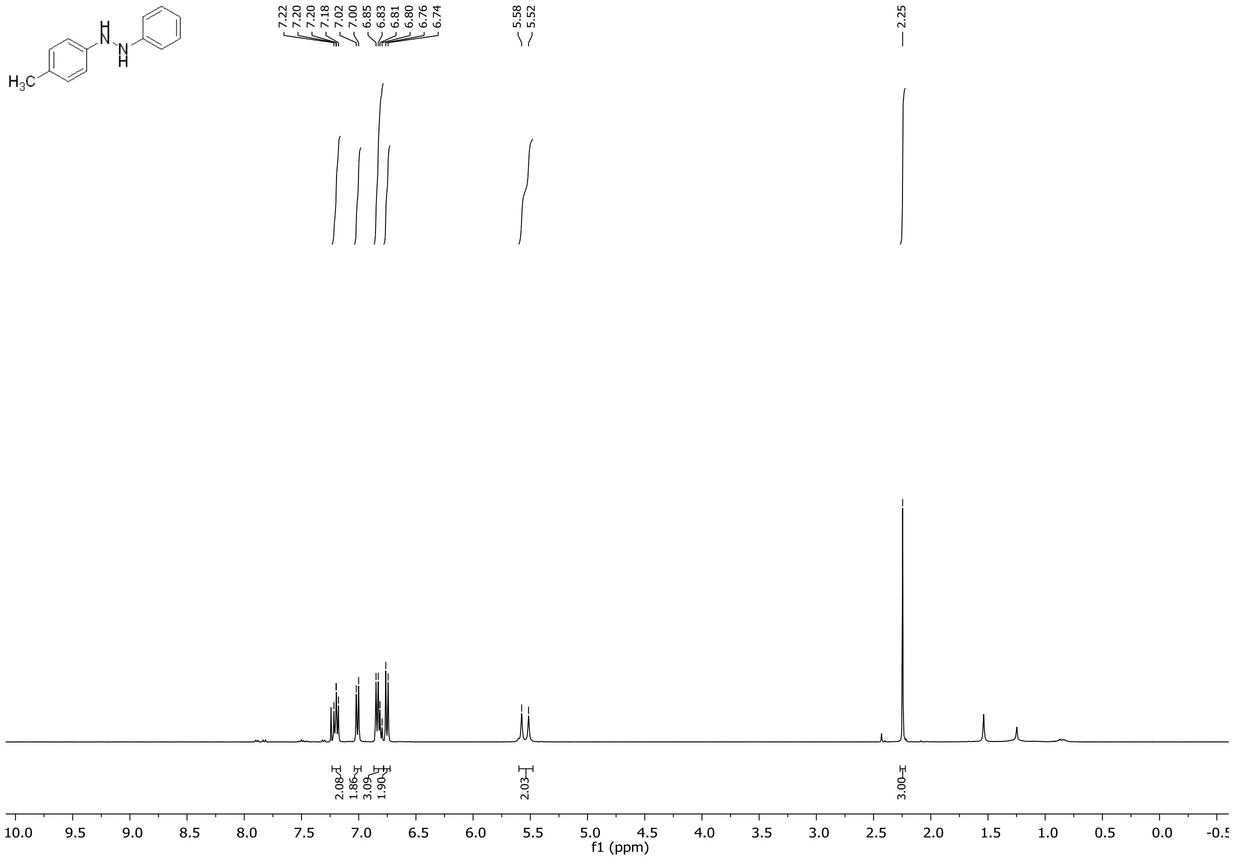

^{13}C NMR spectra of **5b** ($CDCl_3$, 101 MHz)

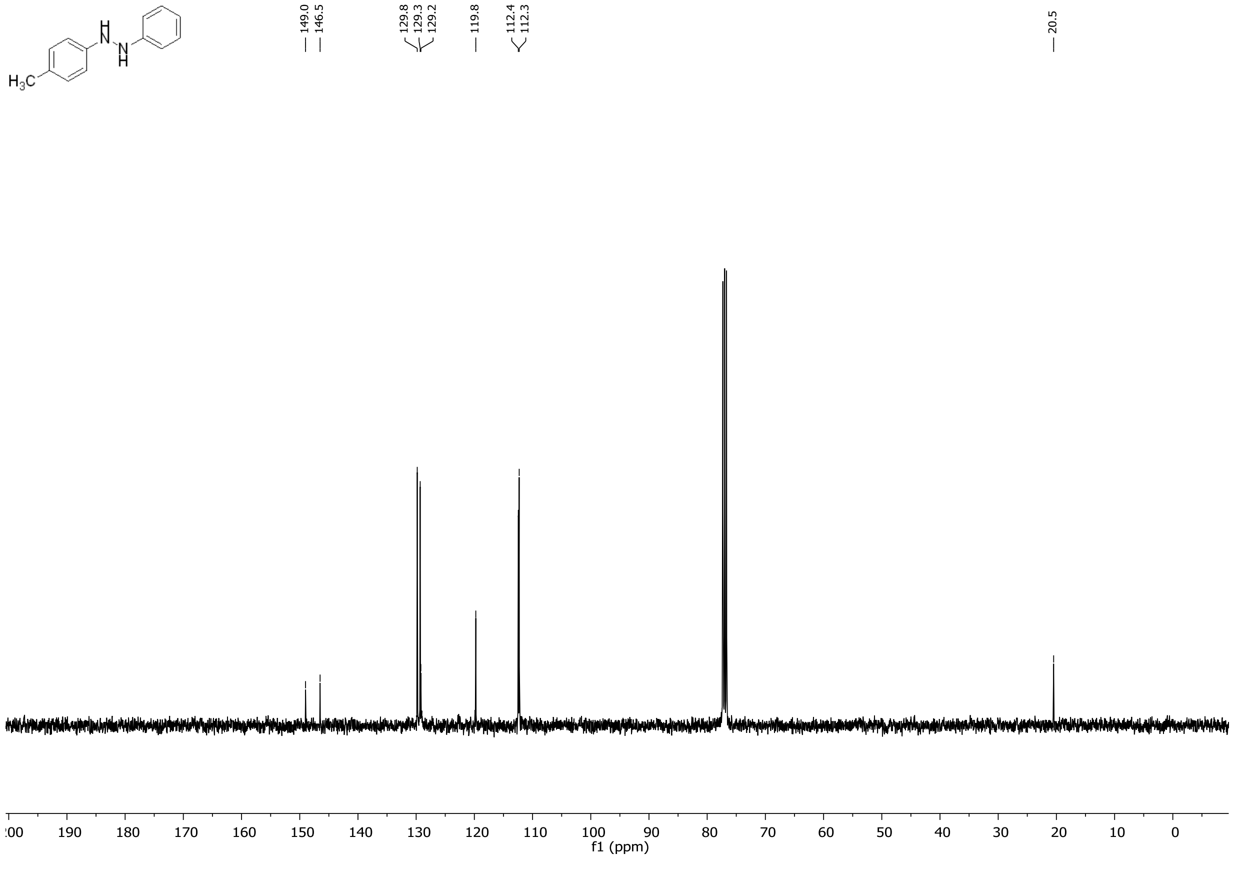

^{1}H NMR spectra of **5c** ($CDCl_3$, 400 MHz)

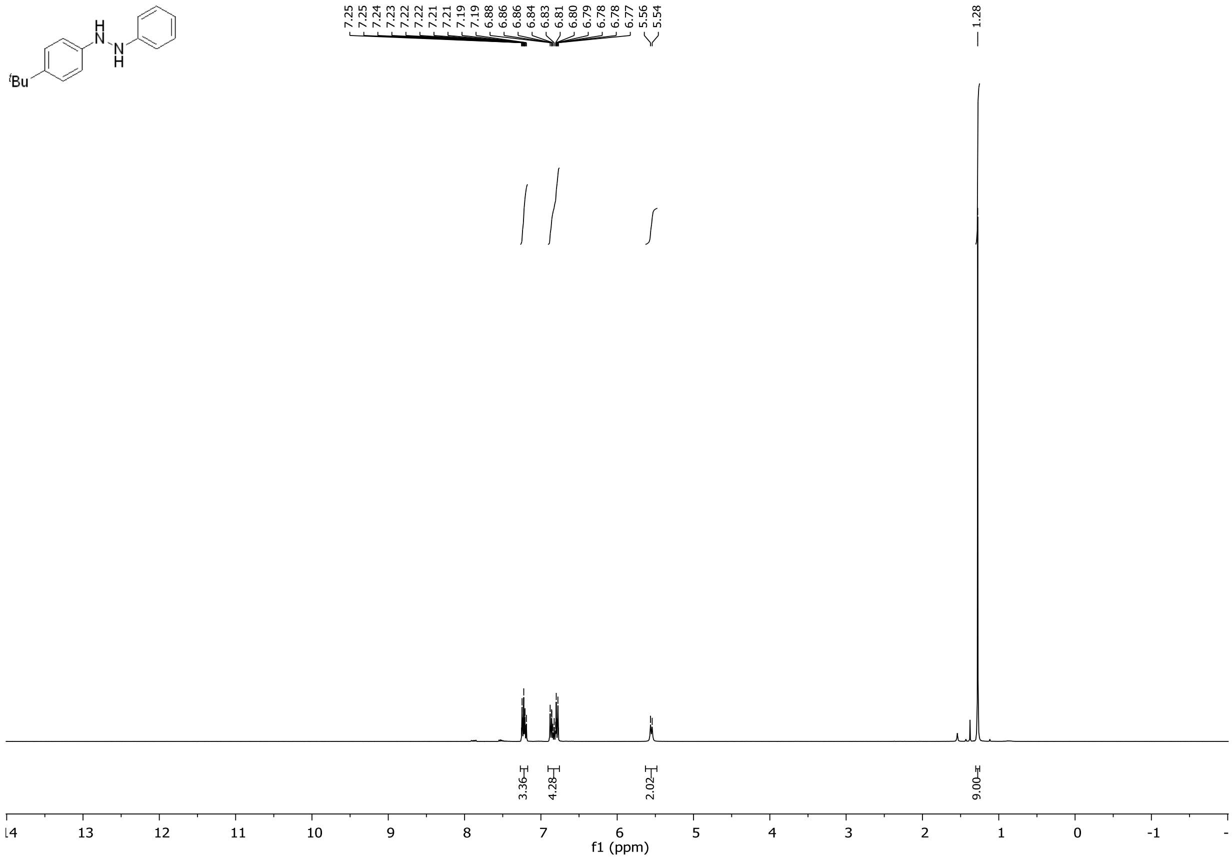

^{13}C NMR spectra of **5c** ($CDCl_3$, 101 MHz)

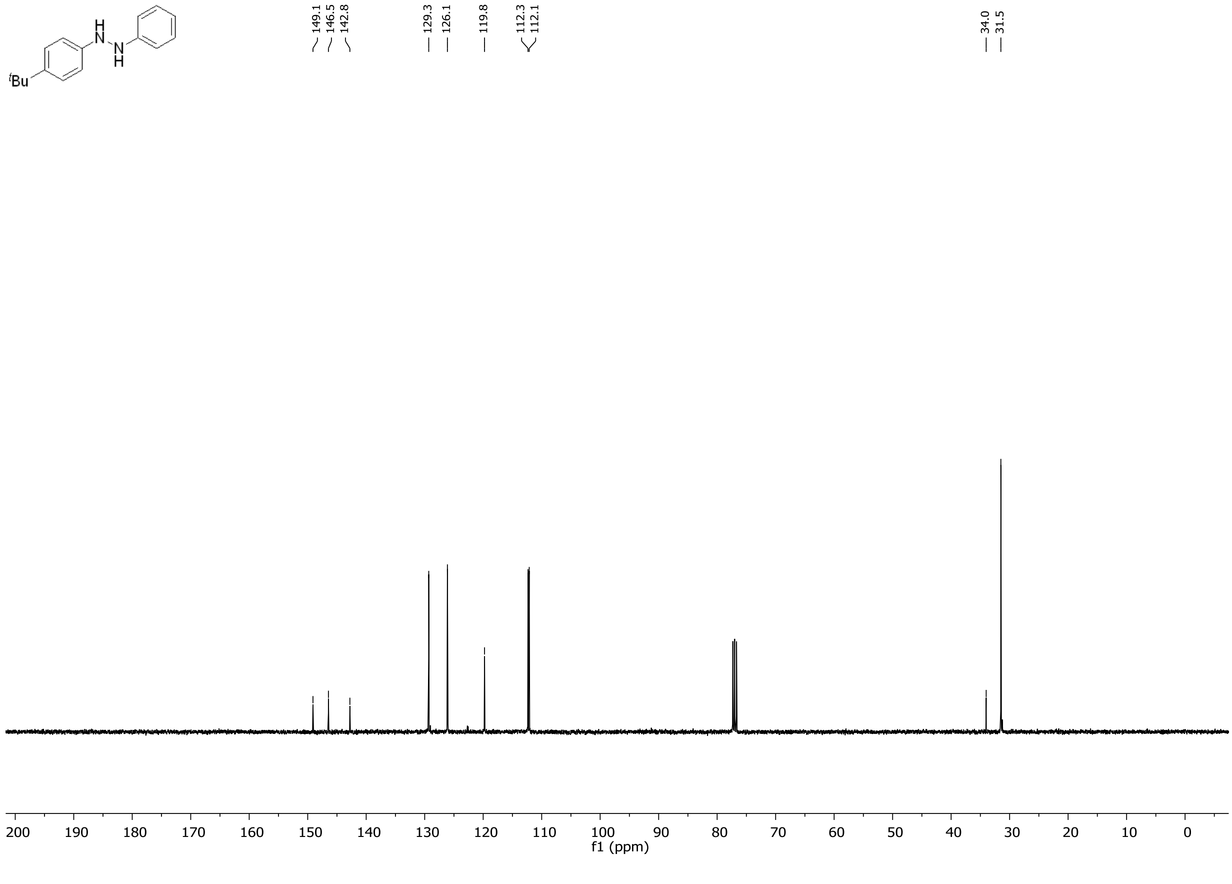

^{1}H NMR spectra of **5d** ($CDCl_3$, 600 MHz)

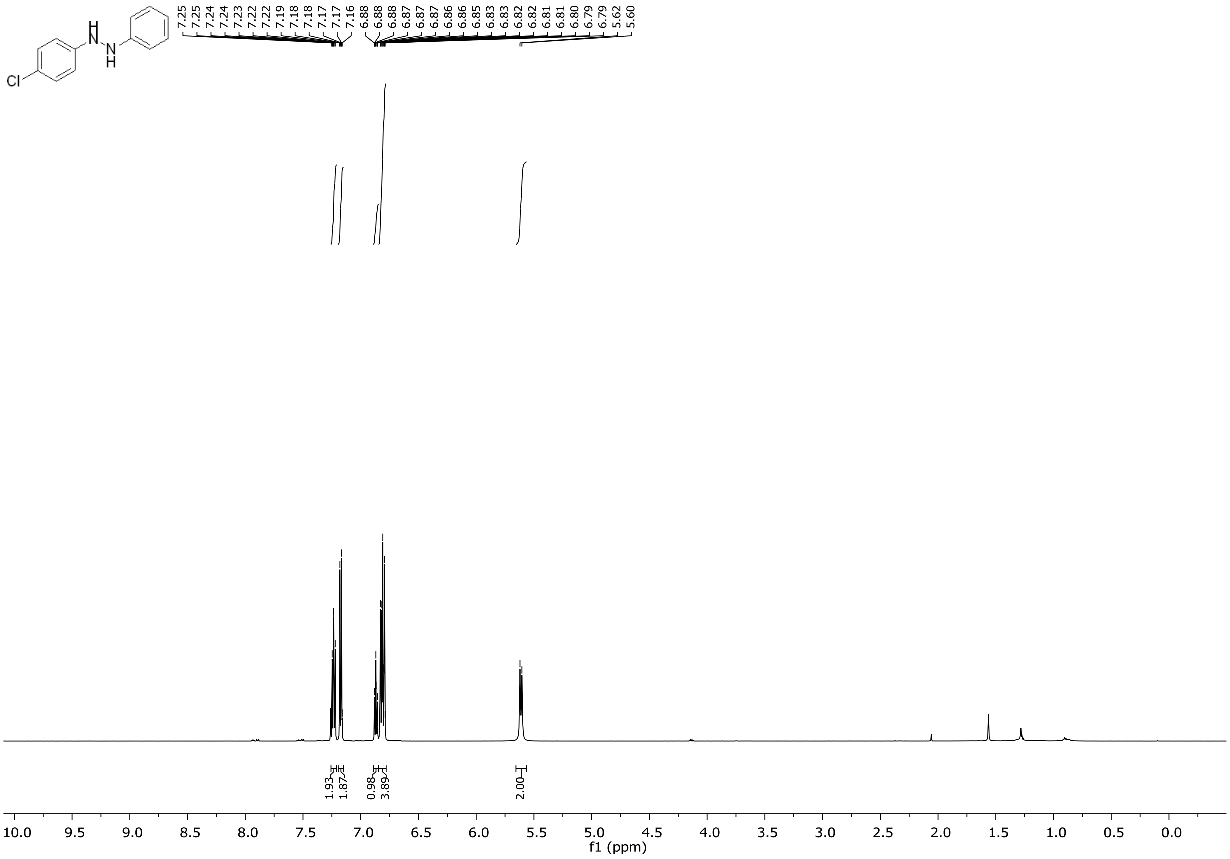

^{13}C NMR spectra of **5d** ($CDCl_3$, 151 MHz)

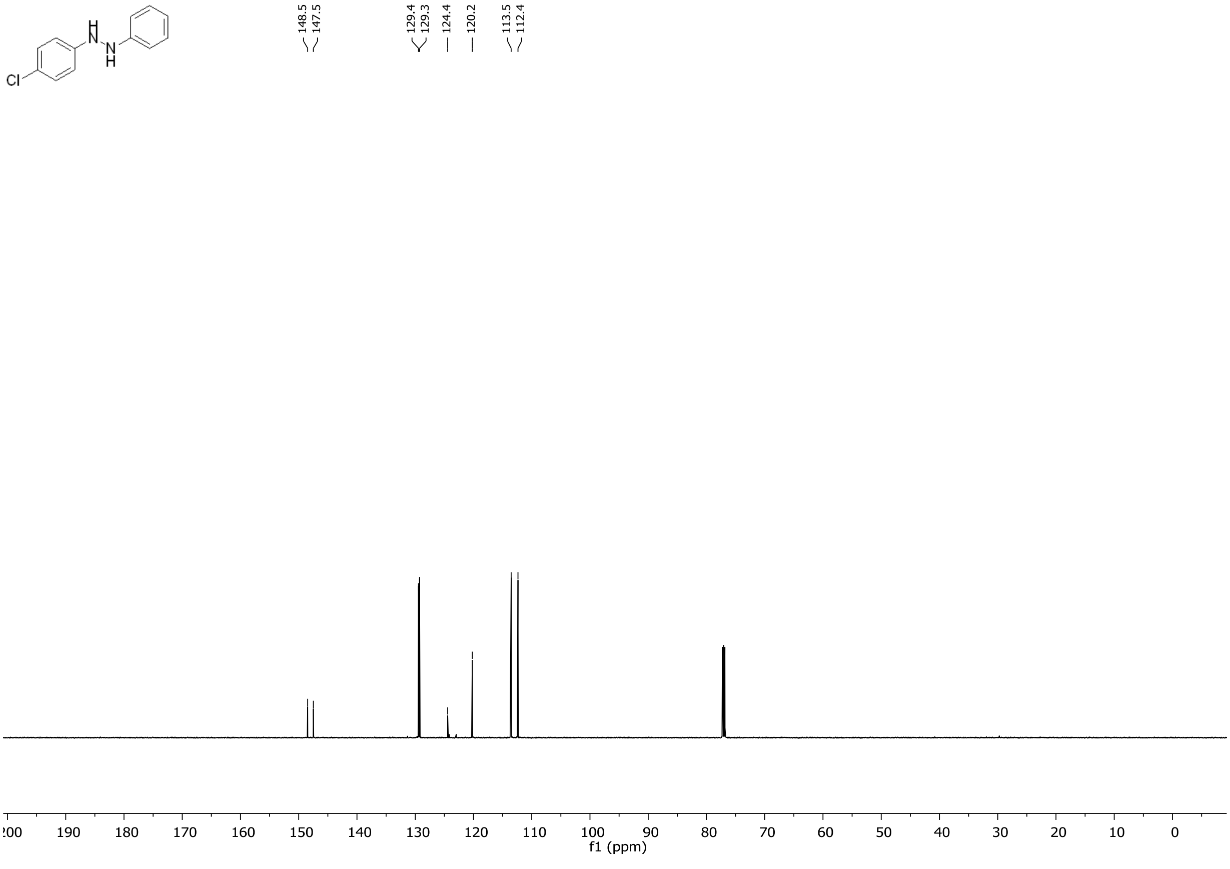

^{1}H NMR spectra of **5e** ($CDCl_3$, 600 MHz)

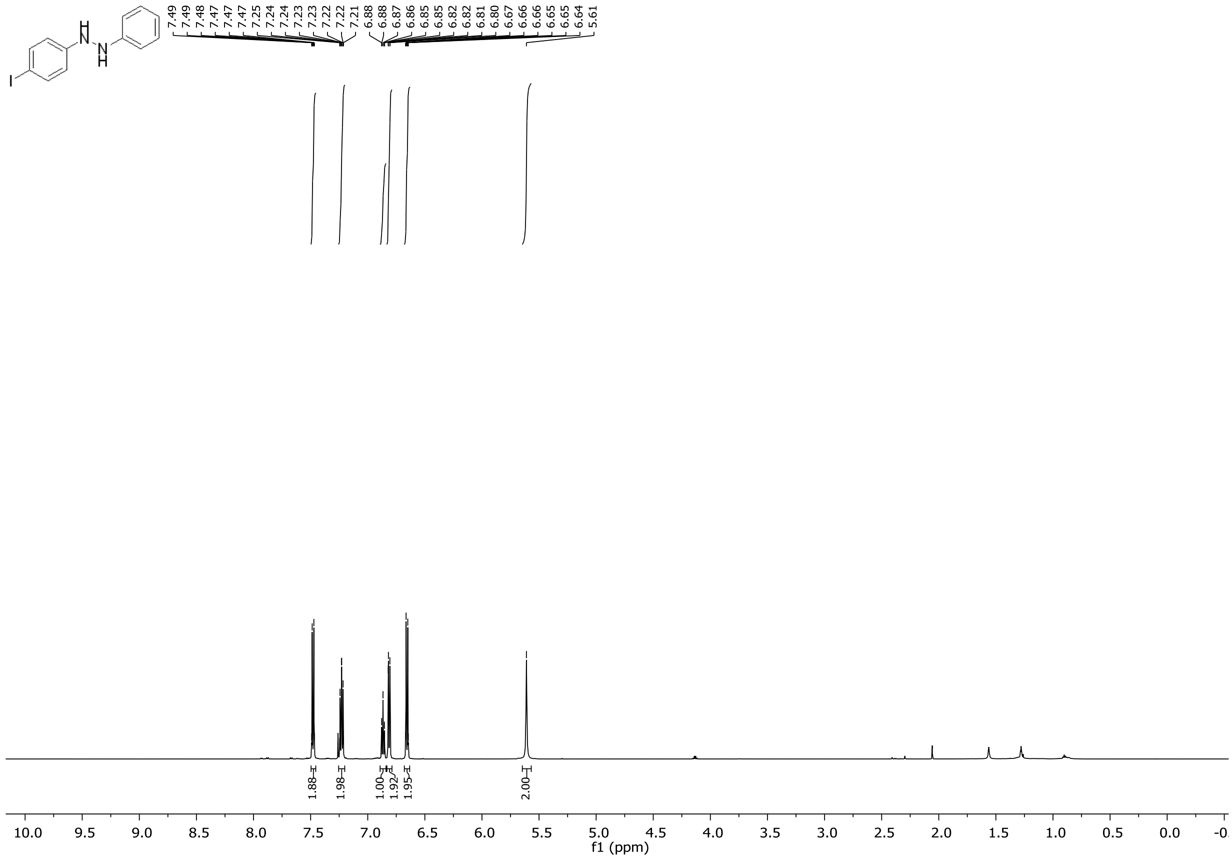

^{13}C NMR spectra of **5e** ($CDCl_3$, 151 MHz)

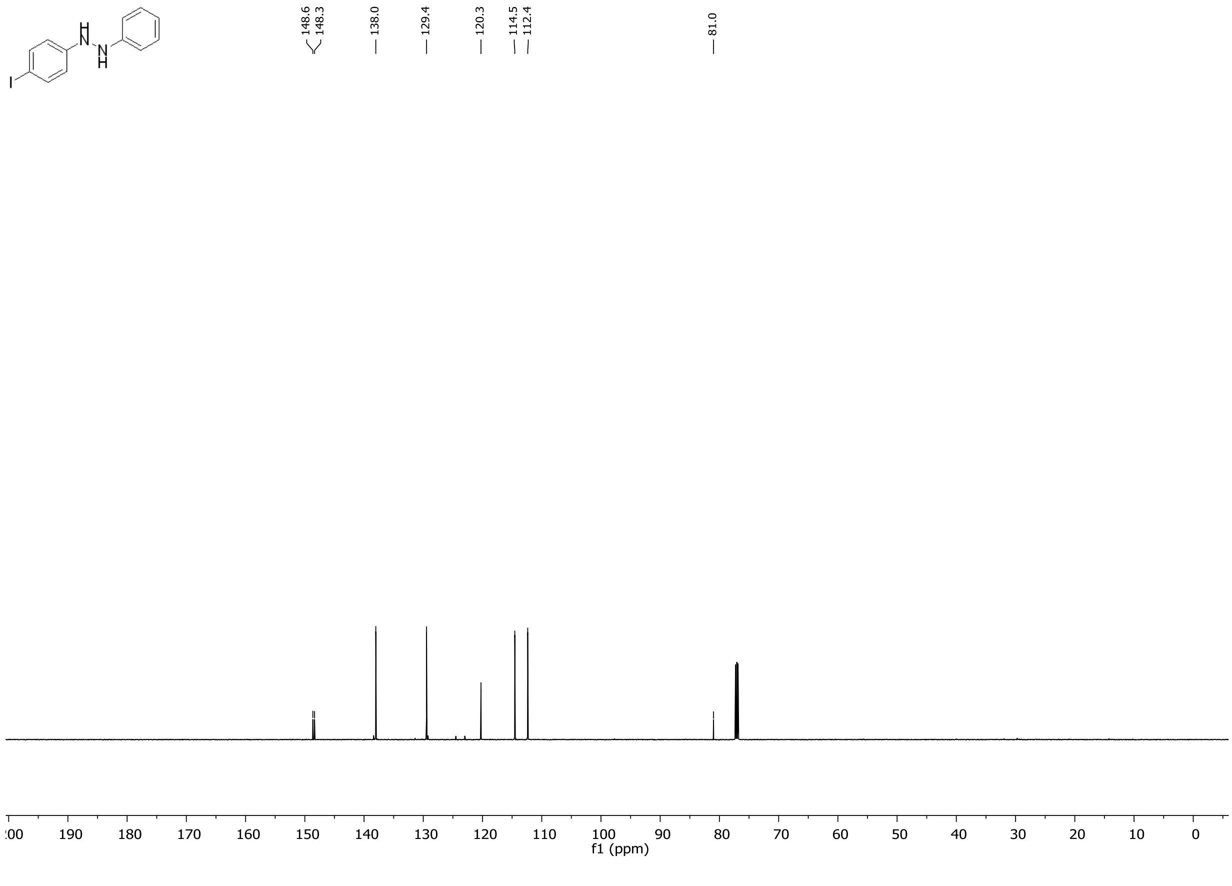

^{1}H NMR spectra of **5f** ($CDCl_3$, 400 MHz)

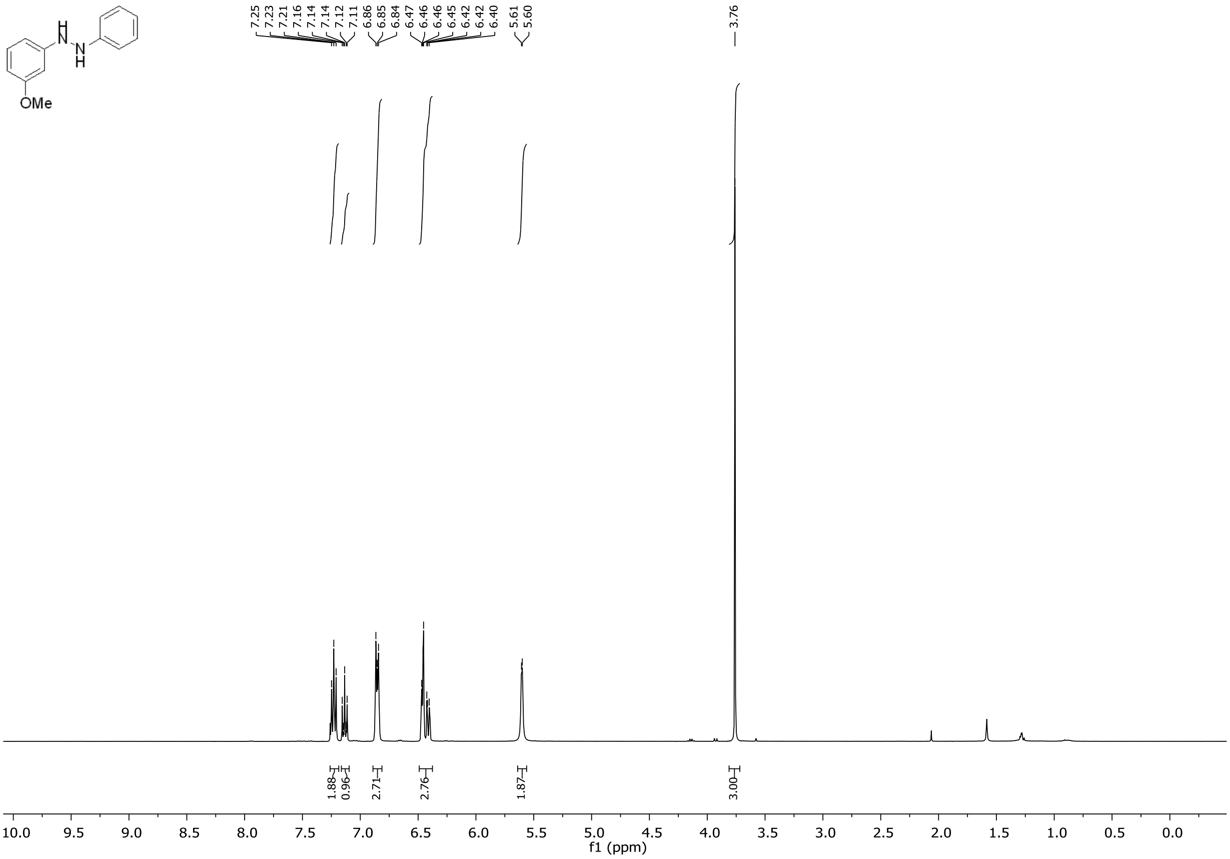

^{13}C NMR spectra of **5f** ($CDCl_3$, 101 MHz)

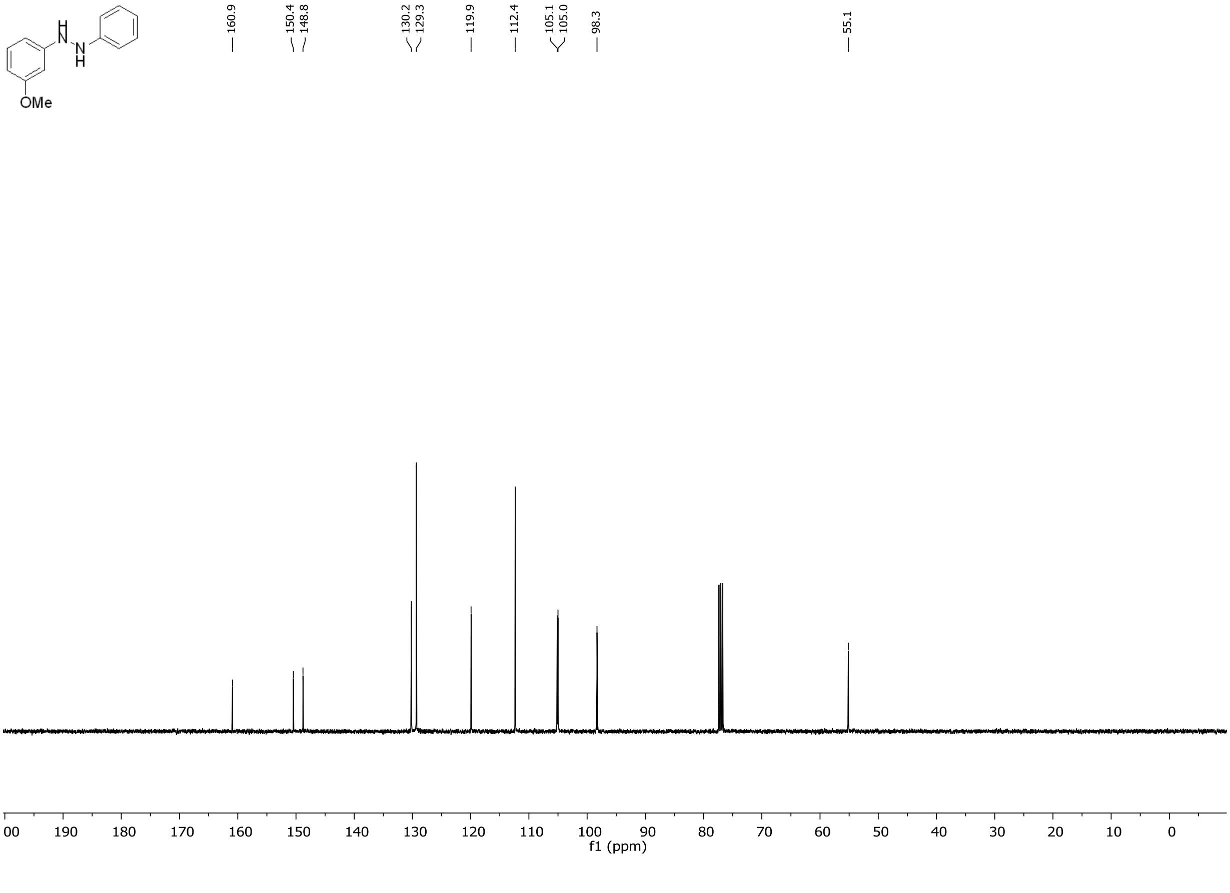

References

(1) Borghs, J. C.; Lebedev, Y.; Rueping, M.; El-Sepelgy, O. Sustainable Manganese-Catalyzed Solvent-Free Synthesis of Pyrroles from 1,4-Diols and Primary Amines. *Org. Lett.* **2019**, *21*, 70–74.

(2) Zubar, V.; Borghs, J. C.; Rueping, M. Hydrogenation or Dehydrogenation of N-Containing Heterocycles Catalyzed by a Single Manganese Complex. *Org. Lett.* **2020**, *22*, 3974–3978.

(3) Mao, Y.; Liu, Y.; Hu, Y.; Wang, L.; Zhang, S.; Wang, W. Pd-Catalyzed Debenzylation and Deallylation of Ethers and Esters with Sodium Hydride. *ACS Catal.* **2018**, *8*, 3016–3020.

(4) Al-Shawabkeh, J. D.; Al-Nadaf, A. H.; Dahabiyeh, L. A.; Taha, M. O. Design, synthesis and structure–activity relationship of new HSL inhibitors guided by pharmacophore models. *Med Chem Res* **2014**, *23*, 127–145.

(5) Katz, C. E.; Aubé, J. Unusual tethering effects in the Schmidt reaction of hydroxyalkyl azides with ketones: cation-pi and steric stabilization of a pseudoaxial phenyl group. *J. Am. Chem. Soc.* **2003**, *125*, 13948–13949.

(6) Baslé, E.; Jean, M.; Gouault, N.; Renault, J.; Uriac, P. Fluorous scavenger for parallel preparation of tertiary sulfonamides leading to secondary amines. *Tetrahedron Lett.* **2007**, *48*, 8138–8140.

(7) Lv, H.; Laishram, R. D.; Li, J.; Zhou, Y.; Xu, D.; More, S.; Dai, Y.; Fan, B. Photocatalyzed oxidative dehydrogenation of hydrazobenzenes to azobenzenes. *Green Chem.* **2019**, *21*, 4055–4061.

(8) Sakai, N.; Fujii, K.; Nabeshima, S.; Ikeda, R.; Konakahara, T. Highly selective conversion of nitrobenzenes using a simple reducing system combined with a trivalent indium salt and a hydrosilane. *Chem. Commun.* **2010**, *46*, 3173–3175.

(9) Singh, S.; Chauhan, P.; Ravi, M.; Taneja, I.; Wahajuddin, W.; Yadav, P. P. A mild CuBr–NMO oxidative system for the coupling of anilines leading to aromatic azo compounds. *RSC Adv.* **2015**, *5*, 61876–61880.

(10) Sharma, S.; Yamini, Y.; Das, P. Hydrogenation of nitroarenes to anilines in a flow reactor using polystyrene supported rhodium in a catalyst-cartridge (Cart-Rh@PS). *New J. Chem.* **2019**, *43*, 1764–1769.

(11) Gao, J.; Bhunia, S.; Wang, K.; Gan, L.; Xia, S.; Ma, D. Discovery of N-(Naphthalen-1-yl)-N'-alkyl Oxalamide Ligands Enables Cu-Catalyzed Aryl Amination with High Turnovers. *Org. Lett.* **2017**, *19*, 2809–2812.

(12) Alsabeh, P. G.; Lundgren, R. J.; McDonald, R.; Johansson Seechurn, C. C. C.; Colacot, T. J.; Stradiotto, M. An examination of the palladium/Mor-DalPhos catalyst system in the context of selective ammonia monoarylation at room temperature. *Chem. Eur. J.* **2013**, *19*, 2131–2141.

(13) Yu, J.; Zhang, P.; Wu, J.; Shang, Z. Metal-free C–N bond-forming reaction: straightforward synthesis of anilines, through cleavage of aryl C–O bond and amide C–N bond. *Tetrahedron Lett.* **2013**, *54*, 3167–3170.

(14) Lee, S.; Jørgensen, M.; Hartwig, J. F. Palladium-catalyzed synthesis of arylamines from aryl halides and lithium bis(trimethylsilyl)amide as an ammonia equivalent. *Org. Lett.* **2001**, *3*, 2729–2732.

(15) Ryabchuk, P.; Junge, K.; Beller, M. Heterogeneous Iron-Catalyzed Hydrogenation of Nitroarenes under Water-Gas Shift Reaction Conditions. *Synthesis* **2018**, *50*, 4369–4376.

(16) Janda, K. D.; Ashley, J. A.; Jones, T. M.; McLeod, D. A.; Schloeder, D. M.; Weinhouse, M. I.; Lerner, R. A.; Gibbs, R. A.; Benkovic, P. A. Catalytic antibodies with acyl-transfer capabilities: mechanistic and kinetic investigations. *J. Am. Chem. Soc.* **1991**, *113*, 291–297.

(17) Ojeda-Porras, A.; Hernández-Santana, A.; Gamba-Sánchez, D. Direct amidation of carboxylic acids with amines under microwave irradiation using silica gel as a solid support. *Green Chem.* **2015**, *17*, 3157–3163.

(18) Xu, H.-J.; Liang, Y.-F.; Cai, Z.-Y.; Qi, H.-X.; Yang, C.-Y.; Feng, Y.-S. CuI-nanoparticles-catalyzed selective synthesis of phenols, anilines, and thiophenols from aryl halides in aqueous solution. *J. Org. Chem.* **2011**, *76*, 2296–2300.

(19) Lu, L.; Ma, J.; Qu, P.; Li, F. Effective Recognition of Different Types of Amino Groups: From Aminobenzenesulfonamides to Amino-(N-alkyl)benzenesulfonamides via Iridium-Catalyzed N-Alkylation with Alcohols. *Org. Lett.* **2015**, *17*, 2350–2353.

(20) Suryavanshi, H. R.; Rathore, M. M. Synthesis and biological activities of piperazine derivatives as antimicrobial and antifungal agents. *Org. Commun.* **2017**, *10*, 228–238.

(21) Wang, M.; Tang, B.-C.; Xiang, J.-C.; Chen, X.-L.; Ma, J.-T.; Wu, Y.-D.; Wu, A.-X. Aryldiazonium Salts Serve as a Dual Synthon: Construction of Fully Substituted Pyrazoles via Rongalite-Mediated Three-Component Radical Annulation Reaction. *Org. Lett.* **2019**, *21*, 8934–8937.

(22) Wang, L.; Ishida, A.; Hashidoko, Y.; Hashimoto, M. Dehydrogenation of the NH-NH Bond Triggered by Potassium tert-Butoxide in Liquid Ammonia. *Angew. Chem. Int. Ed.* **2017**, *56*, 870–873.

(23) Holm, T.; Crossland, I.; Iversen, T.; Jänne, J.; Enzell, C. R.; Rosell, S.; Yanaihara, N.; Yanaihara, C. Mechanisms of the Reactions of Grignard Reagents. XIII. Single Electron Transfer in the Reduction of Azobenzene and Benzophenone. *Acta Chem. Scand.* **1979**, *33b*, 421–428.

(24) Noelting, E.; Werner, P. zur Kenntniss der Diphenylbasen. *Ber. Dtsch. Chem. Ges.* **1890**, *23*, 3252–3266.
